# Nutrient Sources and Transport in the Missouri River Basin, with Emphasis on the Effects of Irrigation and Reservoirs^1^

**DOI:** 10.1111/j.1752-1688.2011.00584.x

**Published:** 2011-08-22

**Authors:** Juliane B Brown, Lori A Sprague, Jean A Dupree

**Keywords:** Missouri River Basin, nutrients, flow modifications, transport, modeling, rivers/streams, lakes, reservoirs, water quality, statistics

## Abstract

**Abstract:**

SPAtially Referenced Regressions On Watershed attributes (SPARROW) models were used to relate instream nutrient loads to sources and factors influencing the transport of nutrients in the Missouri River Basin. Agricultural inputs from fertilizer and manure were the largest nutrient sources throughout a large part of the basin, although atmospheric and urban inputs were important sources in some areas. Sediment mobilized from stream channels was a source of phosphorus in medium and larger streams. Irrigation on agricultural land was estimated to decrease the nitrogen load reaching the Mississippi River by as much as 17%, likely as a result of increased anoxia and denitrification in the soil zone. Approximately 16% of the nitrogen load and 33% of the phosphorus load that would have otherwise reached the Mississippi River was retained in reservoirs and lakes throughout the basin. Nearly half of the total attenuation occurred in the eight largest water bodies. Unlike the other major tributary basins, nearly the entire instream nutrient load leaving the outlet of the Platte and Kansas River subbasins reached the Mississippi River. Most of the larger reservoirs and lakes in the Platte River subbasin are upstream of the major sources, whereas in the Kansas River subbasin, most of the source inputs are in the southeast part of the subbasin where characteristics of the area and proximity to the Missouri River facilitate delivery of nutrients to the Mississippi River.

## Introduction

Agricultural, urban, and industrial development in the Missouri River Basin has increased the demand on limited water resources, particularly in the more arid upper basin, and substantially altered the diverse riverine and floodplain habitats of the Missouri River and its tributaries (National Research Council, 2002; Galat *et al.*, 2005). About 32,000 km^2^ (2.3%) of land is irrigated in the 1,371,000 km^2^ Missouri River Basin (U.S. Department of Agriculture, 2000), and most of the major tributaries have dams, diversion structures, or pump stations to move and store water for irrigation. Numerous dams, canals, and pipelines have also been constructed for flood control, navigation, hydroelectric power generation, and municipal water supply. A series of six reservoirs on the main stem in Montana, North Dakota, South Dakota, and Nebraska constitutes the largest reservoir system in North America, with a storage capacity of 91 km^3^ (Roth, 2005).

Agricultural, urban, and industrial development also has contributed to the nutrient enrichment of streams in the Missouri River Basin (Galat *et al.*, 2005). Various studies (Reckhow and Simpson, 1980; Beaulac and Reckhow, 1982; Kauppi *et al.*, 1993; Johnson *et al.*, 1997; Jones *et al.*, 2004) have reported that nutrient export to streams and water bodies from agricultural and urban-influenced watersheds is substantial although highly variable. Nationally, nutrient enrichment has been identified as one of the leading causes of water-quality impairment in rivers, lakes, and estuaries [U.S. Environmental Protection Agency (USEPA) (2009)]. In the Missouri River Basin, more than 160 stream reaches, lakes or reservoirs, and points were reported to the USEPA for nutrient-related impairment on the 2006 303(d) lists (USEPA Water Quality Assessment and Total Maximum Daily Loads Information, http://www.epa.gov/waters/ir/, *accessed* December 16, 2009). In addition, nutrient loading from the Missouri River Basin and other major tributary basins of the Mississippi River have been linked to a large zone of hypoxia in the Gulf of Mexico (Rabalais *et al.*, 2002; Scavia *et al.*, 2004; Donner and Scavia, 2007; Scavia and Donnelly, 2007; Turner *et al.*, 2008).

Flow modifications (including the application of water to the land surface for irrigation of agricultural fields and storage of water in reservoirs) have previously been shown to affect nutrient fate and transport at other temporal and spatial scales. In several case studies and regional analyses of the effects of irrigation in primary agricultural regions in the United States (U.S.) and in local and regional irrigation-related studies elsewhere (e.g., India, Poland, and the Netherlands), irrigation has been found to increase the leaching of nitrogen to groundwater and streams (Mossbarger and Yost, 1989; Power and Schepers, 1989; Aulakh and Bijay-Singh, 1997; Stites and Kraft, 2001; Domagalski *et al.*, 2008), and surface runoff of sediment-bound phosphorus to streams (McDowell *et al.*, 2001; Westermann *et al.*, 2001; Hansen *et al.*, 2002), as well as to decrease the leaching to groundwater and increase the loss of nitrogen through enhanced denitrification in the soil (Rolston *et al.*, 1982; Goodroad and Keeney, 1984; Sexstone *et al.*, 1985; de Klein and van Logtestijn, 1996; Aulakh and Bijay-Singh, 1997; Mahmood *et al.*, 1998, 2005; Hooda *et al.*, 2003; Vale *et al.*, 2007). In case studies of nutrient attenuation in reservoirs in primary agricultural regions in the U.S., in regional studies in Poland and Sweden, and in a global nitrogen-removal model, reservoirs have been found to retain nutrients and reduce downstream transport through settling of sediment-bound nutrients, denitrification, and uptake by vegetation (Gill *et al.*, 1976; Jansson *et al.*, 1994; Koszelnik and Tomaszek, 2003; David *et al.*, 2006; Harrison *et al.*, 2009).

Improved understanding of how flow modifications such as reservoir storage and irrigation affect nutrient transport in the Missouri River Basin can aid in the refinement of nutrient-management strategies designed to improve the local nutrient conditions in the Missouri River and its tributaries and to reduce nutrient loads leaving the basin. The SPARROW (SPAtially Referenced Regressions On Watershed attributes) model developed by the U.S. Geological Survey (USGS) uses a hybrid statistical and process-based approach to relate instream nutrient load measurements to spatially referenced characteristics of watersheds, including nutrient sources and factors influencing terrestrial and aquatic transport, under long-term steady-state conditions (Schwarz *et al.*, 2006). SPARROW models for nitrogen and phosphorus previously developed for the conterminous U.S. (Smith *et al.*, 1997; Alexander *et al.*, 2000, 2004, 2008) have provided broad insight into nutrient sources and transport in the Missouri River Basin during the late 1980s and early 1990s.

**FIGURE 1:**
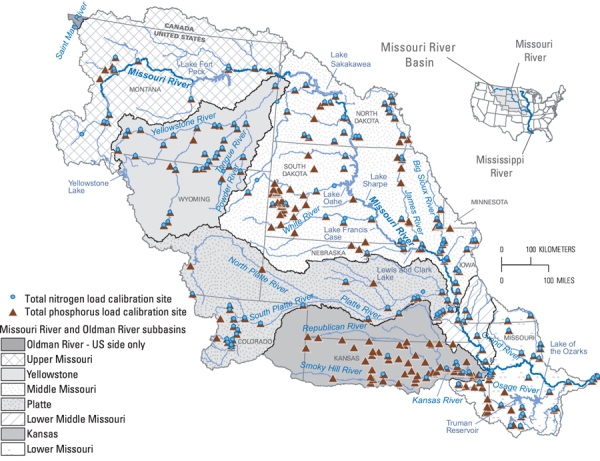
Missouri River Basin Calibration Sites for Total Nitrogen and Total Phosphorus by Subbasin.

We developed refined regional SPARROW total nitrogen (TN) and total phosphorus (TP) models specific to the Missouri River Basin and the U.S. part of the Oldman River drainage (hereinafter referred to as “the Missouri River Basin”) for the early 2000s with a larger number of calibration sites and new or enhanced nutrient source and terrestrial transport variables of regional importance when compared with the 1992 national SPARROW models (Figure 1; Alexander *et al.*, 2008). The selected base time period (early 2000s) was when the most recent periodically reported national and regional-scale geospatial datasets were available (e.g., national land-cover data suitable for large-scale SPARROW modeling were produced only for 1992 and 2002). The base time period helps ensure temporal consistency among geospatial datasets (Preston *et al.*, 2009). The current Missouri River Basin models differ from the most recent national SPARROW models (which had 425 calibration sites nationwide and 57 sites in the Missouri River Basin study area) in several respects, including the change in base year from 1992 to 2002; the change in scale and associated gradients in the source and delivery variables; the more inclusive screening of potential calibration sites; updated datasets (e.g., 2002 *vs.* 1992 data for farm fertilizer, manure, precipitation, and atmospheric deposition); new datasets (e.g., point sources); and the inclusion of more sites with small drainage areas, which increased the variability in observed loads at the calibration sites. The current regional model allows for the potential to identify sources or transport factors that may not have been identified as significant on the national scale. Schwarz *et al.* (2011; this issue) provides an analysis of techniques to regionalize the national model to improve regional estimates.

The Missouri River Basin is one of the eight large geographical regions across the nation (referred to as “major river basins”) identified by the National Water-Quality Assessment (NAWQA) Program of the USGS for assessments of status and trends. The NAWQA Program has integrated the SPARROW-modeling approach in the interpretation of nutrient transport in six of these major river basins (Preston *et al.*, 2009). Results from the Missouri River Basin SPARROW models were used to (1) identify and quantify the major nutrient sources and terrestrial and aquatic delivery factors and processes influencing TN and TP loads in the Missouri River Basin, (2) predict the TN and TP loads (and yields) and track their delivery to the Mississippi River, and (3) evaluate the effects of reservoir storage and irrigated agricultural area on the TN and TP loads.

## Methods

The mathematical form of the SPARROW models is that of a nonlinear regression model in which nutrient loads are related to nutrient-source data weighted by estimates of loss due to terrestrial and aquatic processes (Smith *et al.*, 1997; Schwarz *et al.*, 2006; Alexander *et al.*, 2008). The model includes nonconservative transport, mass-balance constraints, and water flow paths defined by topography, streams, and reservoirs (Alexander *et al.*, 2008). See the Supporting Information (SI; available in online version) for an overview of the model and Schwarz *et al.* (2006) for a detailed description of the SPARROW model.

### SPARROW Model Calibration Data

The major components of the SPARROW model infrastructure are a hydrologic network of stream reaches and associated incremental catchments (the local areas draining directly to a given stream reach), spatial datasets characterizing nutrient sources and landscape and aquatic characteristics, and long-term mean annual load estimates at calibration sites. These model components are described below.

### Hydrologic Network

The hydrologic network dataset used in the Missouri River Basin SPARROW models (MRB_E2RF1) was modified from the 1:500,000-scale Enhanced River Reach File 2.0 reach network for the conterminous U.S. (Nolan *et al.*, 2002; Brakebill *et al.*, 2011; this issue). The MRB_E2RF1 reach network includes attributes that describe the morphology and hydraulic properties of stream reaches (such as estimates of mean discharge, mean velocity, reach length, and travel time) and reservoirs and lakes (such as surface area and outflow discharge). Stream diversions, including canals, ditches, and pipelines, are not included in the MRB_E2RF1 reach network due to their overall complexity and the lack of comprehensive information on location, quantities, and movement within the basins. However, stream divergences, places where the stream channel splits into multiple channels and then rejoins into a single channel, were included. Incremental catchments for the 12,549 stream reaches in the Missouri River Basin (including 73 stream reaches in the U.S. part of the Oldman River drainage that ultimately flow into the Hudson Bay) were delineated from 100-m digital elevation models (Brakebill *et al.*, 2011; this issue). Catchments ranged in size from 0.01 to 6,365 km^2^ (reach catchment sizes: 5th percentile ∼3 km^2^, 95th percentile ∼329 km^2^, and median size ∼68 km^2^). Additionally, 183 reservoirs and lakes (water bodies) in the Missouri River Basin, including two reservoirs in the U.S. part of the Oldman River drainage, were included in the reach network. Of these, approximately 80% are artificial reservoirs and approximately 20% are natural lakes (some of which may be controlled), although no distinction between reservoirs and lakes was made in the Missouri River Basin TN and TP models. The use of the MRB_E2RF1 network resulted in the inclusion of only those reservoirs and lakes connected to medium and larger streams included in the hydrologic network. To maintain hydrologic connectivity in the models, stream reaches in the Canadian portion of the Missouri River Basin were included. However, because comparable nutrient inputs and landscape characteristics were not available for the Canadian drainages, these values had to be estimated. Due to the uncertainty in these estimates, load predictions for reaches in Canada are not reported herein (see “Additional SPARROW model details” section in the SI for more information).

### Nutrient Sources and Landscape and Aquatic Characteristics

Spatial data on nutrient sources and landscape and aquatic characteristics were allocated to the incremental catchment of each stream reach. Some locally important sources or factors affecting nutrient transport may not be included in the final models if they were not found to be regionally significant or if regional- or national-scale datasets were unavailable for evaluation. Unless otherwise noted, all spatial data in this paper are from Wieczorek and LaMotte (2011). For information on point-source data, see Maupin and Ivahnenko (2011; this issue).

Nutrient sources considered during model calibration included point sources (including sewerage and commercial and industrial dischargers); various individual and combined land-cover classes (such as developed, forested, wetland, and range land areas); fertilizer applied to agricultural land; confined and unconfined manure (separate and combined); atmospheric deposition of nitrogen; stream channel contributions; geologic sources; and nitrogen fixation. In some cases, these source variables can serve as surrogates for other nutrient sources that are spatially correlated but that cannot be explicitly modeled. Because SPARROW is a mass-balance model, nutrients are fully allocated across the landscape among the variables specified in the model and therefore some model variables may account for other spatially correlated nutrient sources. For example, developed land area may serve as a surrogate measure of various nonpoint urban sources such as fertilizer, septic leakage, and nitrogen deposition associated with vehicle emissions of nitrous oxides that can enter streams through runoff from impervious surfaces and inflows from groundwater in urbanized catchments (R.B. Alexander, USGS, 2010, written communication). Background sources of TN and TP (e.g., forested land area and geologic sources of phosphorus, respectively) were not significant in the models; therefore, they were likely accounted for in the other sources. Additionally, some sources used in the model may have both natural and anthropogenic origins (e.g., atmospheric deposition).

Land-to-water delivery variables considered included climate variables (precipitation and air temperature); various land area characteristics (such as mean basin slope and elevation, irrigated agricultural land area, land area with tile drains, wetland area, and impervious surface area); bedrock and surficial geology; various soil characteristics (such as permeability, erodibility, soil clay, sand, and silt content; soil organic matter content); stream characteristics (such as channel sinuosity, stream canopy cover, and stream drainage density); and additional flow-related variables (such as base-flow contributions to discharge, overland flow, and number of off-stream reservoirs and lakes).

Aquatic loss in streams was modeled according to a first-order decay process as a continuous function of the time of travel (Schwarz *et al.*, 2006). Additional details on the travel time attributes in the MRB_E2RF1 network are provided in the SI. Because aquatic loss can vary by stream size (Alexander *et al.*, 2000), aquatic loss in streams was evaluated for multiple stream classes defined by discharge (e.g., streams with streamflow greater or less than 100 m^3^/s). These stream classes were adjusted to optimize the model calibration. Aquatic loss (attenuation) in reservoirs and lakes was modeled according to a first-order process as a function of the ratio of areal hydraulic load (estimated as the ratio of outflow discharge to the water-body surface area) to apparent settling velocity (the estimated mean rate at which nutrients move vertically in water) (Schwarz *et al.*, 2006). Instream loss and reservoir and lake attenuation reflect the net balance of processes (e.g., nitrogen fixation, phosphorus resuspension) that supply and remove nutrients from the stream reach or water body (Schwarz *et al.*, 2006). The model assumes that nutrient immobilization and mineralization rates in soils are approximately in equilibrium (Alexander *et al.*, 2008).

### Calibration Sites and Load Estimates

Potential calibration sites included all stream sites in the USGS National Water Information System database and the USEPA Modernized and Legacy STORage and RETrieval (STORET) databases with one or more nutrient records between 1970 and 2005 (Saad *et al.*, 2011; this issue). Additional potential sites were obtained from the databases of agencies that had minimal or no data entry in Modernized STORET, including the U.S. Army Corps of Engineers (USACE), the National Park Service (NPS), the Wyoming Department of Environmental Quality, the Missouri Department of Natural Resources, and the Kansas Department of Health and Environment. Sites within a 100-m radius of one another were identified using Geographic Information Systems buffer tools, and those with unique data records and no intervening reservoirs, diversions, or point-source discharges evident on topographic maps were combined. TN and TP concentrations were compiled for each potential calibration site using either direct measurements or, where possible, a summation of component nutrient species (Saad *et al.*, 2011; this issue). The sites were then screened for sample count and length and coverage of the data record. Because a record of mean daily discharge was required for load estimation at the final calibration sites, sites were also screened for the presence of a co-located or nearby streamgage with contemporaneous data (Saad *et al.*, 2011; this issue). Streamgages operated by the USGS, the NPS, the Montana Department of Natural Resources and Conservation, the Colorado Division of Water Resources, the Minnesota Department of Natural Resources, the U.S. Bureau of Reclamation, and the USACE were screened. In addition to the criteria used for pairing water-quality sites with streamgages (detailed in Saad *et al.*, 2011; this issue), potential water-quality and streamgage pairs that had intervening reservoirs or diversions were excluded from the Missouri River Basin models.

Long-term mean annual loads were then estimated at each remaining potential calibration site using a bias-corrected log-linear regression model with maximum likelihood estimation that relates the concentration to time, discharge, and seasonal terms (Schwarz *et al.*, 2006; Saad *et al.*, 2011; this issue). The use of long-term mean annual loads enhances the ability of the model to identify the major factors that affect the long-term supply, transport, and fate of nutrients in watersheds (Schwarz *et al.*, 2006). The mean annual loads for each site were detrended to a base year of 2002 to give an estimate of the load that would have occurred during 2002 under long-term mean hydrologic conditions (Schwarz *et al.*, 2006; Preston *et al.*, 2009). More detail about the detrending process is available in the SI. Sites where the standard error of the load estimate exceeded 75% of the load estimate were excluded from further consideration, as were sites that could not be indexed to the MRB_E2RF1 reach network or that had more than 50% of their drainage area in Canada. The final number of calibration sites was 193 for TN and 311 for TP (Figure 1). Catchments for the calibration sites ranged from 0.05 to 1,410 km^2^ for TN (reach catchment sizes: 5th percentile ∼1.4 km^2^, 95th percentile ∼293 km^2^, and median size ∼58 km^2^) and 0.06 to 1,531 km^2^ for TP (reach catchment sizes: 5th percentile ∼1.1 km^2^, 95th percentile ∼349 km^2^, and median size ∼61 km^2^). See descriptions and Figures S1, S2a, and S2b for additional geographic details, Figure S5 for TN and TP calibration loads, and Figure S6a (sources) and S6b (land-to-water delivery variables) for comparisons of basin characteristics between calibration sites and all MRB_E2RF1 reaches.

### SPARROW Model Calibration

Model calibration was performed by relating the load data to catchment characteristics at the monitoring sites through nonlinear least-squares regression. The final calibrated SPARROW models for TN and TP were selected on the basis of assessments of the significance (*α* = 0.10) and interpretability (i.e., the variables had to make physical sense) of various specifications of nutrient source, land-to-water delivery, and aquatic loss terms. Model performance was evaluated using a range of statistical diagnostics, such as the coefficient of determination (*R*^2^), magnitude and spatial distribution of residuals, and root-mean-square error (RMSE). The most accurate model (lowest RMSE) with the smallest degree of spatial bias in the residuals was selected as the final model. Approximately 90% confidence intervals for the nonlinear least-squares coefficient estimates were derived using standard methods under the assumption that the least-squares coefficient estimate is normally distributed with standard error equal to the associated nonlinear least-squares standard error estimate (Email from G.E. Schwarz, USGS, to J. B. Brown, USGS, December 29, 2009, Subject: Re: Wording clarification re: CIs and bootstrapping).

The robustness of the final model coefficients (i.e., the ability of the calibrated model coefficients to remain stable with random variations in the input data) for TN and TP was examined using nonparametric resampled bootstrapping techniques with 200 iterations. Nonparametric bootstrapping is based on repeated reestimation of the model applying a different set of randomly selected (with replacement), nonnegative integer weights, each set summing to the number of model observations (Schwarz *et al.*, 2006). Statistical integration of the results of nonparametric bootstrapping provides model parameter estimates that incorporate sampling error and are more robust than the nonbootstrap-derived parameter estimates. Parametric bootstrapping techniques (with 200 iterations) were used to estimate 90% prediction intervals for the load and source-share estimates. Parametric bootstrapping is based on random resampling of the statistical distributions of the nonlinear least-squares parameter estimates, and provides uncertainty estimates that are less biased than either nonlinear least-squares or nonparametric bootstrapping estimates (Schwarz *et al.*, 2006).

### SPARROW Model Output

Model output discussed in this paper includes calibration results and related diagnostics used to evaluate the model, source-share contributions (percentage of load from each source), and mean annual predictions of nutrient mass (load and yield) for stream reaches. In order to compare results across the region, the Missouri River Basin was divided into seven major subbasins, each composed of numerous incremental catchments. The seven subbasins include three tributary subbasins (Yellowstone, Platte, and Kansas River subbasins) and four mainstem Missouri River subbasins that are distributed between the tributary subbasins (Upper, Middle, Lower Middle, and Lower Missouri River subbasins) (Figure 1). Applications of the model output are discussed in the final section of the paper, including an examination of the effects of irrigation and reservoirs and lakes on nutrient transport.

## Results and Discussion

### Calibration Results and Diagnostics

Calibration and bootstrap results for the Missouri River Basin SPARROW models, including parameter estimates, standard errors, and *p*-values, are summarized in Table 1. The TN and TP models explained about 90 and 84%, respectively, of the spatial variability in the log-transformed values of the mean annual load (*R*^2^-load; Table 1). The values of *R*^2^ for load models are generally high because of the strong relation between drainage area and annual discharge, a key determinant of stream load. With adjustment for drainage-area scaling effects to account for variability between calibration sites resulting from differences in drainage area, the models explained about 84 and 68%, respectively, of the variability (*R*^2^-yield; Table 1), which is a better measure of the goodness of model fit for small basins (Schwarz *et al.*, 2006). The RMSE of the TN and TP models was 0.744 and 1.01, respectively (RMSE; Table 1). Nonparametric bootstrapping yielded comparable parameter coefficient estimates, which demonstrates the model's overall robustness (bootstrap coefficient; Table 1).

**TABLE 1:** SPARROW Model Statistics for Total Nitrogen and Total Phosphorus in the Missouri River Basin

			Confidence Interval for Coefficient				
						
Parameters (explanatory variable units)	Calibration Model Coefficient Units	Calibration Model Coefficient	Lower 90%	Upper 90%	Standard Error of Coefficient	Probability Level (*p*-value)*	Nonparametric Bootstrap Estimate of Coefficient (mean)
*Total nitrogen model*							
Nitrogen sources†							
Developed land (km^2^)‡	kg/km^2^/year	511	87.7	934	256	0.024	542
Point sources (kg)	dimensionless	0.962	0.419	1.50	0.328	0.002	0.980
Farm fertilizer (kg)	dimensionless	0.036	0.013	0.059	0.014	0.005	0.034
Manure (kg)	dimensionless	0.040	0.009	0.070	0.019	0.018	0.039
Atmospheric deposition (kg)	dimensionless	0.040	−0.002	0.082	0.025	0.057	0.034
Land-to-water delivery§							
Precipitation (mm)	log (mm)	2.02	1.41	2.63	0.370	<0.001	1.92
Air temperature (°C)	°C	−0.146	−0.201	−0.091	0.033	<0.001	−0.142
Irrigation (km^2^)	percent	−0.058	−0.096	−0.021	0.023	0.011	−0.062
Loess (km^2^)	percent	0.013	0.008	0.018	0.003	<0.001	0.014
Aquatic loss¶							
Instream loss (*Q* < 3.1 m^3^/s) (m/day)	(days)^−1^	0.150	0.056	0.244	0.057	0.004	0.154
Reservoir and lake attenuation (m/year)	m/year	10.5	3.33	17.7	4.36	0.008	9.52
MSE		0.553					
RMSE		0.744		R-squared load			0.903
Number of observations		193		R-squared yield			0.839
*Total phosphorus model*							
Phosphorus Sources†							
Developed land (km^2^)‡	kg/km^2^/year	32.3	10.6	53.9	13.1	0.007	29.4
Point sources (kg)	dimensionless	0.86	0.32	1.4	0.327	0.004	0.832
Farm fertilizer (kg)	dimensionless	0.011	0.002	0.02	0.005	0.027	0.011
Manure (kg)	dimensionless	0.009	0.003	0.015	0.004	0.006	0.008
Stream channels (reach lengthwhere *Q* > 1.13 m^3^/s) (m)	kg/m/year	0.176	0.119	0.233	0.034	<0.001	0.182
Land-to-water delivery§							
Precipitation (mm)	log (mm)	2.33	1.74	2.91	0.354	<0.001	2.43
Soil permeability (cm/h)	log (cm/h)	−1.13	−1.55	−0.71	0.253	<0.001	−1.11
Mean basin slope (percent)	Percent	0.096	0.058	0.133	0.023	<0.001	0.096
Aquatic loss¶							
Reservoir and lake attenuation (m/year)	m/year	39.3	18.7	59.9	12.5	0.001	37.9
MSE		1.02					
RMSE		1.01		R-squared load			0.838
Number of observations		311		R-squared yield			0.681

Notes: °C, degrees celsius; cm/h, centimeters per hour; kg, kilograms; kg/km^2^/year, kilograms per square kilometer per year; kg/m/year, kilograms per meter per year; km^2^, square kilometers; m, meters; mm, millimeters; m^3^/s, cubic meters per second; m/d, meters per day; MSE, mean-square error; m/year, meters per year; Q, mean annual discharge; RMSE, root-mean-square error, <, less than.

* The reported *p*-values are one-sided values for the source and aquatic-loss variables, which were constrained in the model to be positive resulting in a one-sided hypothesis test to evaluate the statistical evidence of the importance of these variables in the model. The reported *p*-values are two-sided for the land-to-water delivery variables, which should generally not have a prior expectation as to the nature of the physical relation to the load and should be evaluated in terms of physical meaningfulness as part of the model calibration process.

† The source coefficients measure the mean rate of nutrient mass delivered to streams as a function of the source input units. Source-related coefficients based on source inputs expressed in areal units (e.g., developed land and stream channels) describe the mass per unit area delivered to streams from these areas. The point-source coefficient estimate, which is a direct measure of point-source loading to the stream (and are in the same units as the response variable) is expected to be close to 1.0 (1.0 should be contained within the confidence interval). The sources with dimensionless coefficients (e.g., farm fertilizer) provide a measure of the fraction of nutrient that is delivered from each source to the aquatic system and can be evaluated as a percent of input from the source that is delivered to the aquatic system (Schwarz *et al.*, 2006).

‡ Developed land from 2001 National Land Cover Database (NLCD, http://www.mrlc.gov/nlcd.php, *accessed* April 21, 2011) includes developed open space, and low-, medium-, and high-intensity developed land areas (original units in square kilometers). See Supporting Information for more information.

§ Delivery variables are standardized to improve the interpretability and to provide stability in their values across alternative land-to-water delivery factor specifications by expressing them as differences from their regional mean value over all the reaches.

¶ Aquatic loss variables are in units per time and can be directly interpreted without standardization. For example, the total nitrogen loss for streams with discharge <3.1 m^3^/s = 0.150 or 15% removal of nitrogen per day of water travel time. The rate coefficients are applied in first-order mass-transfer rate expressions in the model. For streams, the quotient of the rate coefficient and mean water depth quantifies the rate of nutrient loss per unit of water travel time. The reservoir and lake rate coefficient (*k*^r^) and the areal hydraulic load (*q*^r^; ratio of water-body discharge to surface area) are used in the expression 1/(1 + *k*^r^(*q*^r^)^−1^) to quantify the proportion of nutrient mass transported through water bodies (reservoirs and lakes).

**FIGURE 2:**
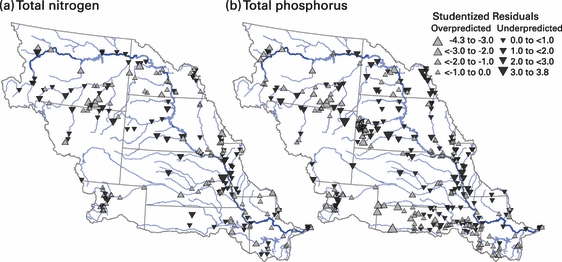
Model Residuals for Sites Used to Calibrate the SPARROW Models of (a) Total Nitrogen and (b) Total Phosphorus. The residuals are expressed in standardized units for the standard normal distribution (Schwarz *et al.*, 2006).

Models were evaluated for evidence of local or regional prediction biases on the basis of visual inspections of calibration-site residual maps (Figure 2). Residuals are expressed as studentized to normalize the residuals allowing for the identification of the largest over and underpredictions (defined as <−2 and >2, respectively). Between 5 and 6% of the residuals were <−2 or >2 for the TN and TP models. For the TN model, there was a slight tendency for underprediction at sites in the upper Powder River Basin and overprediction at sites in the Missouri River downstream from the confluence with the Little Missouri River, in the Platte River Basin in eastern Colorado and Nebraska, and in the Tongue River Basin in the Yellowstone River Basin (Figure 2a). For the TP model, there was a slight tendency for underprediction at sites in the upper James River Basin and overprediction for sites in the Tongue River Basin and in the headwaters of the Kansas River Basin (Figure 2b). Loads tended to be underpredicted on the mainstem and the south-flowing tributaries to the Missouri River downstream from Omaha, Nebraska, in both models. Plots of the observed loads against predicted loads and observed yields against predicted yields suggest reasonably unbiased models, although values are more variable for sites with small loads; generally, there are few sites with very small or very large loads (see Figures S3, S4a, and S4b). Slightly larger variation is evident for TP loads when compared with TN loads, which is consistent with the higher RMSE (and lower *R*^2^) of the TP model and with previously published regression-based nutrient models (Smith *et al.*, 1997; Moore *et al.*, 2004; Alexander *et al.*, 2008; Spahr *et al.*, 2009). Plots of the prediction loads and yields against residuals indicate that the residuals were approximately homoscedastic (i.e., equal variance) (see Figures S3, S4c, and S4d).

Water transfers by diversions, canals, and pipelines in the Missouri River Basin substantially affect water routing throughout the study area; these transfers are not well represented in the reach network due to their complexity and incomplete flow records. Inaccurate accounting of water diversions in the reach network may have had a substantial effect on the calibration sites with small drainage areas and low total streamflow. Smith *et al.* (1997) also attributed the larger positive and negative residuals west of the Mississippi River in their nutrient models to transport measurement errors. Other factors affecting residuals and overall model accuracy may be sparseness of calibration sites and basin heterogeneity.

### Nutrient Sources

Nutrient sources included in the TN model were point sources, developed land (nonagricultural), farm fertilizer, manure (from confined plus unconfined livestock), and atmospheric deposition (Table 1). Nutrient sources included in the TP model were point sources, developed land, farm fertilizer, manure (from confined plus unconfined livestock), and stream channels (using stream length in reaches with mean discharge >1.13 m^3^/s as the model variable) (Table 1). Nutrient sources are described in more detail and mapped in the SI (see Figures S2 and S7-S12 for TN and Figures S2, S7-S11, and S13 for TP).

SPARROW coefficients for the nutrient sources provide an estimate of the fraction (for source inputs estimated in mass, or kg) or absolute quantity (for source inputs measured in area, or km^2^) of each source input that is delivered to streams (Schwarz *et al.*, 2006). As expected, nearly all of the inputs from point sources, those that discharge directly to streams and thus are unaffected by land-to-water delivery factors, were estimated to be delivered to streams, as indicated by the model coefficients near 1. Parameter estimates for point sources should approximate 1 if all sources are described accurately and losses are adequately accounted for by land-to-water delivery factors (Schwarz *et al.*, 2006). Point-source parameter estimates less than (or greater than) 1 indicate that point-source loads were underestimated (or overestimated); the Missouri River Basin estimates were 0.962 (for TN) and 0.86 (for TP), with 1 included within the standard error of both model estimates (Table 1).

A substantially smaller fraction of nonpoint-source nutrient inputs on the land surface was estimated to be delivered to streams – on average, about 3.6 to 4% of the nitrogen inputs from fertilizer, manure, and atmospheric deposition and about 1% of the phosphorus inputs from fertilizer and manure. These estimates are based on a theoretical average catchment condition; delivery in any single catchment varies depending on the particular land-to-water delivery factors in that catchment. Because nutrients reaching the streams from these nonpoint sources have been subject to loss through natural processes (e.g., plant uptake and denitrification), the coefficients of these sources reflect a much lower delivery fraction than nutrients delivered from point sources. The estimated fractions of nitrogen and phosphorus inputs from fertilizer, manure, and atmospheric deposition delivered to streams in the Missouri River Basin were lower than those in some national and regional models (Alexander *et al.*, 2008; Preston *et al.*, 2011; this issue) but comparable to those in others (Wise and Johnson, 2011; this issue; Rebich *et al.*, 2011; this issue).

**FIGURE 3:**
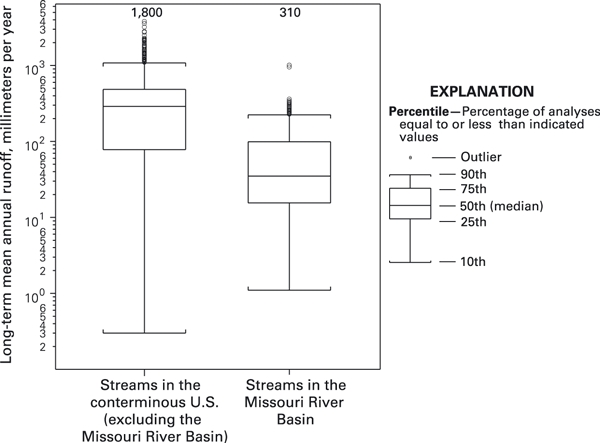
Comparison of Long-Term Mean Annual Runoff (from 1975 to 2007) to Streams in the Missouri River Basin and to Streams in Other Parts of the Conterminous United States. (Streams are aggregated at the 8-digit Hydrological Unit Code (HUC8) level for these runoff estimates.)

Runoff to streams, estimated here as the long-term mean annual runoff from 1975 to 2007 (Brakebill *et al.*, 2011; this issue), is significantly lower in the Missouri River Basin than in other parts of the conterminous U.S. (Wilcoxon rank-sum test, *p*< 0.0001) (Figure 3). This may be one explanation for the relatively low delivery of nutrients to streams in the Missouri River Basin. Calculations of 41-year average annual runoff from 1948 to 1988 for the conterminous U.S. by Dolph and Marks (1992) similarly indicated that runoff across the central part of the Missouri River Basin was low (1-5 cm) compared with estimates for most of the remaining area in the conterminous U.S. (ranging from about 5 to >300 cm), except for areas in the southwestern U.S., which were also low (1-10 cm). Galat and Lipkin (2000) and Galat *et al.* (2005) indicated that spatiotemporal patterns in runoff and hydrology including relatively low precipitation and losses from evapotranspiration in the semiarid Great Plains are the dominant factors in overall low runoff to the Missouri River.

The inclusion of stream channels as a phosphorus source in the TP model indicates that the channels of medium and larger streams (approximately 34% of streams in the study area) are a net source of phosphorus in the Missouri River Basin (see Figure S13). Sources of this channel-derived phosphorus may be geologic and (or) anthropogenic from adjacent and upstream areas in the basin; for example, some of these streams are in areas of intensive agriculture or are underlain by the Phosphoria Formation, which is a source of phosphorus-rich deposits (Gulbrandsen, 1966; Raines *et al.*, 1996; Hein, 2004; Stoeser *et al.*, 2007). Phosphorus attached to sediment from channel erosion and scouring was found to be a significant source in the Lower Mississippi River and Texas-Gulf TP SPARROW model (Rebich *et al.*, 2011; this issue). Stream channels were also found to be a significant source of sediment in a recent national application of the SPARROW model of suspended sediment (Schwarz, 2008). The correspondence between these models suggests that a likely source of phosphorus in the medium and larger stream channels is mobilized sediment. Phosphorus flushed into stream channels during high flows and subsequently deposited or sorbed to streambed sediments during lower flows can be resuspended during high-flow events or desorbed when phosphorus in the overlying stream is out of equilibrium, which typically occurs during periods of lower flows (Meyer, 1979; McDowell *et al.*, 2001; Owens *et al.*, 2001; Owens and Walling, 2002; Jarvie *et al.*, 2005; van der Perk *et al.*, 2006). Because the SPARROW model represents long-term steady-state conditions, the inclusion of the stream channel as a net source of phosphorus indicates that channel erosion and scouring may not be in long-term equilibrium with deposition in the Missouri River Basin. Small streams (defined as reaches in which mean streamflow is ≤1.13 m^3^/s) were not found to be significant sources of phosphorus in the TP model; velocities in these smaller streams may not be great enough to resuspend substantial amounts of sediment.

### Land-to-water Delivery

The delivery of TN and TP to streams in the Missouri River Basin was influenced by both climatic and land-surface characteristics (Table 1). Land-to-water delivery factors that influenced TN delivery to streams include precipitation, air temperature, percentage of irrigated agricultural acreage in an incremental catchment, and percentage of loess in an incremental catchment. Land-to-water delivery factors that influenced TP delivery to streams included precipitation, soil permeability, and mean basin slope. Land-to-water delivery factors are described in more detail and mapped in the SI (Figures S14-S17 for TN and Figures S14, S18, and S19 for TP).

Three land-to-water delivery variables with negative coefficients – soil permeability (TP model), air temperature (TN model), and percentage of irrigated agricultural acreage (TN model) – attenuate the transport of nitrogen or phosphorus to streams in the Missouri River Basin. Permeable soils facilitate infiltration rather than runoff, thereby reducing the amount of phosphorus reaching streams (Heathwaite and Dils, 2000; Sharpley *et al.*, 2001; Djodjic *et al.*, 2004). Higher ambient air temperatures can increase the rate of biological processes such as denitrification that reduce the amount of nitrogen reaching streams (Sabey *et al.*, 1954; Kowalenko and Cameron, 1976; Myrold, 2004).

The negative coefficient for irrigated agricultural acreage in the TN model indicates that irrigation reduces the amount of nitrogen transported to streams in the Missouri River Basin. At small scales, irrigation has previously been found to increase the leaching of nitrogen to groundwater (Mossbarger and Yost, 1989; Power and Schepers, 1989; Aulakh and Bijay-Singh, 1997; Stites and Kraft, 2001); nitrogen in groundwater may then be transported to streams, potentially increasing concentrations in those streams. Other studies, however, have found that denitrification rates in the soil substantially increase with irrigation (Rolston *et al.*, 1982; Sexstone *et al.*, 1985; de Klein and van Logtestijn, 1996; Aulakh and Bijay-Singh, 1997; Mahmood *et al.*, 1998, 2005; Hooda *et al.*, 2003; Vale *et al.*, 2007), ultimately resulting in a decrease in concentrations in streams. Enhanced denitrification during irrigation is in part a result of the increase in the underlying anaerobic zones formed during periods of higher soil moisture (Smith and Arah, 1990; Monnett *et al.*, 1995; Domagalski *et al.*, 2008). The significant negative coefficient for irrigated acreage in the TN model indicates that denitrification losses outweigh leaching to groundwater on a basin scale. Because the SPARROW coefficients represent average basin conditions and because factors such as climate; soil characteristics including moisture, structure, temperature, texture, oxygen concentrations, pH, and organic carbon content; and fertilizer application can affect denitrification rates (Groffman and Tiedje, 1988; Pfenning and McMahon, 1996; Follett, 2008), it is possible that leaching may be increasing TN transport at a small scale in some parts of the Missouri River Basin. The structure of the SPARROW model, however, does not allow for small-scale changes in the effects of the land-to-water delivery factors. Additionally, water that is imported from outside of the immediate drainage basin for irrigation (as often occurs in the upper Missouri River Basin states) may contain lower nutrient concentrations than native water, potentially diluting and decreasing nutrient concentrations in the receiving streams (Domagalski *et al.*, 2008). However, irrigated acreage was not a significant land-to-water delivery variable for the TP model; this lack of correspondence between the TN and TP models suggests that dilution from imported irrigation water was not a major factor for either TN or TP transport on a basin scale.

The remaining land-to-water delivery variables with positive coefficients – precipitation (TN and TP models), percentage of loess (TN model), and mean basin slope (TP model) – enhance the transport of one or both nutrients to streams (Table 1). Precipitation affects the amount and rate of overland flow to streams and recharge to groundwater. Several regional and national models have identified precipitation as a significant variable that enhances the delivery of nitrogen and phosphorus to streams (Smith *et al.*, 1997; Alexander *et al.*, 2008; Rebich *et al.*, 2011; this issue; Wise and Johnson, 2011; this issue). Loess is typically a nonstratified, porous, highly erosive sediment that commonly stands in steep or vertical faces and consists predominantly of wind-transported silt with variable amounts of sand and clay loosely cemented by calcium-carbonate (McNab *et al.*, 2005). In loess areas where conservation practices (e.g., terracing) have been incorporated to reduce erosion, infiltration and subsequent transport of nitrogen to the stream through groundwater may prevail; in loess areas where conservation practices are absent, surface erosion and subsequent overland transport of nitrogen to the stream may be most common (Saxton *et al.*, 1971; Burwell *et al.*, 1976, 1977). The presence of thick loess soils was one factor likely influencing higher nitrate export in a case study comparison of three adjacent agricultural watersheds in Ohio and Indiana (Vanni *et al.*, 2001), whereas studies by Kalkhoff (1995) and Steinheimer *et al.* (1998) found that nitrogen concentrations were positively correlated with the quantity of loess in the watersheds. Studies on the Loess Plateau in China show large losses of nutrients from soil erosion attributable to land use, presence of loess, and slope (Meng *et al.*, 2008). Although phosphorus transport is also typically enhanced in areas with highly erosive sediment, the percentage of loess was not significant in the TP model, perhaps in part because other factors reflecting erosion potential (such as mean basin slope) were incorporated into the model. As basin slope increases, erosion can intensify, increasing the delivery of sediment-bound phosphorus to streams (Elkholm *et al.*, 2000; Kronvang *et al.*, 2003; Laubel *et al.*, 2003; Meng *et al.*, 2008).

### Aquatic Loss

The instream loss rate for the TN model was estimated to be 0.150 day^−1^ for streams with a mean annual discharge ≤3.1 m^3^/s (Table 1). Instream attenuation of nitrogen for streams with discharges >3.1 m^3^/s was not significant, indicating that such loss is minimal in larger streams. These findings are consistent with results of national and other regional SPARROW models (Smith *et al.*, 1997; Preston and Brakebill, 1999) as well as other studies (Peterson *et al.*, 2001) and are likely related to greater light penetration, algal activity, and contact with benthic sediments in smaller, shallower streams, resulting in a greater potential for biologically driven nutrient uptake and sedimentation (Stream Solute Workshop, 1990; Howarth *et al.*, 1996; Alexander *et al.*, 2000; Seitzinger *et al.*, 2002; Bernot and Dodds, 2005; Mulholland *et al.*, 2008). For comparison to reservoir and lake attenuation, the aquatic loss rate was converted into a rate representing nutrient loss per unit of water travel time by multiplying 0.150 day^−1^ by mean stream depth, for a value of 0.059 m/day (22 m/year). The estimated loss rate for nitrogen in reservoirs and lakes (10.5 m/year) was approximately half the rate estimated in streams. The greater supply of organic matter commonly available in streams can result in more denitrification (Howarth *et al.*, 1996), although Saunders and Kalff (2001) found that differences in the proportion of N attenuated in wetlands, lakes, and rivers were almost entirely explained by differences in discharge.

Instream loss of phosphorus was not significant for streams of any size in the Missouri River Basin (Table 1). Streams in the basin do not appear to be net sinks for phosphorus; indeed, the inclusion of stream channels as a source term in the TP model indicates that some streams (those with discharges >1.13 m^3^/s) are a net source of phosphorus. This finding is further supported by research that suggests storage and subsequent re-release of bed-sediment phosphorus is an important and dynamic process governing the export of phosphorus from some catchments (Jarvie *et al.*, 2005). In simulations made with previous national SPARROW models, however, streams were not found to be a net source of phosphorus, but rather, streams of various sizes were found to be net sinks (Alexander *et al.*, 2004, 2008), raising the possibility that some aspects of phosphorus cycling in the Missouri River Basin are atypical of some other areas of the U.S. The estimated loss rate for phosphorus in reservoirs and lakes in the Missouri River Basin was 39.3 m/year, more than three times the estimated loss rate for nitrogen in water bodies in the basin. Throughout the Missouri River Basin, TP attenuation in reservoirs and lakes was significantly higher than TN attenuation (Wilcoxon signed-rank test, *p*< 0.0001). Similar results have been reported in other studies (Gill *et al.*, 1976; Jossette *et al.*, 1999). Higher TP attenuation is likely due to the tendency of phosphorus to attach to sediment and for water bodies to act as sediment traps (Dendy, 1968; Stumm and Morgan, 1970; McHenry, 1974). The estimated rate of phosphorus loss in reservoirs and lakes in the Missouri River Basin is comparable to recent national estimates reported by Alexander *et al.* (2008) for phosphorus (34 m/year) and by Schwarz (2008) for sediment (36.5 m/year) and regional estimates for the Southeastern U.S. reported by García *et al.* (2011; this issue) for phosphorus (31.5 m/year), although it is higher than earlier estimates of phosphorus loss in reservoirs made by Alexander *et al.* (2004) for the U.S. (14.3 m/year) and somewhat larger than some of the recent regional SPARROW models and typical literature estimates for TP loss in reservoirs, which range from about 4 to 20 m/year (Chapra, 1997; Rebich *et al.*, 2011; this issue; Robertson and Saad, 2011; this issue).

### Prediction Results

In this study, predicted loads are summarized for the Missouri River Basin and its major subbasins using *incremental* catchments (the local areas draining directly to a given stream reach) and *total* catchments (the incremental catchment plus all of the upstream catchments draining to a stream reach). Information discretized by incremental catchment preserves detail on the spatial distribution of source and transport attributes, whereas information accumulated over the total catchment enables the comparison of conditions among the major subbasins. For both incremental and total catchments, the load (the load in the stream reach transported to the reach outlet after accounting for the effects of instream attenuation) and the *delivered* load (that portion of the load ultimately delivered to the Mississippi River) are presented. To compare catchments of different size, yields (loads normalized to catchment area) are also discussed.

### Incremental Catchments: Loads and Yields

**FIGURE 4:**
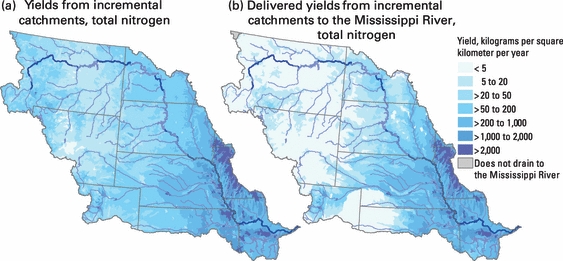
Model Estimates of Total Nitrogen (a) Yields and (b) Yields Delivered to the Mississippi River From Incremental Catchments in the Missouri River Basin.

**FIGURE 5:**
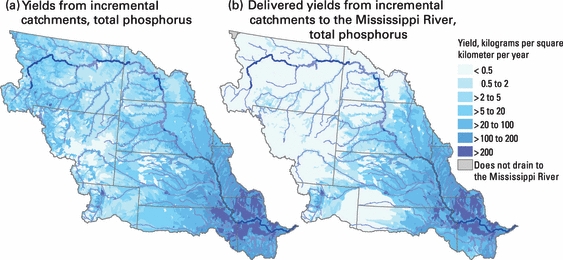
Model Estimates of Total Phosphorus (a) Yields and (b) Yields Delivered to the Mississippi River From Incremental Catchments in the Missouri River Basin.

The TN yields generally ranged from 13 (10th percentile) to 730 (90th percentile) kg/km^2^/year, with a median of 55.7 kg/km^2^/year, for incremental catchments in the Missouri River Basin (Table 2). The TP yields generally ranged from 0.630 (10th percentile) to 163 (90th percentile) kg/km^2^/year, with a median of 10.8 kg/km^2^/year, for incremental catchments in the Missouri River Basin. Yield estimates outside of this range occur in catchments not well represented by calibration sites (e.g., catchments of very small size or with very high precipitation). Yields in incremental catchments are the amounts of locally generated TN and TP in the stream reaches as illustrated in Figures 4a and 5a, respectively. Yields of TN and TP in incremental catchments generally increased from west to east, although the pattern was less distinct for phosphorus than for nitrogen. High yields of TN and TP (generally >200 kg/km^2^/year for TN and >20 kg/km^2^/year for TP) in the southeast part of the basin likely reflect agricultural and urban activities (Figure S2), whereas high yields of TP (generally between 5 and 100 kg/km^2^/year) in the headwaters of the Upper Missouri and Yellowstone River subbasins likely reflect the relatively high contribution from stream channels (Figure S13). The areas with the lowest yields of TN and TP (generally ≤50 kg/km^2^/year for TN and ≤5 kg/km^2^/year for TP) were in the southwestern headwaters of the Yellowstone River subbasin; additional areas with relatively low yields included the headwaters of the Platte and Middle Missouri River subbasins and the central and eastern areas of the Yellowstone and Upper Missouri River subbasins. These areas are generally characterized by fewer and smaller point sources, less developed land, and low fertilizer inputs when compared with other parts of the Missouri River Basin (Figures S7-S10). The spatial distribution and magnitude of incremental yields are generally comparable to the values reported by Alexander *et al.* (2008) and presented by Robertson *et al.* (2009) for the Missouri River Basin in a study ranking nutrient yields from the Mississippi/Atchafalaya River basins.

**TABLE 2:** Summary Statistics of Yields and Source Shares From Incremental Catchments in the Missouri River Basin

	Total Nitrogen (TN)							Total Phosphorus (TP)						
		
Variable	Mean	SD	10th	25th	Med	75th	90th	Mean	SD	10th	25th	Med	75th	90th
Yield^1^ (kg/km^2^/year)														
Incremental^1^	334	4,197	13	25.3	55.7	196	730	67.7	400	0.630	1.90	10.8	70.8	163
Delivered incremental^2^	258	4,180	0.532	1.82	6.49	77.6	619	38	375	0.002	0.017	0.222	12.1	105
Source shares (%)^3^														
Developed land	13.0	14.6	0	2.12	10.0	16.8	28.7	12.1	16.2	0	0.218	7.83	16.9	28.6
Point sources	1.49	9.52	0	0	0	0	0	1.57	9.71	0	0	0	0	0
Farm fertilizer	28.0	23.8	0	2.83	25.6	49.7	61.3	21.7	22.5	0	0.698	13.8	39.6	57.2
Manure	34.4	19.5	10.9	18.6	31.6	49.2	62.0	41.9	33.8	1.1	11.6	35.4	69.2	98.3
Atmospheric deposition	23.2	19.3	6.57	9.92	16.5	30.7	48.3	-	-	-		-		-
Stream channels	-	-	-		-		-	22.8	37.8	0	0	0	36.3	96.6

Notes: kg/km^2^/year, kilograms per square kilometer per year; Med, median (50th percentile); SD, standard deviation.

1 The amount of TN or TP generated within a given incremental catchment that is delivered to the catchment outlet after accounting for the effects of instream attenuation processes associated with one-half the reach time of travel.

2 The amount of TN or TP generated within a given incremental catchment that is ultimately delivered to the Mississippi River.

3 The amount (share) of TN or TP, in percent, generated within a given incremental catchment that can be attributed to the sources in the model.

On average, the largest sources of nitrogen in the incremental catchments were manure (34.4%), farm fertilizer (28.0%), and atmospheric deposition (23.2%), whereas the largest sources of phosphorus were manure (41.9%), stream channels (22.8%), and farm fertilizer (21.7%) (Table 2). In contrast, on average, point sources contributed <2% for both nitrogen and phosphorus, whereas developed land contributed between 12 and 13%. Alexander *et al.* (2004) showed similar distributions between agricultural and urban sources (78 and 13%, respectively) of phosphorus in the Missouri River Basin. The contributions from each source ranged from at or near zero to >90% in individual incremental catchments, reflecting the variability in predominant inputs throughout the Missouri River Basin (Table 2; Figures S7-S11). The inclusion of medium-sized and larger stream channels as a source of TP in the Missouri River Basin is somewhat unique for SPARROW nutrient models, although Rebich *et al.* (2011; this issue) also found stream channels as a significant source of TP in the Lower Mississippi River and Texas-Gulf region. In related studies on channel sources of phosphorus, Meyer (1979) evaluated the role of streambed sediments in phosphorus dynamics for a small undisturbed catchment and found that sediments provide a continuously renewed source of phosphorus. However, Walling *et al.* (2003) found that the phosphorus from streambed sediment represented only a small fraction of the TP load (0.6-1.2%) in the Aire and Swale Rivers evaluated in Yorkshire, United Kingdom.

**FIGURE 6:**
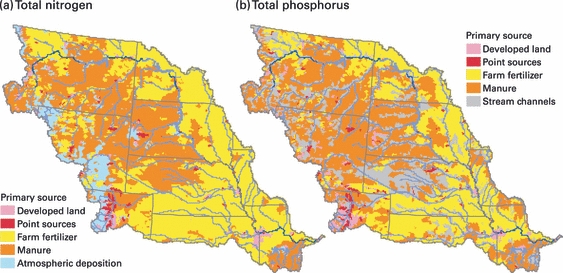
Primary Sources of (a) Total Nitrogen and (b) Total Phosphorus Within an Incremental Catchment in the Missouri River Basin. (For this figure, the primary source in each of the 12,549 incremental catchments was defined as the source contributing the greatest percentage of the total load in the incremental catchment. Other sources are also usually present in each catchment, but in smaller proportions.)

Agriculture contributed the largest relative percentage of the nutrient load generated within each incremental catchment throughout a large part of the Missouri River Basin (Figure 6). Manure was the predominant source of TN and TP in incremental catchments in the north-central part of the study area, including nearly the entire Yellowstone River subbasin, the western section of the Middle Missouri River subbasin, and a smaller area in the southern part of the Lower Missouri River subbasin. Farm fertilizer was the predominant source of TN and TP in many of the remaining incremental catchments in the southern and eastern parts of the study area, as well as in some of the incremental catchments along the northern basin boundary in Montana and North Dakota. Agricultural sources were also found to be the predominant sources of TN and TP in the Missouri River Basin by Alexander *et al.* (2008). Developed land and point sources were the predominant sources of TN and TP in incremental catchments concentrated in the areas surrounding Denver, Colorado; Helena, Montana; and Kansas City, Missouri; and to a lesser extent, Omaha; Rapid City, South Dakota; and Jefferson City, Missouri (see Figure S1 for area locations). Atmospheric deposition was the predominant source of TN largely along the western boundary of the basin; this finding is consistent with the limited agricultural and urban development in these northern and central Rocky Mountain headwater catchments (see source variable figures and descriptions in SI). The incremental catchments with stream channels as the predominant source of TP were more scattered throughout the study area, although those were fewer in the southeastern part of the basin.

### Incremental Catchments: Yields Delivered to the Mississippi River

The median yield ultimately delivered to the Mississippi River from all of the incremental catchments in the Missouri River Basin was 6.49 kg/km^2^/year for TN and 0.222 kg/km^2^/year for TP (Table 2). The yield ultimately delivered to the Mississippi River from each incremental catchment is illustrated in Figures 4b and 5b. Delivery from incremental catchments was greatest in the southeast part of the basin (generally >1,000 kg/km^2^/year of TN and >100 kg/km^2^/year of TP), where relatively high precipitation, the presence of loess-dominated geologic units, low soil permeability, and proximity to the Mississippi River contributed to a large amount of the nutrient yield being delivered to the Mississippi River. A limited amount of the nutrient yield was delivered to the Mississippi River from the northwestern and western parts of the basin (generally ≤50 kg/km^2^/year of TN and ≤5 kg/km^2^/year of TP) because of the nitrogen attenuation in these smaller headwater streams (Howarth *et al.*, 1996; Alexander *et al.*, 2000), long travel time to the Mississippi River, and attenuation in numerous reservoirs and lakes, including the large mainstem reservoirs on the upper and middle Missouri River. One exception was the Platte River subbasin, where a relatively large amount (>2,000 kg/km^2^/year of TN and >200 kg/km^2^/year of TP from some incremental catchments) of the nutrient yields generated in the headwaters of the Platte River Basin near Denver were delivered to the Mississippi River. The distinctive delivery pattern in this subbasin is likely due to the geographic distribution of water bodies on the Platte River, most of which, including the largest reservoirs, are upstream from the major point and agricultural sources. These values are comparable in relative magnitude and geographic distribution to estimates of delivery from incremental catchments in the Missouri River Basin to the Gulf of Mexico made by Alexander *et al.* (2000) using a 1987 base year (generally <1,200 kg/km^2^/year for TN) and by Alexander *et al.* (2008) and reported by Robertson *et al.* (2009) using a 1992 base year (generally <1,000 kg/km^2^/year for TN and <100 kg/km^2^/year for TP).

### Total Catchments: Loads and Yields in the Major Subbasins

**FIGURE 7:**
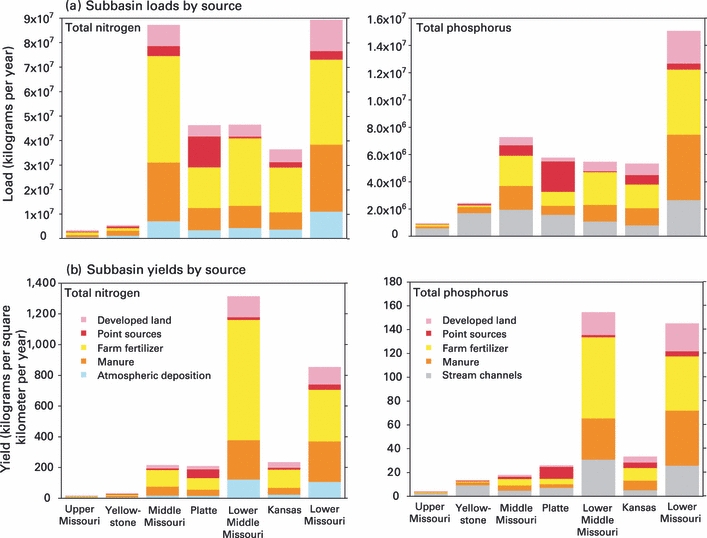
Distribution of (a) Loads and (b) Yields by Source Delivered to the Subbasin Outlet From the Total Catchments of the Major Subbasins for Total Nitrogen and Total Phosphorus. (Source-share contributions for all subbasins are available in Table S5 in SI. Note: relative source-share contributions are the same for load and yield in a subbasin.)

Loads and yields of TN and TP varied considerably among the total catchments of the major subbasins of the Missouri River Basin. Loads and yields for each subbasin – the load or yield in the incremental catchment at the subbasin outlet plus the loads or yields from all of the upstream catchments that are delivered to the subbasin outlet – are illustrated in Figure 7 by source for TN and TP.

Because of the large size of the Middle Missouri River subbasin and the relatively high inputs in the Lower Missouri River subbasin, the largest TN and TP loads were in these two subbasins, although the TP loads from the Platte, Lower Middle Missouri, and Kansas River subbasins were nearly as large as the TP load from the Middle Missouri River subbasin (Figure 7a). The smallest loads were from the Upper Missouri and Yellowstone River subbasins where inputs from all sources are low when compared with inputs from the other subbasins.

The smallest nutrient yields (<50 kg/km^2^/year for TN and <20 kg/km^2^/year for TP) were in the Upper Missouri River and Yellowstone River subbasins, whereas the largest yields were in the Lower Middle Missouri and Lower Missouri River subbasins (>800 kg/km^2^/year and >140 kg/km^2^/year for TP), where much of the agricultural activity (farm fertilizer and manure input) and developed land area in the basin are concentrated (Figure 7b). Farm fertilizer was the largest contributor to the TN yields in six of the seven subbasins, composing 32 to 59% of the total yield (see source shares by major subbasin in Appendix S1). The exception was the Yellowstone River subbasin, where manure was the largest contributor to the TN yield (38%). The source contributions were more variable for TP yields – stream channel sources predominated in the Upper Missouri River and Yellowstone River subbasins (63 and 70%, respectively); point sources predominated in the Platte River subbasin (39%); farm fertilizer predominated in the Middle Missouri, Lower Middle Missouri, and Kansas River subbasins (31 to 44%); and manure and farm fertilizer were equally predominant in the Lower Missouri River subbasin (both 32%). Developed land areas or point sources were the smallest contributor to both TN and TP outlet yields in six of seven major subbasins (ranging from 1 to 9% for nitrogen and 1 to 13% for phosphorus); the exception was in the Platte subbasin, where atmospheric deposition was the smallest contributor to nitrogen yields (7%). Although not directly comparable, estimates of TN and TP yields delivered from states in the Missouri River Basin to the Gulf of Mexico (Alexander *et al.*, 2008) were slightly lower, which may be due in part to additional attenuation between the confluence of the Missouri and Mississippi Rivers and the Gulf of Mexico.

### Total Catchments: Loads Delivered to the Mississippi River From the Major Subbasins

Loads of TN and TP delivered to the Mississippi River varied considerably among the total catchments of the major subbasins. Only 22% of the TN load (2% of TP load) in the Upper Missouri and Yellowstone River subbasins was delivered to the Mississippi River, whereas approximately 100% of the TN and TP loads in the remaining five subbasins were delivered to the Mississippi River (Table 3). The total load delivered to the Mississippi River from the Missouri River Basin was approximately 306 × 10^6^ kg/year for TN and 39 × 10^6^ kg/year for TP (Table 3). Of these loads, the largest percentage was contributed by the Middle Missouri and Lower Missouri River subbasins (28.4 and 29.1% for TN, respectively; 18.6 and 38.7% for TP, respectively), whereas the smallest percentage was contributed by the Upper Missouri and Yellowstone subbasins (0.2 and 0.4% for TN, respectively; 0.1% each for TP) (Table 3). These findings are similar to those reported by Alexander *et al.* (2008) that indicated that the proximity of nutrient sources to large rivers is an important consideration in managing nutrient delivery downstream and to the Gulf of Mexico.

**TABLE 3:** Summary of Loads and Loads Delivered to the Mississippi River From the Total Catchments of the Major Subbasins in the Missouri River Basin

Major Subbasin	Load (10^6^ kg/year) (90% prediction interval)	Load Delivered to Mississippi River (% of load)	Load Delivered to Mississippi River (10^6^ kg/year) (90% prediction interval)	Contribution to the Total Load Delivered to Mississippi River (%)
Total nitrogen				
Upper Missouri	3.2 (0.64-7.7)	22	0.71 (0.09-2.0)	0.2
Yellowstone	5.4 (1.1-15)	22	1.2 (0.15-3.1)	0.4
Middle Missouri	87 (16-217)	100	87 (16-217)	28.4
Platte	46 (13-109)	100	46 (13-109)	15.0
Lower Middle Missouri	46 (12-164)	100	46 (12-164)	15.1
Kansas	36 (7.5-104)	100	36 (7.5-104)	11.8
Lower Missouri	89 (19-171)	100	89 (19-171)	29.1
Missouri River Basin	-	-	306 (67-589)	100
Total phosphorus				
Upper Missouri	0.93 (0.12-2.4)	2	0.02 (0.001-0.07)	0.1
Yellowstone	2.4 (0.30-7.9)	2	0.05 (0.003-0.26)	0.1
Middle Missouri	7.2 (0.54-20)	100	7.2 (0.54-20)	18.6
Platte	5.7 (0.51-16)	100	5.7 (0.51-16)	14.8
Lower Middle Missouri	5.5 (0.61-15)	100	5.5 (0.61-15)	14.0
Kansas	5.3 (0.44-16)	100	5.3 (0.44-16)	13.7
Lower Missouri	15 (0.83-53)	100	15 (0.83-53)	38.7
Missouri River Basin	-	-	39 (2.2-132)	100

### Effects of Irrigation on Nutrient Transport

**FIGURE 8:**
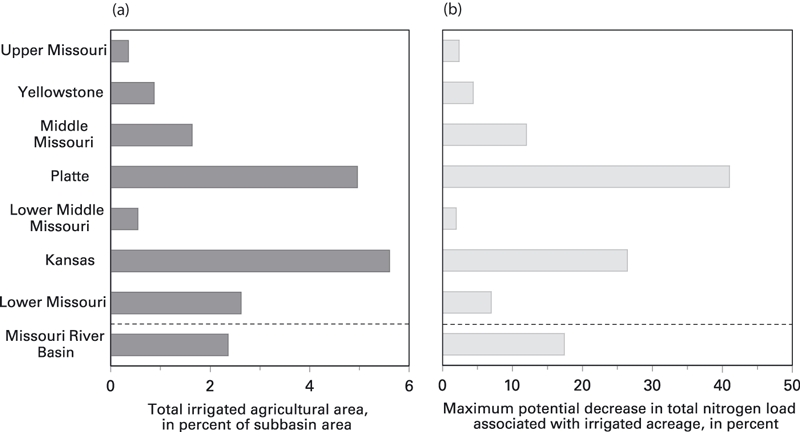
Percentage of (a) Irrigated Acreage on Agricultural Land in the Major Subbasins of the Missouri River Basin and (b) Maximum Potential Decrease in Total Nitrogen Loads From the Major Subbasins of the Missouri River Basin Associated With Irrigated Acreage on Agricultural Land.

Irrigation of agricultural land occurs throughout the Missouri River Basin, but is concentrated in the southern Middle Missouri, eastern Platte, and northern Kansas River subbasins (Figure S16). Large areas of Federal land and other areas with minimal irrigation occur in a large part of the northern and western subbasins. Irrigated area in the subbasins ranged from 0.4 to 6% of the total catchment area, with the Platte and Kansas River subbasins having more than twice the amount of irrigation (5 and 6%, respectively) as most other subbasins (Figure 8a). To estimate the theoretical maximum decreases in the TN loading associated with irrigation in the subbasins, predicted TN loads at recent (1997) levels of irrigated acreage on agricultural land were compared with predicted TN loads from a hypothetical “no irrigated acreage on agricultural land” scenario. The difference in TN load between these scenarios is illustrated in Figure 8b. Because this scenario is simplistic in that it does not consider other probable changes associated with a reduction in irrigated acreage – for example, because some areas of the basin cannot be cultivated without irrigation, the application of fertilizer or manure could change along with irrigation – it is intended to compare the theoretical maximum decrease in TN loading that could be associated with irrigation rather than to estimate the actual decrease in TN loading that is associated with irrigation in each subbasin. Although the resulting estimates may be high relative to the actual net decreases in TN loadings associated with irrigation, they do demonstrate the potentially important effect irrigation has on reducing nitrogen loads in the Missouri River Basin. In the SPARROW model, the quantification of the effects of individual land-water-delivery variables (such as irrigation) is not possible because their coefficients are linked to the source variables; therefore, this alternative scenario approach was used to evaluate the maximum potential effects of irrigation in the Missouri River Basin.

Overall, TN loads delivered to the Mississippi River from the Missouri River Basin were estimated to decrease by as much as 17% with irrigated acreage in the study area. The effect of irrigation on TN loads in the major subbasins was variable (Figure 8b). The largest potential decreases in TN loads were in the Middle Missouri, Platte, and Kansas River subbasins, where the percentage of subbasin area in irrigation was relatively high and TN loads were estimated to decrease as much as 12 to 41% with irrigated acreage. Potential reductions in TN loads associated with irrigated acreage were smaller (between 2 and 7%) in the remaining five subbasins, where the percentage of subbasin area in irrigation was relatively low. Although the percentage of subbasin area in irrigation was similar in the Platte and Kansas River subbasins, the potential reduction in TN loads associated with irrigated acreage was greater in the Platte River subbasin likely because a greater proportion of the total agricultural input (manure and fertilizer) was applied in irrigated areas of the Platte River subbasin (Figures S10, S11, and S16). An additional factor may be the particular spatial distribution of irrigated acreage, fertilizer, and manure inputs within each subbasin relative to other important land-to-water delivery variables. For example, the Lower Middle Missouri and Lower Missouri River subbasins have the highest percentage of loess-dominated surficial geology (46 to 54%), which facilitates nitrogen transport to streams and may be moderating the effect of irrigation (Table S4). Similarly, approximately 31% of the Kansas River subbasin is covered by loess-dominated surficial sediments, whereas only 17% of the Platte River Basin is loess-dominated; this difference may, in part, be contributing to differences in the potential reduction in TN loads associated with irrigated acreage in these two subbasins.

### Effects of Reservoirs and Lakes on Nutrient Transport

Estimated nutrient attenuation in reservoirs and lakes was modeled as a function of apparent settling velocity and areal hydraulic load (Schwarz *et al.*, 2006). This attenuation reflects the net annual balance between processes that supply nutrients (nitrogen fixation, bottom release, and resuspension of phosphorus) and those that remove nutrients (sedimentation, algal uptake, and denitrification) and is strongly related to morphological features of lakes and reservoirs that affect biological and physical processes (Håkanson, 2005; Walker *et al.*, 2007). Sedimentation is acknowledged as the primary mechanism for TP attenuation (Canfield and Bachmann, 1981; Kõiv *et al.*, 2011), whereas denitrification and, to a lesser extent, sedimentation have been shown to be the primary processes responsible for TN attenuation in lakes and reservoirs (Nõges *et al.*, 1998; Saunders and Kalff, 2001). The TN and TP models of the Missouri River Basin included 183 water bodies (two reservoirs in the U.S. part of the Oldman River drainage were included in the model, but excluded from this Missouri River Basin reservoir summary), approximately 80% of which are artificial reservoirs and approximately 20% are natural lakes, some of which may be controlled.

**FIGURE 9:**
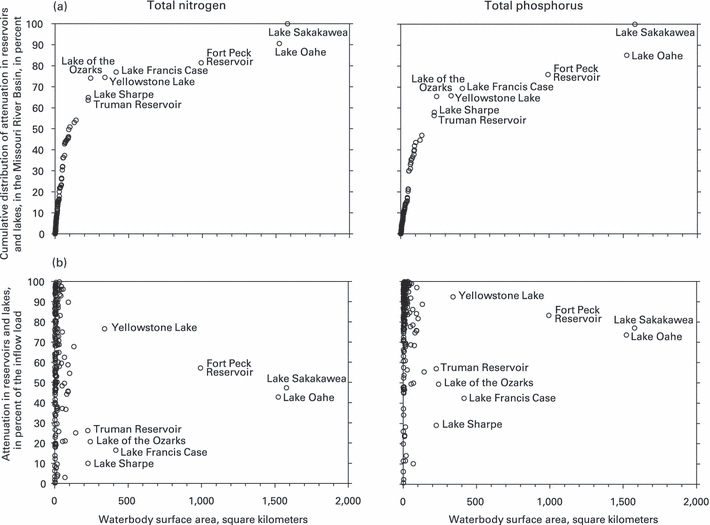
(a) Cumulative Distribution of Attenuation of Total Nitrogen and Total Phosphorus in Reservoirs and Lakes, in Percent (based on a standard cumulative distribution ordered by increasing water-body surface area), and (b) Attenuation of Total Nitrogen and Total Phosphorus in Reservoirs and Lakes, in Percentage of the Inflow Load, in the Missouri River Basin.

Approximately 16% of the TN load and 33% of the TP load that would have otherwise reached the Mississippi River from the Missouri River Basin was retained in reservoirs and lakes. Approximately 54% of the TN attenuation in the basin occurred in water bodies with a surface area <143 km^2^, and the remaining 46% occurred in just eight of the largest (by surface area) water bodies (seven reservoirs and one natural lake) (Figure 9a). Similarly, approximately 47% of the TP attenuation occurred in the water bodies with a surface area <143 km^2^, and the remaining 53% occurred in the same group of seven large reservoirs and one lake (Figure 9a). These eight large water bodies range in size from 225 to 1,578 km^2^ for a total combined surface area of approximately 5,540 km^2^, or 66% of the total reservoir and lake surface area modeled in the Missouri River Basin. Lake Oahe and Lake Sakakawea (Figure 1), the two largest reservoirs, collectively comprise 37% of the total reservoir and lake surface area modeled in the basin and retained approximately 18% of the TN load and 24% of the TP load that would have otherwise traveled downstream to the Mississippi River. These findings agree with those of a previous study that found that attenuation by large reservoir systems can substantially reduce regional nutrient export by rivers (Caraco and Cole, 1999).

Individually, the seven largest reservoirs retained between 10 and 57% of their TN inflow load and between 29 and 77% of their TP inflow load, with TP attenuation consistently exceeding TN attenuation (Figure 9b). Of the eight largest water bodies included in the model, Yellowstone Lake, a large natural lake, had the highest nutrient attenuation at 77% of the inflow TN load and 92% of the TP inflow load. This was likely due to its position in the headwaters of the Yellowstone River subbasin, where inflow discharge, and thus areal hydraulic load, was relatively low compared with that of the other large water bodies. Attenuation of TN and TP in reservoirs and lakes with surface areas approximately <20 km^2^ was highly variable, ranging from 1 to 100% of their inflow nutrient loads (Figure 9b), reflecting the variability in their areal hydraulic loads. Other factors affecting nutrient loss in reservoirs and lakes that are not explicitly modeled in SPARROW, including storage and release operations (reservoirs only), reservoir age, morphology, oxygen status, and temperature, also likely influence the actual attenuation in these reservoirs and lakes (Kimmel and Groeger, 1986; Kennedy and Walker, 1990; Kelly, 2001; Kennedy, 2001; Koszelnik and Tomaszek, 2003; Walker *et al.*, 2007).

**FIGURE 10:**
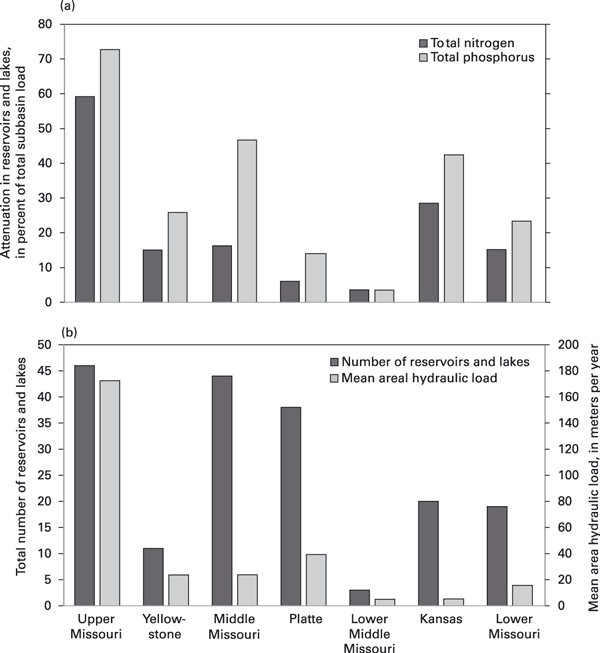
(a) Attenuation of Total Nitrogen and Total Phosphorus in Reservoirs and Lakes and (b) Total Number of Reservoirs and Lakes and Mean Areal Hydraulic Load in the Major Subbasins of the Missouri River Basin.

The percentage of the total subbasin load attenuated in reservoirs and lakes – the total load attenuated in all reservoirs in the subbasin as a percentage of the total load from each subbasin that would have occurred with no loss in reservoirs and lakes – varied among the subbasins (Figure 10a). Note that this estimate of total subbasin load attenuated in water bodies assumes that no additional instream loss would have occurred in the absence of nutrient attenuation in reservoirs and lakes. Subbasin differences in attenuation were related in part to differences in the total number of reservoirs and lakes in each subbasin – with a larger number of water bodies potentially contributing to greater loss of nutrients in reservoirs (Figure 10b); the areal hydraulic load of the reservoirs and lakes in each subbasin – with slowly flushed water bodies with low areal hydraulic loads potentially contributing to greater reservoir attenuation (Figure 10b); and the location of the reservoirs and lakes within each subbasin relative to the largest nutrient inputs – with water bodies downstream from large nutrient inputs potentially contributing to greater reservoir attenuation. It has been well established in the literature that nitrogen and phosphorus retention increases in relation to loading (Jansson *et al.*, 1994; Saunders and Kalff, 2001; Jeppesen *et al.*, 2005; Brett and Benjamin, 2008; Kõiv *et al.*, 2011).

The Upper Missouri River subbasin had the highest TN (59%) and TP (73%) attenuation in water bodies among the subbasins (Figure 10a). The subbasin had the largest number of reservoirs and lakes, but a high mean areal hydraulic load (Figure 10b). More than half of the reservoir attenuation in the subbasin occurred in Lake Fort Peck, a mainstem reservoir that is downstream from the largest nutrient inputs in the subbasin and which has a low mean areal hydraulic load of 7.9 m/year (see SI for explanation of reservoir and lake attenuation calculations).

The Yellowstone River subbasin had lower attenuation for TN (15%) and TP (26%), a lower number of water bodies, and a lower mean areal hydraulic load than the Upper Missouri River subbasin (Figure 10). The low number of reservoirs and lakes, together with the location of the three largest water bodies, including Yellowstone Lake, upstream from most of the nutrient inputs, counteracted the low mean areal hydraulic load in this subbasin.

The Middle Missouri River subbasin, which includes five of the large Missouri River mainstem reservoirs, had relatively low TN attenuation (16%) and moderate TP attenuation (47%) despite having a large number of water bodies and a relatively low mean areal hydraulic load (Figure 10). The four largest mainstem reservoirs in this subbasin (Lake Sakakawea, Lake Oahe, Lake Sharpe, and Lake Francis Case (Figure 1) collectively account for 76% (TN) and 88% (TP) of the attenuation in this subbasin; however, they are upstream from the highest nutrient inputs that occur in the southeastern part of the subbasin.

The Platte River subbasin had low attenuation (6% for TN and 14% for TP) compared with most of the other subbasins, despite having a large number of water bodies and a somewhat low mean areal hydraulic load (Figure 10). Most of the reservoirs and lakes in this subbasin are in the North and South Platte drainages, upstream from the major urban (including developed land and point sources) and agricultural inputs. This geographic distribution of reservoirs and lakes contributes to the relatively high delivery of incremental nutrient loads from the Denver area to the Mississippi River (Figures 4b and 5b).

The Lower Middle Missouri River subbasin, which has just three water bodies in the MRB_E2RF1 reach network used in the models, had the lowest attenuation among all the subbasins (3.6% for TN and 3.5% for TP) (Figure 10). The mean areal hydraulic load was relatively low; consequently, the low number and the location of the reservoirs and lakes in this subbasin were likely important contributors to the low attenuation.

The Kansas River subbasin had moderate attenuation of TN (28%) and TP (42%), a moderate number of water bodies, and a low mean areal hydraulic load compared with the other subbasins (Figure 10). Attenuation in the Kansas River subbasin may have been higher than in other subbasins with a comparable number of water bodies and mean areal hydraulic load due to the attenuation from the five largest (>50 km^2^) water bodies that collectively accounted for 74% of the attenuation in the subbasin. Three of these five reservoirs, which had relatively high areal hydraulic loads, are in the eastern part of the subbasin and in areas of higher nutrient inputs.

The Lower Missouri River subbasin had relatively low attenuation of 15% for TN and 23% for TP, a moderate number of water bodies, and a low mean areal hydraulic load (Figure 10). However, two large reservoirs with high nutrient inputs on the Osage River (Truman Reservoir and Lake of the Ozarks; Figure 1) that collectively resulted in 68% (TN) and 72% (TP) of the total subbasin attenuation had relatively high areal hydraulic loads. Although the nutrient input is relatively high throughout this subbasin, much of the nutrient input was downstream from these reservoirs and in other areas of the subbasin that drain to the Missouri River.

These results indicate that areal hydraulic load was an important determinant of individual and cumulative subbasin attenuation in reservoirs and lakes, and that the total number of water bodies and their location relative to major urban (including developed land and point sources) and agricultural inputs also played a role.

No distinction between reservoirs and lakes was made in the models because data sufficient to distinguish them were not available. Studies evaluating nutrient retention in reservoirs and lakes by Bachmann (1980), Canfield and Bachmann (1981), Kimmel and Groeger (1984), and Kennedy (2001) have discussed similarities between water-body types. Bachmann (1980) and Canfield and Bachmann (1981) reported comparable results when separate or combined equations were used when testing a large dataset of natural and artificial lakes in TN and TP volumetric loading models. Harrison *et al.* (2009) showed that the amount of nitrogen removed by large lakes and large reservoirs is globally similar (3.7 × 10^9^ and 3.6 × 10^9^ kg N/year, respectively), although on a per-unit basis, large and small reservoirs retained a much larger amount of nitrogen (24,000 to 30,612 kg N km^2^/year) than large and small lakes (3,083 to 3,577 kg N km^2^/year). Differences in reservoir and lake characteristics, such as hydraulic residence time (typically longer in natural lakes than reservoirs) and ratio of drainage area to surface area (typically smaller in natural lakes than reservoirs), can affect nutrient retention, as identified in studies by Kennedy and Walker (1990), Straškraba *et al.* (1995), Kennedy (2001), Hejzlar *et al.* (2006), Walker *et al.* (2007), and Harrison *et al.* (2009). Additionally, characteristics of individual reservoirs are highly variable, with some resembling rivers (run-of-the-river reservoirs like those on the mainstem of the Missouri River), and others more closely resembling lakes (tributary-storage reservoirs). Run-of-the-river reservoirs are considered semi-fluvial environments and are typically characterized by extensive longitudinal gradients, shorter hydraulic residence times, and more turbidity (Kimmel and Groeger, 1984; Kennedy, 2001), although variability in morphological and internal and upstream hydraulic characteristics among these water bodies can greatly affect the downstream transport of nutrients (Kelly, 2001). Additional factors not explicitly considered in the models, such as reservoir morphology and operational characteristics, could provide further insight into differences in nutrient attenuation within the Missouri River Basin.

## Summary and Conclusions

Spatially referenced regression (SPARROW) modeling was used to identify the major nutrient sources and terrestrial and aquatic delivery factors influencing nitrogen and phosphorus loads in the Missouri River Basin and to evaluate the effects of reservoir storage and irrigation on those loads. Farm fertilizer was the largest contributor to the nitrogen yields in six of the seven major subbasins. Source contributions were more variable for phosphorus: stream channel sources predominated in the Upper Missouri River and Yellowstone River subbasins; point sources predominated in the Platte River subbasin; farm fertilizer predominated in the Middle Missouri, Lower Middle Missouri, and Kansas River subbasins; and manure and farm fertilizer were equally predominant in the Lower Missouri River subbasin. Developed land area and point sources were the smallest contributors to both nitrogen and phosphorus loads in six of seven subbasins. Nitrogen delivery to streams was affected by precipitation, air temperature, percentage of irrigated acreage, and percentage of loess in surficial sediments; phosphorus delivery to streams was affected by precipitation, soil permeability, and mean basin slope. Additionally, nitrogen was attenuated in small streams and in reservoirs and lakes; phosphorus was attenuated in reservoirs and lakes, but not in streams. The inclusion of reach length as a source term in the phosphorus model indicated that the stream channels of medium and larger streams were a net source of phosphorus. Because of a combination of high agricultural and urban inputs and proximity to the Mississippi River, the Middle Missouri and Lower Missouri River subbasins contributed the largest percentage of the nutrient load reaching the Mississippi River. The Upper Missouri and Yellowstone subbasins, with lower inputs, one or more large reservoirs, and greater travel time to the Mississippi River, contributed the smallest percentage.

Nutrient loads were found to decrease as a result of both irrigation and reservoir storage. Nitrogen loads from the Missouri River Basin to the Mississippi River were estimated to decrease by as much as 17% with irrigated acreage on agricultural land, likely due to increased anoxia and denitrification in the soil zone. The largest potential decreases with irrigated acreage were in the Middle Missouri, Platte, and Kansas River subbasins, where the percentage of subbasin area in irrigation was relatively high. Nutrient loads from the Missouri River Basin to the Mississippi River also decreased by approximately 16% for nitrogen and 33% for phosphorus as a result of attenuation in reservoirs and lakes. Approximately half of that attenuation occurred in just eight of the largest (by surface area) water bodies (seven reservoirs and one natural lake). Attenuation of nutrients in reservoirs and lakes within the subbasins varied with the total number of water bodies in the subbasin, their areal hydraulic loads, and their locations relative to the largest nutrient inputs. Unlike the other major tributary basins, nearly the entire instream nutrient load leaving the outlet of the Platte and Kansas River subbasins reached the Mississippi River. The Platte River subbasin had low attenuation compared with that in most of the other subbasins, despite having a large number of water bodies. Most of these water bodies, however, are in the North and South Platte drainages, upstream from the major urban (including developed land and point sources) and agricultural inputs in the subbasin. In the Kansas River subbasin, most of the source inputs are in the southeast part of the subbasin where characteristics of the area and proximity to the Missouri River facilitate the delivery of nutrients to the Mississippi River.

The results from the Missouri River Basin TN and TP models suggest some unique findings when compared with previously published SPARROW models and indicate that some aspects of nutrient cycling in the Missouri River Basin may be atypical of other areas of the U.S. Unlike findings in previous studies, streams in the basin did not appear to be net sinks for phosphorus; indeed, stream channels of medium and larger streams were found to be a significant source for phosphorus and indicate that channel erosion and scouring may not be in long-term equilibrium with deposition in the Missouri River Basin. Estimates of the fraction of nonpoint-source inputs of TN and TP delivered to streams were low compared with those from other national and regional SPARROW models. This is likely related to the significantly lower runoff rate in the basin when compared with other parts of the conterminous U.S. The identification of irrigated agricultural land as a significant negative land-to-water delivery variable (TN model) has not been previously published and suggests that further research into the regional effects of irrigation on nutrient transport is warranted; it also points to the need for enhanced regional or national irrigation datasets. The substantial nutrient attenuation identified in the large mainstem reservoirs has not previously been reported and indicates the importance of considering these water-body features in regional nutrient management planning.

Insights into nutrient sources and nutrient transport in the Missouri River Basin could be advanced by verification of point-source locations and loads and the inclusion of additional monitoring station data and regional datasets. Water transfers by diversions, canals, and pipelines in the Missouri River Basin are not well represented in the reach network due to their overall complexity and the lack of information – inclusion of these data would contribute to more accurate load and yield estimates in areas greatly affected by diversions. Nutrient transport related to aspects of irrigation that can be managed could be evaluated if regional data on volume of irrigated water applied or the type of irrigation used were available. Distinctions between reservoirs and lakes and delineations based on morphological or reservoir operational factors may aid in further understanding and quantifying nutrient transport and aquatic-loss occurring in these water bodies. Use of a higher-resolution stream network could provide refined estimates for source delineations, land-to-water delivery processes, and stream reach and water-body nutrient transport and attenuation. The scale of the geospatial data used to calibrate the models, the geospatial distribution of the calibration sites, as well as the increased uncertainty of predictions at locations and scales that differ from those of the monitoring data used for model calibration should be considered when deciding at what scale to use these results to inform management strategies.

The results of this study suggest that irrigation and reservoirs could play a role in regional nutrient management in the Missouri River Basin. The effects of any changes in irrigated acreage or reservoir storage (or any source or delivery factor), however, would need to be considered together with the effects of changes in other sources or delivery factors that might occur concurrently; for example, fertilizer application might also increase if irrigated acreage were to increase. Although this paper highlights the effects of irrigation and reservoirs on nutrient loads, the Missouri River Basin SPARROW models could be helpful in addressing a wide range of management issues. Understanding the sources and processes influencing nutrient transport in the Missouri River Basin has local, regional, and national implications for nutrient management efforts. For example, sources contributing the most to instream loads throughout the Missouri River Basin could be targeted for nutrient reduction, including the development of total maximum daily loads. Stream reaches with high nutrient loads could be prioritized for local nutrient-reduction strategies, whereas stream reaches contributing high loads to the Mississippi River, such as those in close proximity to the Mississippi River and downstream from large reservoirs, could be prioritized as part of regional or national nutrient-reduction efforts. Moreover, modeling results could aid in identification of data gaps and prioritization of locations for future monitoring in order to optimize limited resources available for monitoring.

## SUPPORTING INFORMATION

**Document S1.** Additional supporting information mentioned in the text, including basin description, additional SPARROW model details, calculation of reservoir and lake attenuation values, catchment source shares, and an overview of input data used in the TN and TP Missouri River Basin SPARROW models, may be found in the online version of this article. Additionally, a web-based decision support system (DSS) has been developed to provide access to SPARROW model data, including input data, load estimates, confidence intervals, and standard errors of prediction. The DSS also offers scenario testing of altered inputs for research and water-quality planning (Booth et al., 2011; this issue). The SPARROW DSS application is available at: http://water.usgs.gov/ nawqa/sparrow/dss. [Correction made here after initial online publication..

**Figure S1.** Geography of the Missouri River Basin.

**Figure S2.** (a) Land use and (b) physiography of the Missouri River Basin.

**Figure S3.** Model diagnostics for total nitrogen: (a) observed against predicted load; (b) observed against predicted yield; (c) log residuals against predicted load; and (d) log residuals against predicted yield.

**Figure S4.** Model diagnostics for total phosphorus: (a) observed against predicted load; (b) observed against predicted yield; (c) log residuals against predicted load; and (d) log residuals against predicted yield.

**Figure S5.** Mean annual load estimated from stream water-quality monitoring data and discharge measurements for (a) total nitrogen and (b) total phosphorus, in kilograms per year, in the Missouri River Basin, normalized to 2002.

**Figure S6a.** Boxplots of basin characteristics for sources used in the TN and TP SPARROW models for the calibration reaches and all the reaches in the Missouri River Basin.

**Figure S6b.** Boxplots of basin characteristics for land-to-water delivery variables used in the TN and TP SPARROW models for the calibration reaches and all the reaches in the Missouri River Basin. [Correction made here after initial online publication.]

**Figure S7.** Estimates of nutrient contributions from point sources based on the mean of 1992, 1997, and 2002 data for (a) total nitrogen and (b) total phosphorus, in kilograms, in the Missouri River Basin.

**Figure S8.** Developed land (all classes) normalized by incremental catchment area, in percent, in the Missouri River Basin.

**Figure S9.** Open-space developed land (a) as a percent of total developed land area, and (b) normalized by incremental catchment area, in percent, in the Missouri River Basin, 2001.

**Figure S10.** Nutrient inputs per unit area from farm fertilizer as (a) total nitrogen and (b) total phosphorus, by county in kilograms per square kilometer, in the Missouri River Basin, 2002.

**Figure S11.** Nutrient inputs per unit area from confined and unconfined manure as (a) total nitrogen and (b) total phosphorus, by county in kilograms per square kilometer, in the Missouri River Basin, 2002.

**Figure S12.** Nitrogen inputs from atmospheric deposition from measurements of wet deposition of total inorganic nitrogen, in kilograms per square kilometer per year, in the Missouri River Basin, 2002.

**Figure S13.** Stream channel length as a source of phosphorus (for streams where discharge is >1.13 m^3^/s), in kilometers, in the Missouri River Basin.

**Figure S14.** Mean annual precipitation, in millimeters, in the Missouri River Basin, 1971-2000.

**Figure S15.** Mean of maximum and minimum air temperature, in degrees Celsius, in the Missouri River Basin, 1971-2000.

**Figure S16.** Estimated agricultural area in gravity and pressure irrigation, in percent, in the Missouri River Basin, 1997.

**Figure S17.** Loess-dominated surficial geology in the Missouri River Basin.

**Figure S18.** Mean soil permeability, in centimeters per hour, in the Missouri River Basin.

**Figure S19.** Mean basin slope, in percent, in the Missouri River Basin.

**Table S1.** Sources of water-quality and discharge monitoring data used to estimate mean annual nutrient load.

**Table S2.** List of Standard Industrial Classification codes included and excluded from the Missouri River Basin SPARROW models.

**Table S3.** National Land Cover Database class definitions for developed land, 2001 (from http://www. mrlc.gov/nlcd_definitions.php, accessed June 2011).

**Table S4.** Summary of loess-dominated surficial geology by major subbasin.

**Appendix S1.** Source shares (in percent of load) in the total catchment of the major subbasins. Source shares correspond to the data in Figure 7a in main manuscript. [Correction made here after initial online publication.]

Please note: Neither AWRA nor Wiley-Blackwell is responsible for the content or functionality of any supporting materials supplied by the authors. Any queries (other than missing material) should be directed to the corresponding author for the article.
