# Supplementary material for: Nutrient Sources and Transport in the Missouri River Basin, with Emphasis on the Effects of Irrigation and Reservoirs
Source: J Am Water Resour Assoc. 2011 Aug 22;47(5):1034–60. doi: 10.1111/j.1752-1688.2011.00584.x (PMC3307633; doi:10.1111/j.1752-1688.2011.00584.x)
Supplement: Supplementary file 1 [file jawr0047-1034-SD1.pdf]

Nutrient sources and transport in the Missouri River Basin, with emphasis on the effects of reservoirs and irrigation

Juliane B. Brown, Lori A. Sprague, and Jean A. Dupree

Supporting Information

Basin Description

The Missouri River drains more than 1,371,000 km^2, one-sixth of the conterminous United States, and flows 3,767 km from its headwaters in the Rocky Mountains through the Great Plains to its confluence with the Mississippi River (Sprague *et al.*, 2006). In 2000 approximately 11 million people lived in the basin, with urban areas concentrated near large cities like Denver, Colorado; Omaha, Nebraska; and Kansas City, Missouri (Figure S1; Sprague *et al.*, 2006). Land use in the Missouri River Basin is primarily grassland and shrubland (typically used for range) in the northwestern and central part of the basin and primarily cultivated (including pasture and row crops) in the southeastern half of the basin (Figure S2a). The basin has a strong east-west topographic and climatic gradient, reflected in the basin's seven major physiographic provinces — the steep mountains, high plateaus, and intervening valleys of the Northern, Southern, and Middle Rocky Mountain Provinces and the Wyoming Basin Province; the semiarid elevated tablelands and smooth grasslands of the Great Plains Province; the gently rolling hills and intermittent escarpments of the Central Lowland Province; and the deep stream valleys and intervening ridges in the Ozark Plateaus Province (Fenneman and Johnson, 1946) (Figure S2b). Climatic conditions range from cold and moist in the Rocky Mountains to semiarid in the Great Plains to humid continental in the Central Lowlands.

Figure S1. Geography of the Missouri River Basin.

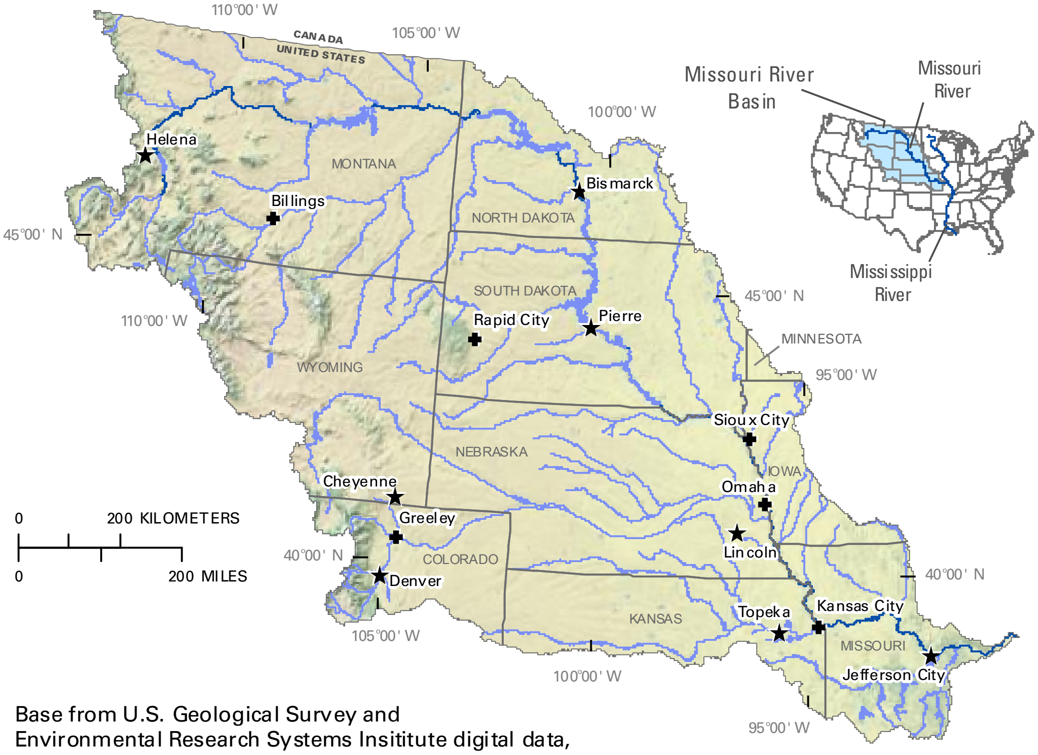

Base from U.S. Geological Survey and
Environmental Research Systems Insititute digital data,
http://resources.esri.com/arcgisdesktop/layers

Figure S2. (a) Land use and (b) physiography of the Missouri River Basin.

(a) Land use

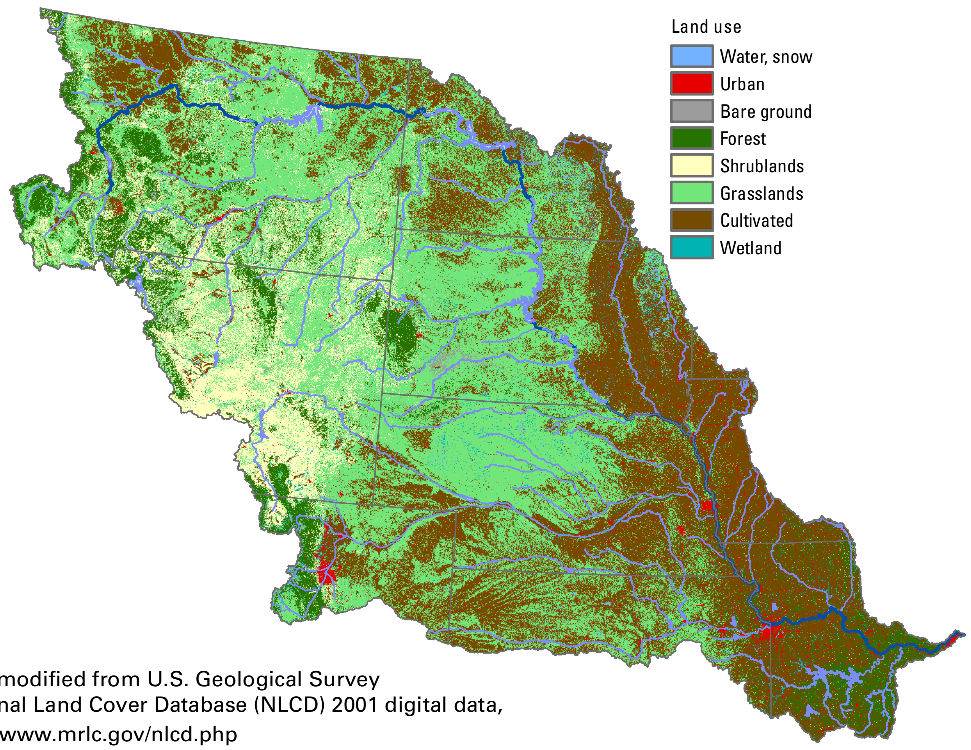

Base modified from U.S. Geological Survey
National Land Cover Database (NLCD) 2001 digital data,
http://www.mrlc.gov/nlcd.php

(b) Physiography

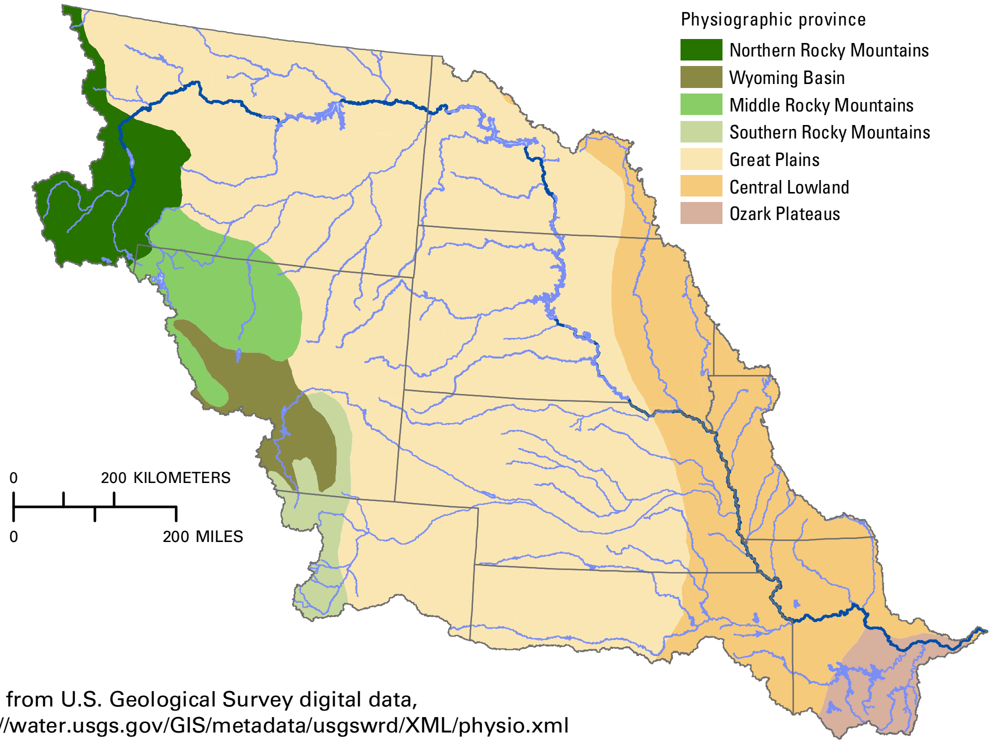

Base from U.S. Geological Survey digital data,
http://water.usgs.gov/GIS/metadata/usgswrd/XML/physio.xml

Additional SPARROW model details

The mathematical form of the SPARROW models is that of a nonlinear regression model in which nutrient loads are related to nutrient-source data weighted by estimates of loss due to land-surface and instream processes (Smith *et al.*, 1997). As described in Alexander *et al.* (2008) and detailed in Schwarz *et al.* (2006), the mean annual load in leaving reach *i* is given by

$$F_i^* = \left[(\sum_{j \in J(i)} F_j^{'}) A(Z_i^S, Z_i^R; \theta_S, \theta_R) + (\sum_{n-1}^{Ns} S_{n,i} \alpha_n D_n (Z_i^D; \theta_D)) A^{'}(Z_i^S Z_i^R; \theta_S, \theta_R) \right] \varepsilon_i . \quad \text{(S1)}$$

The first summation term in the above equation (S1) represents the total load delivered to reach *i* from upstream reaches, where F_j*'* is the measured load if the upstream reach is monitored or the model-estimated load if it is not. The *A(·)* term represents any stream delivery factors that cause load to be lost as it travels along the reach. Within this term, the $\boldsymbol{Z}^S$ and $\boldsymbol{Z}^R$ vectors (with corresponding coefficient vectors θ_S and θ_R) represent losses in measured stream and reservoirs, respectively. If reach *i* is a stream, then only the $\boldsymbol{Z}^S$ and θ_S terms determine the value of *A(·)*; conversely, if reach *i* is a reservoir then $\boldsymbol{Z}^R$ and θ_R determine *A(·)*. The second summation term represents the amount of the within-reach load introduced to stream reach *i*. This term is composed of load originating in individual modeled sources, each source being indexed by $n=1,...N_S$. Each source has a source variable, S_n , and a source coefficient, α_n, that measures the intensity of source contribution. The function $D_n(\cdot)$ represents land-to-water delivery factors, and, coupled with the coefficient, represents the rate at which the source variable is converted to nutrient mass delivered to streams. The land-to-water delivery factor is a source-specific function of a vector of delivery variables, represented by $\boldsymbol{Z}_i^D$ in the equation (S1), and an associated vector of coefficients θ_D. The last term in the equation, the function *A'(·)*, represents the fraction of the load originating in and delivered to reach *i* that is transported to the reach's downstream node. If reach *i* is a stream reach (as opposed to a reservoir reach), the nitrogen load or phosphorus load introduced to reach *i* from the incremental drainage for reach *i* is attenuated to receive the square root of the reach's full instream delivery. For reservoir reaches, the assumption is made that the nutrient mass receives the full attenuation, which is tabulated as a reach attribute. The multiplicative error term in equation (S1), ε_i, is applicable in cases where reach *i* is a monitored reach; the error is assumed to be independent and identically distributed across independent catchments in the intervening drainage between stream monitoring sites.

The measured load in monitored reaches for the 2002 base year are estimated by detrending the data at each site. According to Schwarz *et al.* (2006, p. 20), the detrended time series of load at a site can be interpreted as the series that would have been observed if the dynamic factors causing trend over time, whatever they might be, were held constant throughout the entire period of record, equal to the values they had in 2002 (the base date). All other dynamic factors determining the short-term variations in the series are left unchanged. Therefore, for example, peak-flow events affecting the original series would remain in the detrended series; however, gradual improvements in water quality resulting from the implementation of

management practices over time would be substituted with management practices that were in place in 2002. The mean of the detrended series was used to represent the load that would have occurred during 2002 under long-term mean hydrologic conditions. In mathematical terms, the detrending process can be described as follows. Let $h(t)$ be the function of time used to describe trend through the original series $X(t)$. The detrended series $X^*(t)$ is given by

$$X^*(t) = X(t) - (h(t) - h(T_o)) \qquad \text{(S2)}$$

In equation (S2), the term $h(t) - h(T_o)$ is the adjustment function and the constant $h(T_o)$ is the constant that causes the adjustment to equal zero for the base date, T_o.

Two notable specifications affecting model calibration and prediction were applied to the Missouri River Basin SPARROW model. To preserve the overall mass balance of the model, predicted loads were not conditioned on measured load at monitored reaches (if_adjust = no); therefore, the predictions are based only on the estimated SPARROW model. To aid in interpretation of the model output by providing standardized delivery and source coefficients, the land-to-water delivery variables were expressed as differences from their mean value over all reaches (if_mean_adjust_delivery_vars = yes) (Schwarz *et al.*, 2006).

The mean discharge and travel time attributes in the reach network used for the Missouri River Basin SPARROW model (MRB_E2RF1) were refined using an alternative measure of mean discharge that was based on an interpolation of USGS streamgage estimates from 1975 to 2007, with extrapolation of discharge upstream from gages based on runoff estimated at downstream or neighboring stations and apportioned to the land surface according to the MRB_E2RF1 catchments (Brakebill *et al.*, this issue.). The existing reservoir coverage in the MRB_E2RF1 reach network was modified to include nine additional reservoirs with maximum capacity greater than 61,674,093 m^3 (50,000 acre feet) or normal capacity greater than 12,334,819 m^3 (10,000 acre-feet) using the National Inventory of Dams (U.S. Army Corps of Engineers, 2005), a USGS major dams of the United States map layer (http://www.nationalatlas.gov/mld/dams00x.html, March 2006 release, *accessed* June 2011), waterbodies from the National Hydrography Dataset (http://nhd.usgs.gov/, *accessed* June 2011), and digital topographic maps (http://resources.esri.com/arcgisdesktop/layers, *accessed* June 2011), for a total of 183 reservoirs and lakes in the Missouri River Basin (two reservoirs in the U.S. part of the Oldman River drainage were included in the model, but excluded from the Missouri River Basin reservoir summary).

The spatial data on nutrient inputs and landscape characteristics were limited to the basin area in the United States; the basin area in Canada initially was undefined. Nutrient inputs and landscape characteristics for 93 reaches with catchments in Canada were estimated as the mean value of all reaches in the United States in the same 8-digit hydrologic unit code. Because of the irregular location of point sources throughout the hydrologic unit, the mean was not used to estimate contributions of nutrients from point sources in the Canadian reaches; instead, the point-source contributions were treated as missing. The values estimated for reaches in Canada were used in calibration and prediction to maintain water flow paths and mass balance throughout the Missouri River Basin reach network. But because the uncertainty in these values is expected to be greater than the uncertainty in values for reaches in the United States, predictions for reaches in Canada are not reported here.

As stated in the main article, plots of the observed loads versus predicted loads and observed yields versus predicted yields from the Missouri River Basin TN and TP SPARROW models suggest reasonably unbiased models, though values are more variable for sites with small loads and generally coverage is sparse for sites with very small and very large loads (Figures S3 and S4 [a and b]). Slightly larger variation is evident for total phosphorus as compared with total nitrogen, which is consistent with the higher RMSE of the TP model. Plots of the prediction loads and yields versus residuals indicate that the residuals were approximately homoscedastic (Figures S3 and S4 [c and d]).

Figure S3. Model diagnostics for total nitrogen: (a) observed against predicted load; (b) observed against predicted yield; (c) log residuals against predicted load; and (d) log residuals against predicted yield. [One-to-one line shown on graphs.]

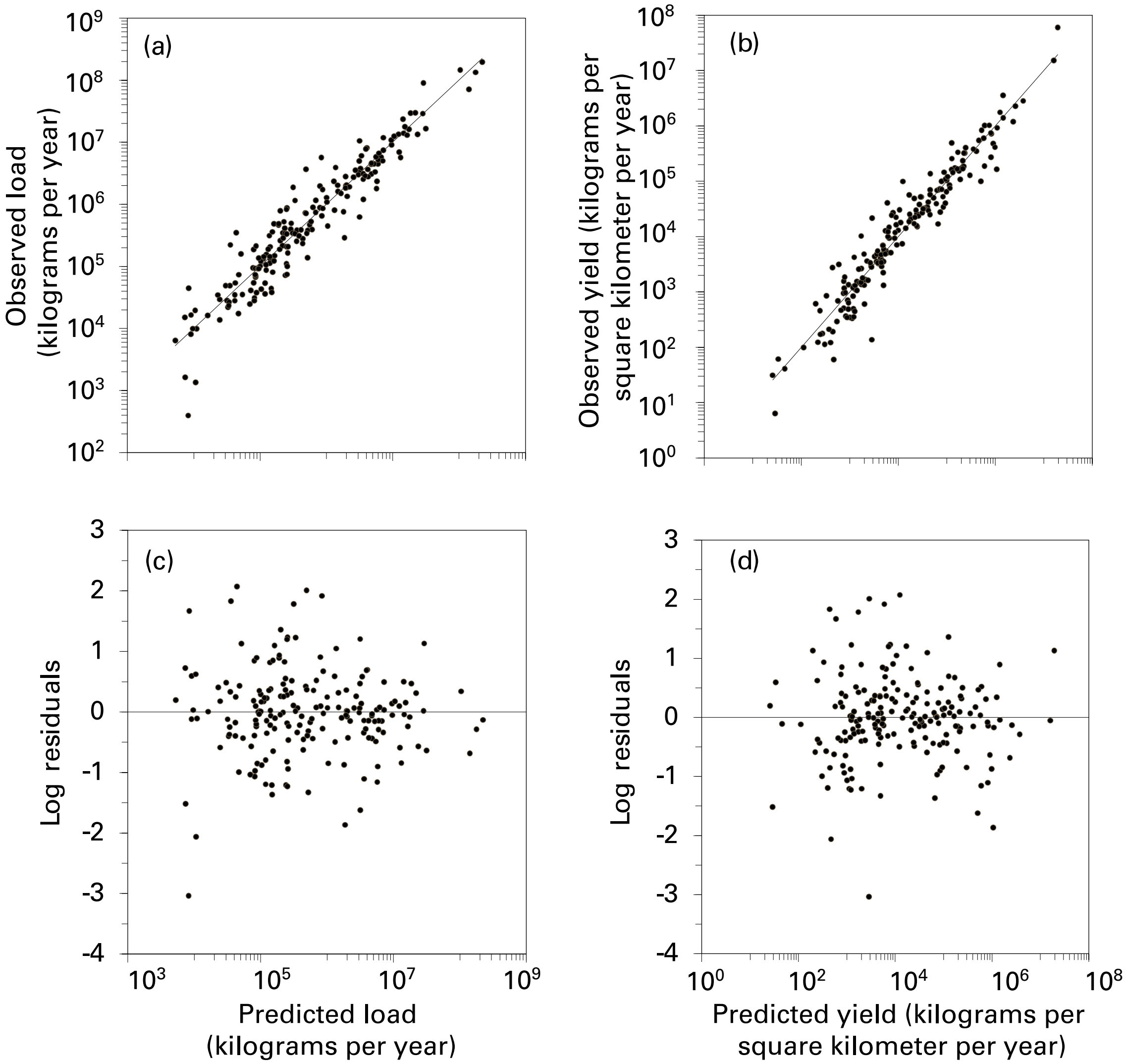

Figure S4. Model diagnostics for total phosphorus: (a) observed against predicted load; (b) observed against predicted yield; (c) log residuals against predicted load; and (d) log residuals against predicted yield. [One-to-one line shown on graphs.]

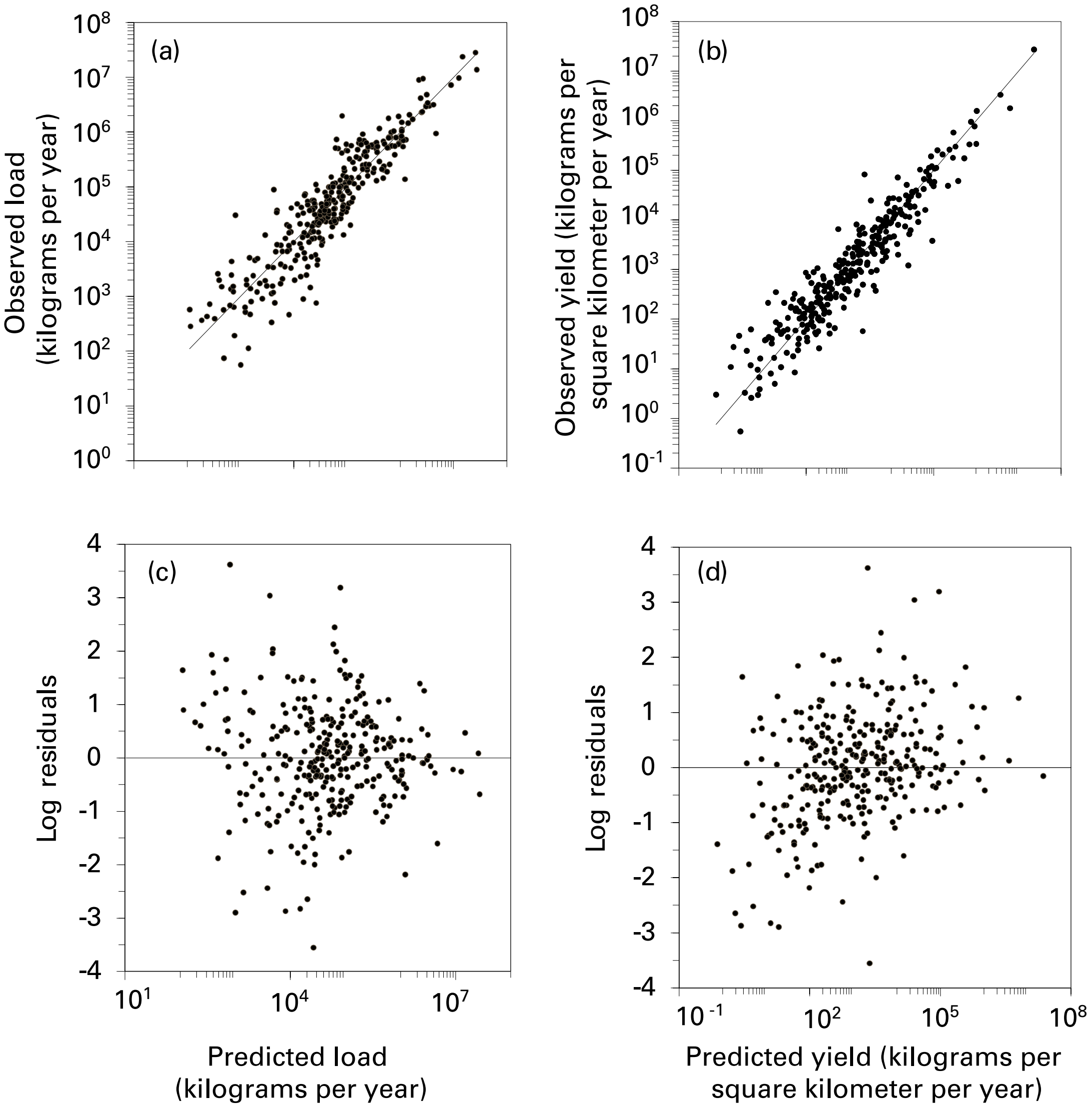

Calculation of reservoir and lake attenuation values

Estimates of attenuation in individual reservoirs and lakes were computed by dividing the total load attenuated in each waterbody by the total load entering that waterbody. Because the total load entering each waterbody was not available directly from the SPARROW model output, it was calculated as the sum of the total load attenuated in the waterbody and the total load leaving the reach. Overall, this calculation is given by:

$$RESERVOIR\ ATTENUATION = (RES\_DECAY / (PLOAD\_TOTAL + RES\_DECAY)) * 100 \quad \text{(S3)}$$

where

RESERVOIR ATTENUATION = attenuation in the individual reservoir or lake, in percent of the total load entering the waterbody;

RES_DECAY = the load attenuated in the individual reservoir or lake, in kg/yr; and

PLOAD_TOTAL = the total load leaving the reach (i.e., the reach entering the reservoir), in kg/yr.

The *RES_DECAY* and *PLOAD_TOTAL* variables, provided as part of the SPARROW model output, are further explained in Schwarz *et al.* (2006). Individual error on these attenuation estimates was not computed.

Estimates of attenuation in the reservoirs and lakes within each major subbasin were computed by dividing the total load attenuated in all reservoirs and lakes in each subbasin by the total load that would have left that subbasin had there been no loss in any of the reservoirs and lakes. This calculation is given by:

$$SUBBASIN\ ATTENUATION_S = \left(\frac{\sum_{r=1}^{n} RES\_DECAY_r}{PLOAD\_ND\_TOTAL_S} \right) *100 \quad \text{(S4)}$$

where

$SUBBASIN\ ATTENUATION_s$ = attenuation in the *n* reservoirs and lakes in subbasin *s,* in percent of the total subbasin load;

RES_DECAY_r = the load attenuated in reservoir or lake *r* in subbasin *s*, in kg/yr; and

$PLOAD\_ND\_TOTAL_s$ = the total load leaving the outlet reach of subbasin *s,* excluding instream and reservoir attenuation, in kg/yr.

This approach assumed that no additional instream loss would have occurred in the absence of nutrient attenuation in reservoirs and lakes (G.E. Schwarz, U.S. Geological Survey, oral commun., 2009). Individual error on these attenuation estimates was not computed.

SPARROW input data

Calibration data

The spatial distribution of the TN and TP loads used in model calibration are shown in Figure S5. Information on sources of water-quality data and discharge measurements used to estimate mean annual nutrient load are presented in Table S1. The highest TN loads (greater than 10,000,000 kg/yr) are concentrated in the southeast part of the basin (Figure S5a). Although the highest TP loads (greater than 1,000,000 kg/yr) are similarly concentrated in the southeast, additional higher TP load sites were identified in the central and north-central part of the study area (Figure S5b). More information on the preprocessing of the calibration data is provided in the Methods section of the report and in Saad *et al.* (this issue).

Boxplots of basin characteristics used in the TN and TP SPARROW models for the calibration reaches and all the reaches in the Missouri River Basin (Figure S6 [a and b]) indicate that source and transport attributes of the calibration sites generally corresponded well to the attributes of the Missouri River Basin reaches used during model prediction. The calibration sites generally did not capture the extremes of these basin characteristics. However, the calibration sites on average had somewhat higher developed land area, mean temperature, mean precipitation, irrigated area, and area in loess surficial geology than those throughout the Missouri River Basin (Wieczorek and LaMotte, 2011; Maupin and Ivahnenko, this issue).

Figure S5. Mean annual load estimated from stream water-quality monitoring data and discharge measurements for (a) total nitrogen and (b) total phosphorus, in kilograms per year, in the Missouri River Basin, normalized to 2002.

(a) Total nitrogen

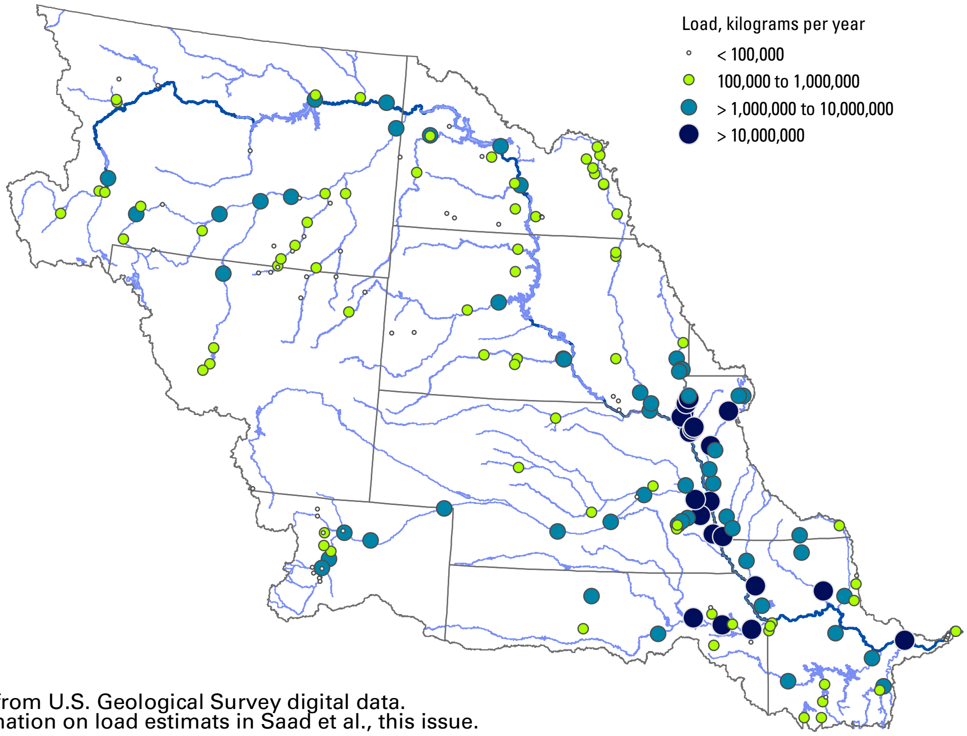

Base from U.S. Geological Survey digital data.
Information on load estimats in Saad et al., this issue.

(b) Total phosphorus

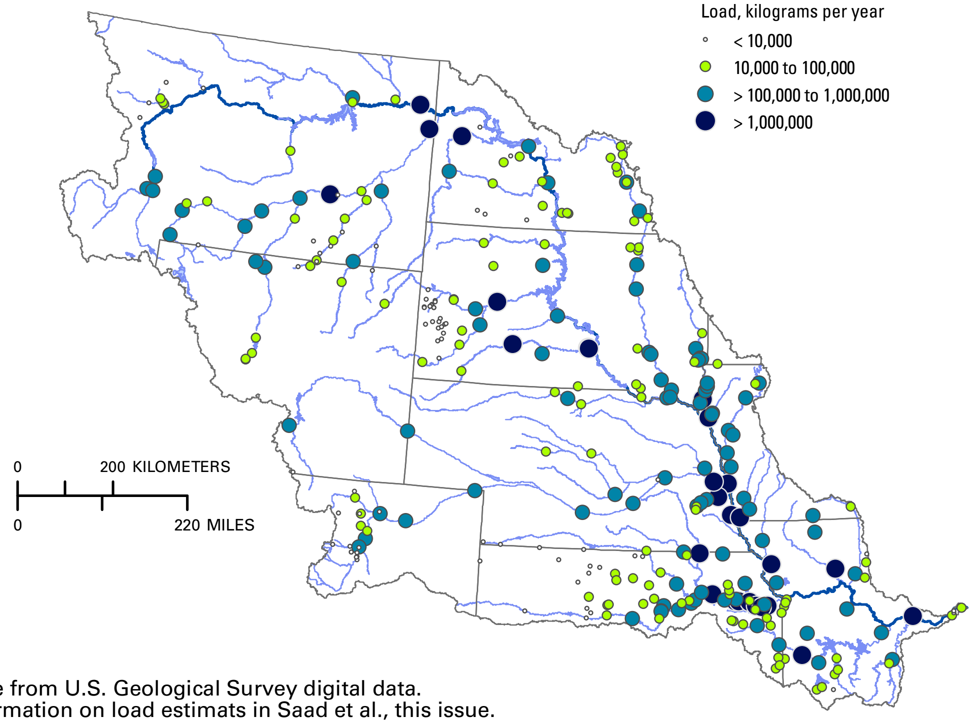

Base from U.S. Geological Survey digital data.
Information on load estimats in Saad et al., this issue.

Table S1. Sources of water-quality and discharge monitoring data used to estimate mean annual nutrient load.

[MODNR, Missouri Department of Natural Resources; NWIS, National Water Information System; sites, water-quality sampling sites; stations, streamflow gaging stations; STORET-LEG, Legacy STORET; STORET-MOD, Modernized STORET; TN, total nitrogen; TP, total phosphorus; All states = CO, IA, KS, MO, MT, ND, NE, SD, WY, no sites in MN; Note: Not all sites used in final models]

Agency type	Monitoring agency	Agency Code	Database	No. of sites TN	No. of sites TP	State(s)
			**Water-quality sites**			
Local	Centennial Water And Sanitation District (Colorado)	CWSD	STORET-MOD	2	2	CO
State	Colorado Department Of Public Health & Environment	21COL001	STORET-MOD	4	5	CO
Federal	U.S. Army Corps Of Engineers	121MBRCE	STORET-LEG	8	11	MO, KS
Federal	U.S. Army Corps Of Engineers, Kansas City District	KCCOE	MODNR	1	1	NE
Federal	U.S. Army Corps Of Engineers, Kansas City District	KCDCOE	MODNR	1	1	MO
State	Iowa Department Of Natural Resources	IDNR	MODNR	1	1	IA
State	Iowa Department Of Environmental Protection	21IOWA	STORET-LEG	5	5	IA
State	Iowa Department Of Natural Resources	21IOWA	STORET-MOD	4	4	IA
State	Kansas Department Of Health & Environment	21KAN001	STORET-LEG	4	14	KS
State	Kansas Department Of Health & Environment	21KAN001	STORET-MOD	3	52	KS, NE
Local	Metro Waste Water Reclamation District (Colorado)	MWRD	STORET-MOD	2	2	CO
State	Minnesota Pollution Control Agency	MNPCA1	STORET-MOD	0	1	IA, MN
State & Federal	Missouri Department Of Natural Resources/U.S. Army Corps Of Engineers	MODNR/KC	MODNR	1	1	MO
Federal	National Park Service	11NPSWRD	STORET-MOD	1	1	CO
State	North Dakota Department Of Health	21NDHDWQ	STORET-MOD	10	13	ND
State	South Dakota Department Of Environmental & Natural Resources	21SDAK01	STORET-LEG	2	3	IA, SD
State	South Dakota Department Of Environmental & Natural Resources	21SDAK01	STORET-MOD	19	59	IA, SD
State	South Dakota Department Of Environmental & Natural Resources	SDWRAP	STORET-MOD	1	1	SD
Federal	U.S. Army Corp Of Engineers (CEMRO-ED-H)	COEOMAHA	STORET-LEG	5	5	CO, ND, SD
Federal	U.S. Environmental Protection Agency	11EPALES	STORET-LEG	1	1	MO
Federal	U.S. Environmental Protection Agency Region 7	1117MBR	STORET-LEG	3	3	IA, MO
Federal	U.S. Geological Survey	USGS	NWIS	135	154	ALL
**Agency type**	**Monitoring agency**	**Agency Code**		**No. of stations**		**State(s)**
			**Streamflow gaging stations**			
Federal	U.S. Geological Survey	USGS	NWIS	281		ALL
State	Colorado Division Of Water Resources	CDWR	na	1		CO
Federal	U.S. Army Corps Of Engineers	USCOE	na	1		SD

Figure S6a. Boxplots of basin characteristics for sources used in the TN and TP SPARROW models for the calibration reaches and all the reaches in the Missouri River Basin.

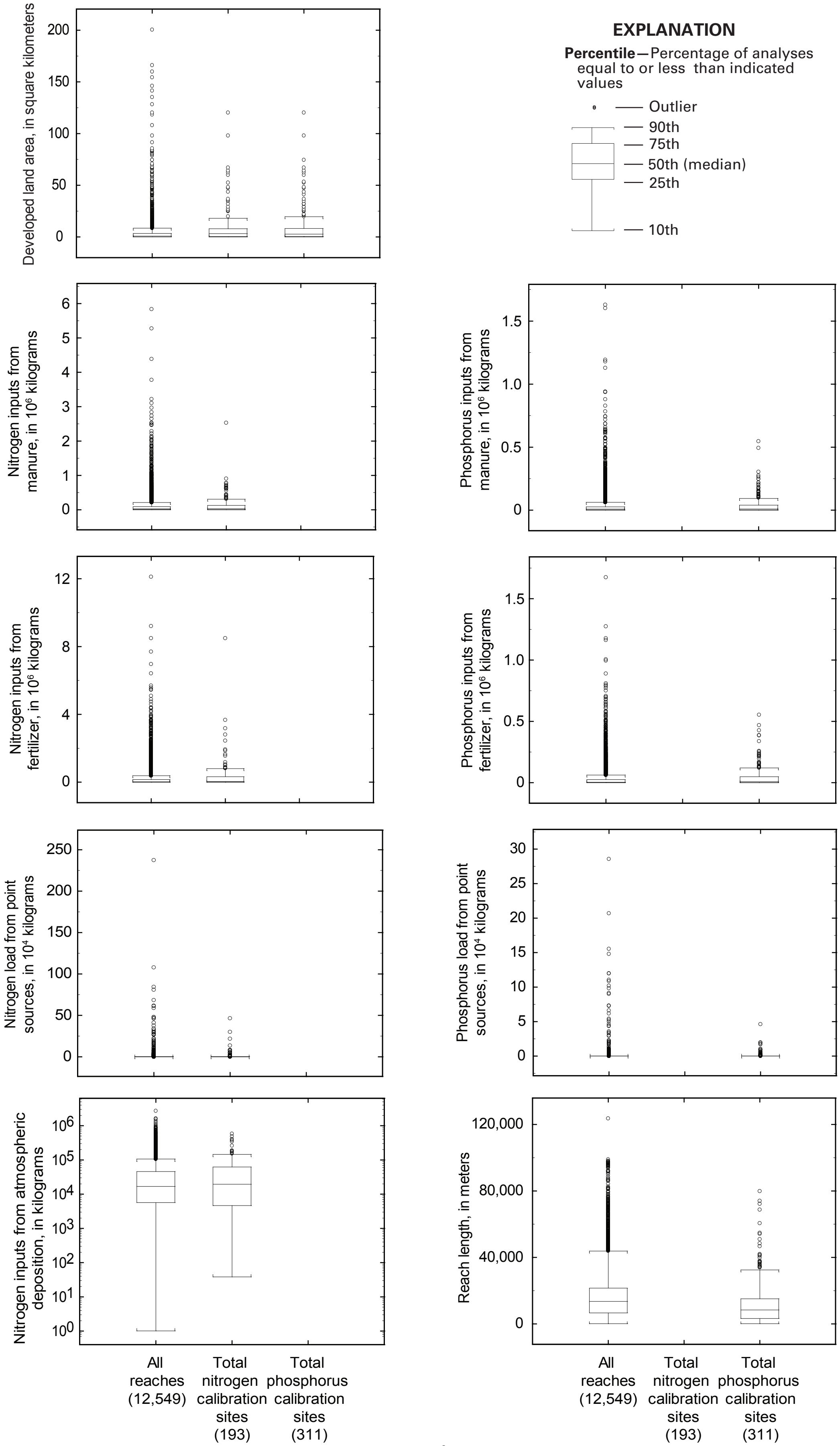

Figure S6b. Boxplots of basin characteristics for land-to-water delivery variables used in the TN and TP SPARROW models for the calibration reaches and all the reaches in the Missouri River Basin.

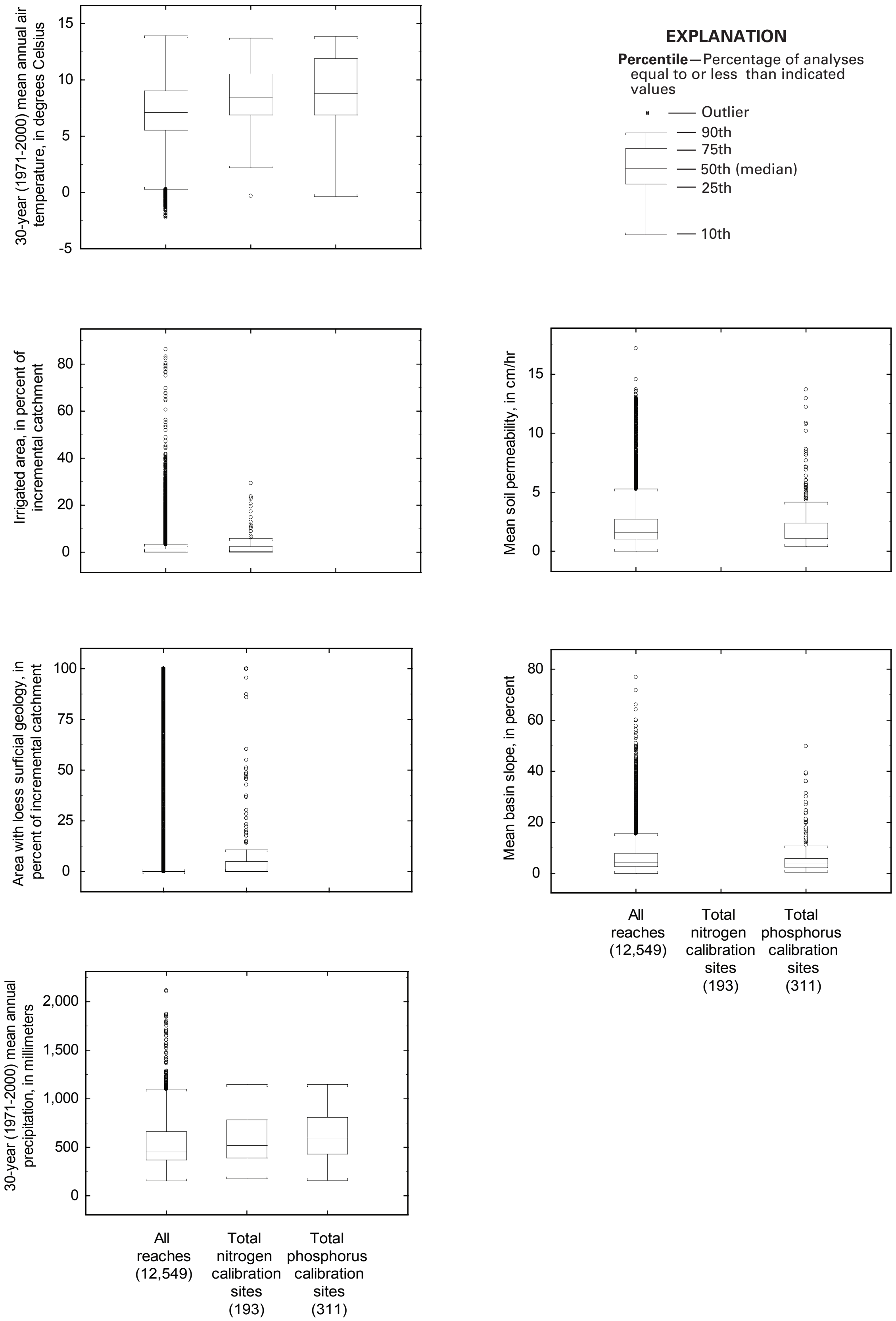

Point sources

Estimates of point-source contributions were derived using data from the U.S. Environmental Protection Agency (USEPA) Permit Compliance System (PCS) database (Maupin and Ivahnenko, this issue) (Figure S7). For the Missouri River Basin SPARROW models, only Standard Industrial Classifications (SIC) determined to discharge nitrogen or phosphorus to streams were used (Table S2). The estimates of point-source contributions used in model calibration were based on a mean of 1992, 1997, and 2002 data, as some inconsistencies in reporting between years were identified. For example, there were consistently fewer facilities reported for most SICs in the Missouri River Basin states in 1992 than in 1997 and 2002, and Missouri did not report any nutrient discharges in 1992. Minnesota had only one facility reported in 2002 while 12 and 14 facilities were reported as discharging nitrogen in 1992 and 1997, respectively. Variations on the 3-year mean (e.g., just 2002 data) were evaluated during model specification; however, the 3-year mean ultimately provided the best model fit. Typically, larger point sources for nitrogen (Figure S7a) coincide with the larger point sources for phosphorus (Figure S7b), with the largest point sources located in the urban areas of Denver, Colorado, and Lincoln, Nebraska. The PCS database included a greater density of point sources for some states (e.g., Colorado, Nebraska, and Iowa) than others (e.g., Missouri and Kansas). It is possible that these differences are due to differences in State reporting practices.

Figure S7. Estimates of nutrient contributions from point sources based on the mean of 1992, 1997, and 2002 data for (a) total nitrogen and (b) total phosphorus, in kilograms, in the Missouri River Basin.

(a) Total nitrogen

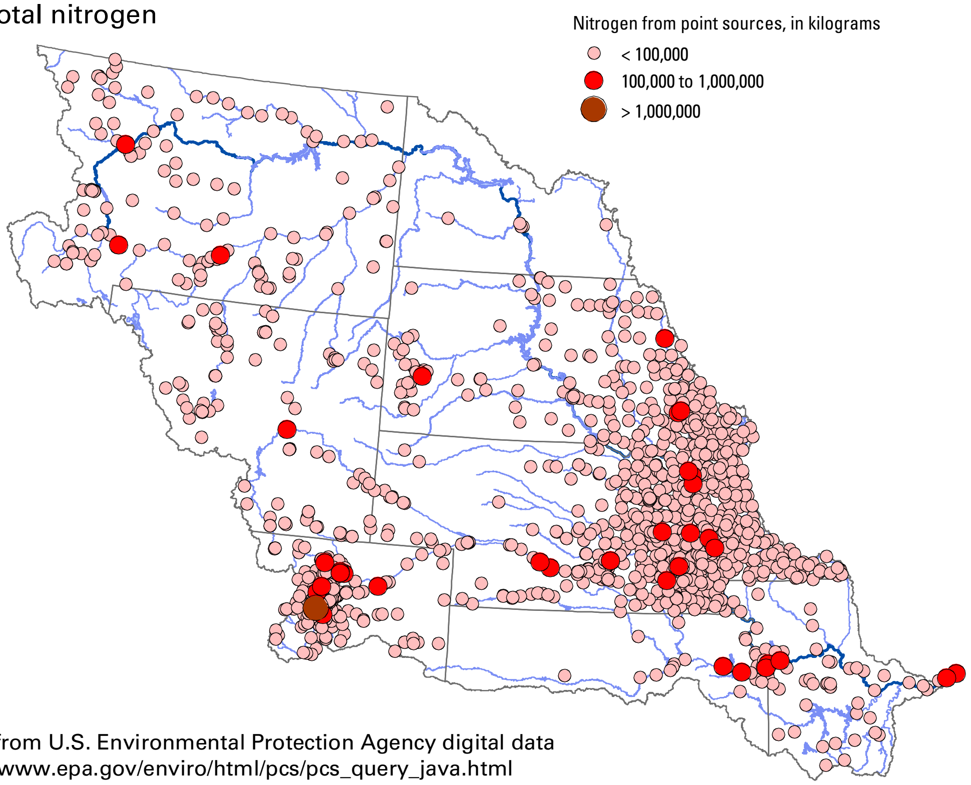

Base from U.S. Environmental Protection Agency digital data
http://www.epa.gov/enviro/html/pcs/pcs_query_java.html
Point source data from Maupin and Ivahnenko, this issue.

(b) Total phosphorus

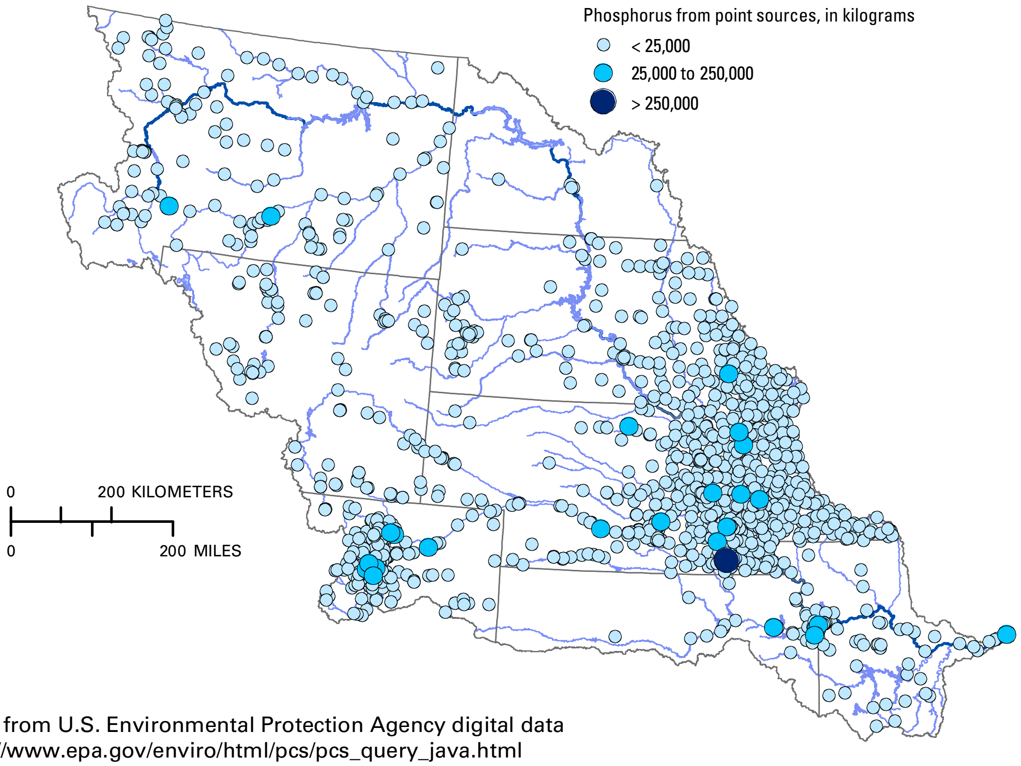

Base from U.S. Environmental Protection Agency digital data
http://www.epa.gov/enviro/html/pcs/pcs_query_java.html
Point source data from Maupin and Ivahnenko, this issue.

Table S2. List of Standard Industrial Classification codes included and excluded from the Missouri River Basin SPARROW models.

[SIC, Standard Industrial Classification code; *, number of facilities based on maximum number of facilities in 3 years of reporting (1992, 1997, or 2002)]

SIC	SIC Description	Number of point source facilities reporting nutrient discharges(*)
	Included in model	
0181	Ornamental Floriculture and Nursery Products	1
0211	Beef Cattle Feedlots	1
0921	Fish Hatcheries and Preserves	12
1442	Construction Sand and Gravel	26
1499	Miscellaneous Nonmetallic Minerals, except Fuels	2
1622	Bridge, Tunnel, and Elevated Highway Construction	1
2011	Meat Packing Plants	16
2013	Sausages and Other Prepared Meats	3
2015	Poultry Slaughtering and Processing	6
2021	Creamery Butter	1
2022	Natural, Processed, and Imitation Cheese	5
2024	Ice Cream and Frozen Desserts	1
2026	Fluid Milk	3
2046	Wet Corn Milling	2
2047	Dog and Cat Food	1
2048	Prepared Feed and Feed Ingredients for Animals and Fowls, except Dogs and Cats	1
2063	Beet Sugar	8
2068	Salted and Roasted Nuts and Seeds	1
2075	Soybean Oil Mills	5
2077	Animal and Marine Fats and Oils	1
2082	Malt Beverages	2
2085	Distilled and Blended Liquors	4
2086	Bottled and Canned Soft Drinks and Carbonated Waters	1
2097	Manufactured Ice	2
2099	Food Preparations, NEC	1
2542	Office and Store Fixtures, Partitions, Shelving, and Lockers, except Wood	2
2813	Industrial Gases	1
2819	Industrial Inorganic Chemicals, NEC	3
2821	Plastics Material and Synthetic Resins, and Nonvulcanizable Elastomers	1
2865	Cyclic Organic Crudes and Intermediates, and Organic Dyes and Pigments	1
2873	Nitrogenous Fertilizers	6
2875	Fertilizers, Mixing Only	1
2911	Petroleum Refining	10
3052	Rubber and Plastics Hose and Belting	3
3061	Molded, Extruded, and Lathe-Cut Mechanical Rubber Goods	2
3081	Unsupported Plastics Film and Sheet	2
3086	Plastics Foam Products	1
3089	Plastics Products, NEC	1
3312	Steel Works, Blast Furnaces (Including Coke Ovens), and Rolling Mills	1
3316	Cold-Rolled Steel Sheet, Strip, and Bars	2
3321	Gray and Ductile Iron Foundries	1
3339	Primary Smelting and Refining of Nonferrous Metals, except Copper and Aluminum	1
3341	Secondary Smelting and Refining of Nonferrous Metals	1
3423	Hand and Edge Tools, except Machine Tools and Handsaws	1
3431	Enameled Iron and Metal Sanitary Ware	1
3441	Fabricated Structural Metal	1
3442	Metal Doors, Sash, Frames, Molding, and Trim Manufacturing	1
3443	Fabricated Plate Work (Boiler Shops)	2
3462	Iron and Steel Forgings	1
3471	Electroplating, Plating, Polishing, Anodizing, and Coloring	6
3479	Coating, Engraving, and Allied Services, NEC	3

Table S2, cont. List of Standard Industrial Classification codes included and excluded from the Missouri River Basin SPARROW models.

[SIC, Standard Industrial Classification code; *, number of facilities based on maximum number of facilities in 3 years of reporting (1992, 1997, or 2002)]

SIC	SIC Description	Number of point source facilities reporting nutrient discharges(*)
	Included in model	
3482	Small Arms Ammunition	1
3483	Ammunition, except for Small Arms	1
3489	Ordnance and Accessories, NEC	1
3491	Industrial Valves	1
3492	Fluid Power Valves and Hose Fittings	1
3499	Fabricated Metal Products, NEC	2
3519	Internal Combustion Engines, NEC	1
3523	Farm Machinery and Equipment	4
3524	Lawn and Garden Tractors and Home Lawn and Garden Equipment	2
3533	Oil and Gas Field Machinery and Equipment	1
3535	Conveyors and Conveying Equipment	1
3548	Electric and Gas Welding and Soldering Equipment	1
3559	Special Industry Machinery, NEC	1
3566	Speed Changers, Industrial High-Speed Drives, and Gears	1
3585	Air-Conditioning and Warm Air Heating Equipment and Commercial and Industrial Refrigeration Equipment	2
3589	Service Industry Machinery, NEC	2
3612	Power, Distribution, and Specialty Transformers	1
3613	Switchgear and Switchboard Apparatus	1
3621	Motors and Generators	1
3625	Relays and Industrial Controls	4
3644	Noncurrent-Carrying Wiring Devices	1
3661	Telephone and Telegraph Apparatus	1
3675	Electronic Capacitors	2
3676	Electronic Resistors	1
3677	Electronic Coils, Transformers, and Other Inductors	1
3678	Electronic Connectors	1
3694	Electrical Equipment for Internal Combustion Engines	1
3714	Motor Vehicle Parts and Accessories	5
3715	Truck Trailers	2
3724	Aircraft Engines and Engine Parts	1
3743	Railroad Equipment	1
3751	Motorcycles, Bicycles, and Parts	1
3761	Guided Missiles and Space Vehicles	1
3799	Transportation Equipment, NEC	1
3823	Industrial Instruments for Measurement, Display, and Control of Process Variables; and Related Products	1
3824	Totalizing Fluid Meters and Counting Devices	1
3841	Surgical and Medical Instruments and Apparatus	3
3861	Photographic Equipment and Supplies	1
3999	Manufacturing Industries, NEC	1
4225	General Warehousing and Storage	1
4581	Airports, Flying Fields, and Airport Terminal Services	3
4613	Refined Petroleum Pipelines	4
4741	Rental of Railroad Cars	1
4789	Transportation Services, NEC	2
4812	Radiotelephone Communications	1
4813	Telephone Communications, except Radiotelephone	1
4939	Combination Utilities, NEC	1
4941	Water Supply	42
4952	Sewerage Systems	834

Table S2, cont. List of Standard Industrial Classification codes included and excluded from the Missouri River Basin SPARROW models.

[SIC, Standard Industrial Classification code; *, number of facilities based on maximum number of facilities in 3 years of reporting (1992, 1997, or 2002)]

SIC	SIC Description	Number of point source facilities reporting nutrient discharges(*)
	Included in model	
4953	Refuse Systems	6
4959	Sanitary Services, NEC	5
4961	Steam and Air-Conditioning Supply	2
5032	Brick, Stone and Related Construction Materials	1
5171	Petroleum Bulk Stations and Terminals	3
5399	Miscellaneous General Merchandise Stores	1
5511	Motor Vehicle Dealers (new and used)	2
5541	Gasoline Service Stations	6
6021	National Commercial Banks	1
6022	State Commercial Banks	1
6512	Operators of Nonresidential Buildings	4
7011	Hotels and Motels	3
7032	Sporting and Recreational Camps	1
7299	Miscellaneous Personal Services, NEC	1
7384	Photofinishing Laboratories	1
7542	Carwashes	1
7991	Physical Fitness Facilities	1
7992	Public Golf Courses	1
7993	Coin-Operated Amusement Devices	1
7996	Amusement Parks	1
7997	Membership Sports and Recreation Clubs	1
7999	Amusement and Recreation Services, NEC	6
8051	Skilled Nursing Care Facilities	1
8062	General Medical and Surgical Hospitals	1
8211	Elementary and Secondary Schools	3
8221	Colleges, Universities, and Professional Schools	1
8412	Museums and Art Galleries	1
8422	Arboreta and Botanical or Zoological Gardens	1
8711	Engineering Services	1
8731	Commercial Physical and Biological Research	1
8733	Noncommercial Research Organizations	1
8999	Services, NEC	1
9199	General Government, NEC	1
9224	Fire Protection	1
9511	Air and Water Resource and Solid Waste Management	1
9512	Land, Mineral, Wildlife, and Forest Conservation	1
9621	Regulation and Administration of Transportation Programs	1
9711	National Security	2
9999	Nonclassifiable Establishments	2
	Excluded from model	
1011	Iron ores	1
1021	Copper ores	1
1041	Gold ores	12
1099	Miscellaneous metal ores, NEC	1
1221	Bituminous coal and lignite surface mining	7
1311	Crude petroleum and natural gas	40
1321	Natural gas liquids	3
4922	Natural gas transmission	4
4911	Electric services	47

Nutrients from developed land area

Data on non-agricultural developed land area used in the models were derived from the 30-m grid of National Land Cover Database 2001 for the conterminous United States (LaMotte, 2008a,b,c,d) and allocated to MRB_E2RF1 catchments by Wieczorek and LaMotte (2010c). Developed land area in the Missouri River Basin includes four classes: developed open space, and low, medium, and high intensity developed land (Table S3). Developed land area in the Missouri River Basin generally increases following a west-northwest to east-southeast gradient, with less than 1% developed land area in many incremental catchments and as much as 100% developed land area in a few catchments (Figure S8). Notably, open-space developed land comprises as much as 100% of the total developed land area in many of the incremental catchments (Figure S9a), and it covers as much as 40% of the total catchment area in some incremental catchments (Figure S9b) making it a relevant component of nutrients being contributed from developed land area in the Missouri River Basin. The nutrient sources from developed land area may serve as a surrogate measure of various diffuse urban sources in the model, including subsurface inputs from individual or group septic systems as well as surface and subsurface runoff from fertilized land (e.g., golf courses, lawns, parks), runoff from impervious areas (e.g., rooftops, streets), nitrogen deposition associated with vehicle emissions of nitrous oxides, and inputs from domestic pets or wildlife.

Table S3. National Land Cover Database class definitions for developed land, 2001 (from http://www.mrlc.gov/nlcd_definitions.php, *accessed* June 2011).

Developed Land Class	Definition
Open space	Includes areas with a mixture of some constructed materials, but mostly vegetation in the form of lawn grasses. Impervious surfaces account for less than 20 percent of total cover. These areas most commonly include large-lot single-family housing units, parks, golf courses, and vegetation planted in developed settings for recreation, erosion control, or aesthetic purposes
Low intensity	Includes areas with a mixture of constructed materials and vegetation. Impervious surfaces account for 20-49 percent of total cover. These areas most commonly include single-family housing units.
Medium intensity	Includes areas with a mixture of constructed materials and vegetation. Impervious surfaces account for 50-79 percent of the total cover. These areas most commonly include single-family housing units.
High intensity	Includes highly developed areas where people reside or work in high numbers. Examples include apartment complexes, row houses and commercial/industrial. Impervious surfaces account for 80 to100 percent of the total cover.

Figure S8. Developed land (all classes) normalized by incremental catchment area, in percent, in the Missouri River Basin.

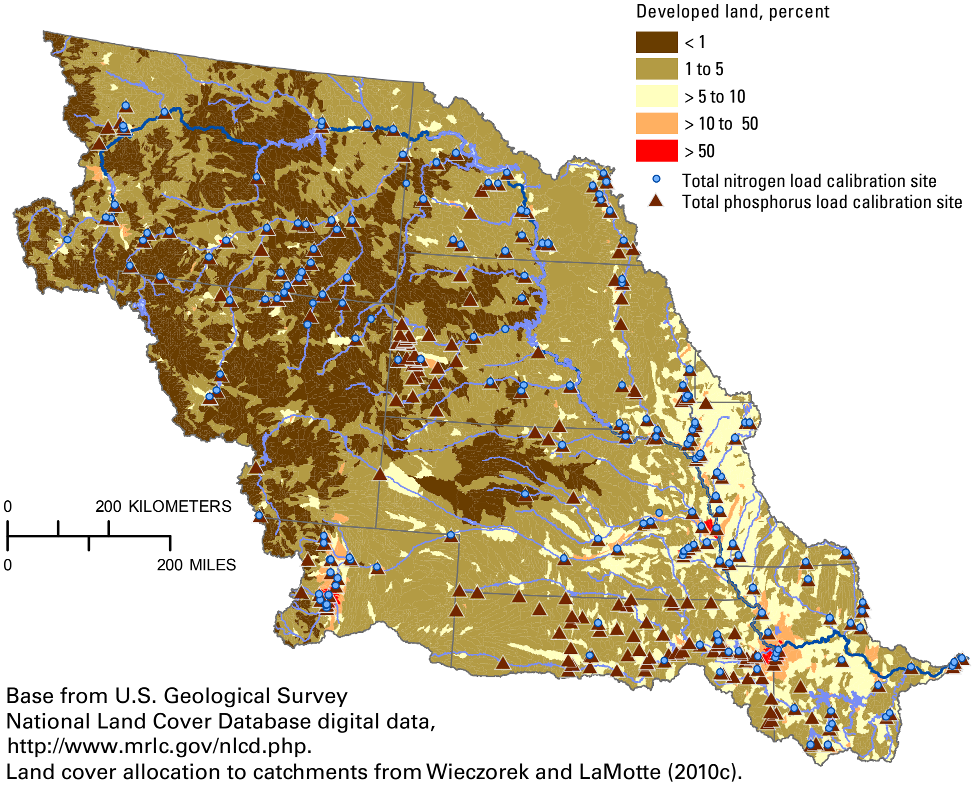

Base from U.S. Geological Survey
National Land Cover Database digital data,
http://www.mrlc.gov/nlcd.php.
Land cover allocation to catchments from Wieczorek and LaMotte (2010c).
Metadata on-line at http://water.usgs.gov/GIS/ metadata/usgswrd/XML/mrb_e2rf1_nlcd01.xml.

Figure S9. Open-space developed land (a) as a percent of total developed land area, and (b) normalized by incremental catchment area, in percent, in the Missouri River Basin, 2001.

(a) Open-space developed land, as percent of total developed land

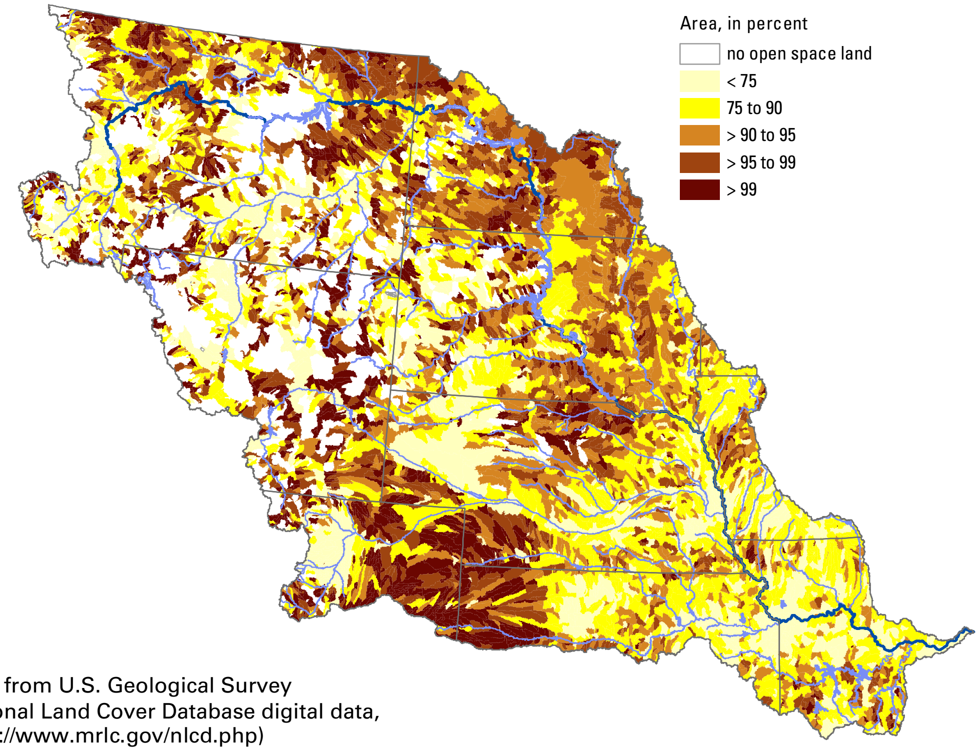

Base from U.S. Geological Survey
National Land Cover Database digital data,
(http://www.mrlc.gov/nlcd.php)
Land cover allocation to catchments from Wieczorek and LaMotte (2010c).
Metadata on-line at http://water.usgs.gov/GIS/metadata/usgswrd/XML/mrb_e2rf1_nlcd01.xml.

(b) Open-space developed land, normalized by incremental catchment area

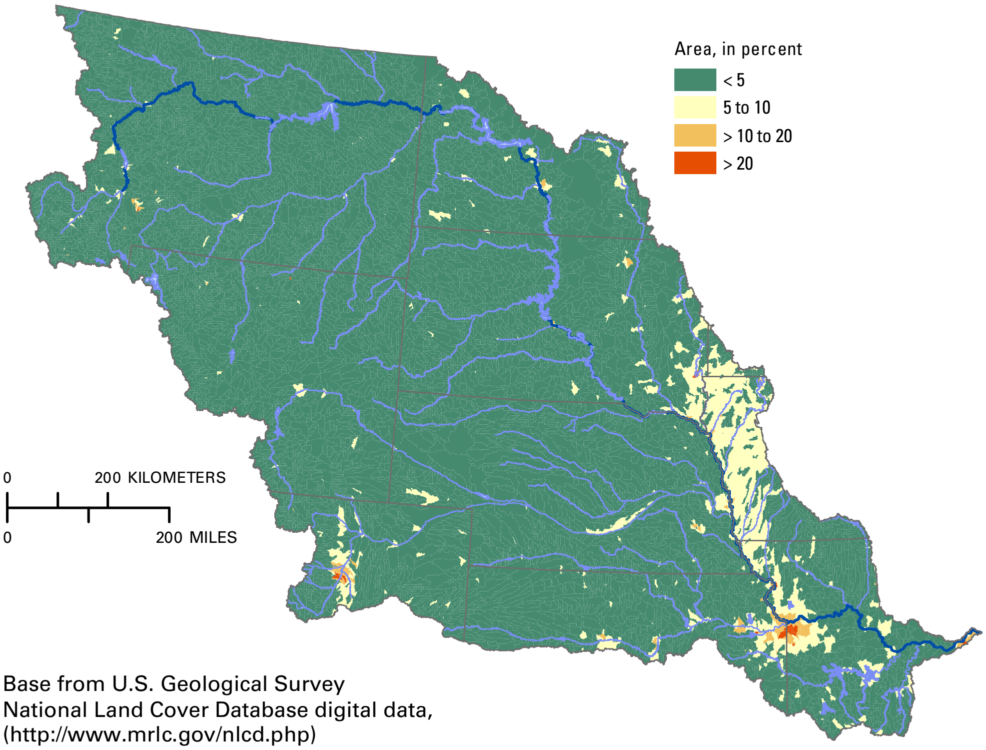

Base from U.S. Geological Survey
National Land Cover Database digital data,
(http://www.mrlc.gov/nlcd.php)
Land cover allocation to catchments from Wieczorek and LaMotte (2010c).
Metadata on-line at http://water.usgs.gov/GIS/metadata/usgswrd/XML/mrb_e2rf1_nlcd01.xml.

Nutrients from farm fertilizer

Data on nitrogen and phosphorus inputs from farm fertilizer were derived from 2002 sales and expenditures data from the Association of American Plant Food Control Officials and the U.S. Census of Agriculture (Ruddy *et al.*, 2006) and allocated MRB_E2RF1 catchments by Wieczorek and LaMotte (2010e). Farm fertilizer sales serve as a measure of the location and intensity of farming activities; in addition to providing a direct measure of commercial fertilizer use, the fertilizer source in SPARROW serves as a surrogate for other nutrient inputs to farms and the net effects of farm practices on nutrient runoff to the extent that they are spatially correlated with fertilizer sales. As a result, the model estimates of fertilizer contributions to streams may potentially reflect additional nutrient inputs to croplands from manure fertilizers and nitrogen fixation by legumes (e.g., soybeans, alfalfa) and the effects of some farm-management practices (e.g., rotations, harvesting, conservation tillage). Nutrient mineralization and immobilization rates in cultivated soils are assumed to be approximately in equilibrium (R.B. Alexander, U.S. Geological Survey, written commun., 2010). Farm fertilizer inputs showed a largely northwest to southeast increasing gradient (Figure S10). Fertilizer inputs were highest in the area directly west of Omaha and Lincoln, Nebraska, and northeast of Sioux City, Iowa (see Figure S1 for city locations). Spatial patterns of nitrogen (Figure S10a) and phosphorus (Figure S10b) fertilizer inputs were largely similar, though the magnitudes were substantially different. Generally, nitrogen inputs were at least an order of magnitude greater than phosphorus inputs.

Figure S10. Nutrient inputs per unit area from farm fertilizer as (a) total nitrogen and (b) total phosphorus, by county in kilograms per square kilometer, in the Missouri River Basin, 2002.

(a) Total nitrogen

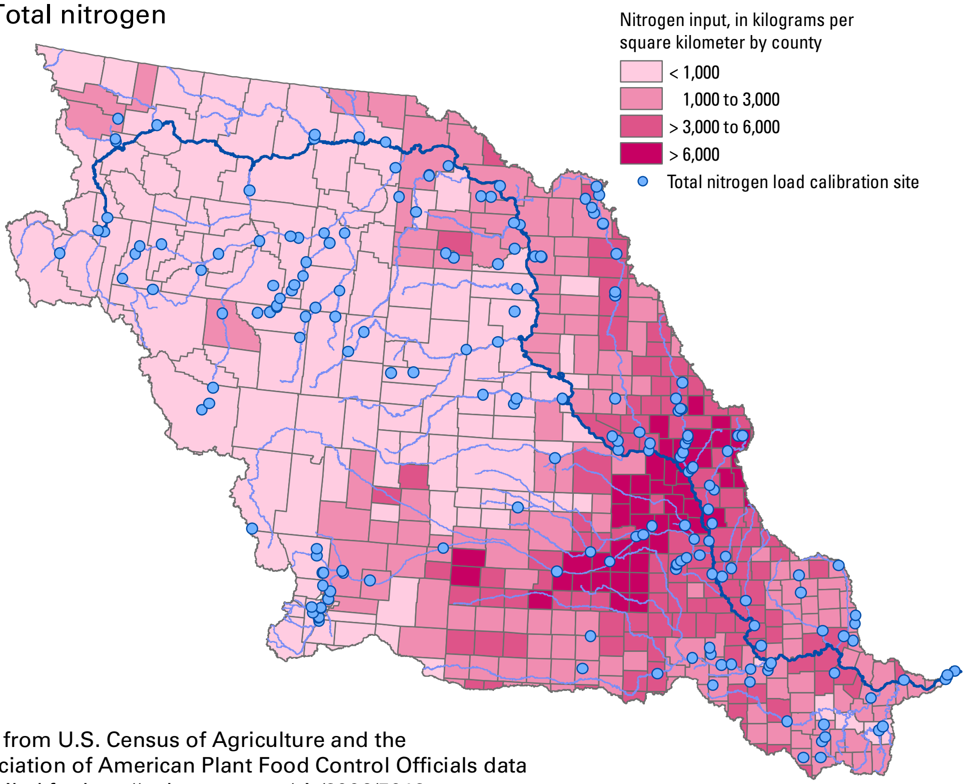

Base from U.S. Census of Agriculture and the Association of American Plant Food Control Officials data compiled for http://pubs.usgs.gov/sir/2006/5012.
Fertilizer allocation to catchments from Wieczorek and LaMotte (2010e).
Metadata on-line at http://water.usgs.gov/GIS/metadata/usgswrd/XML/mrb_e2rf1_nutrients.xml.

(b) Total phosphorus

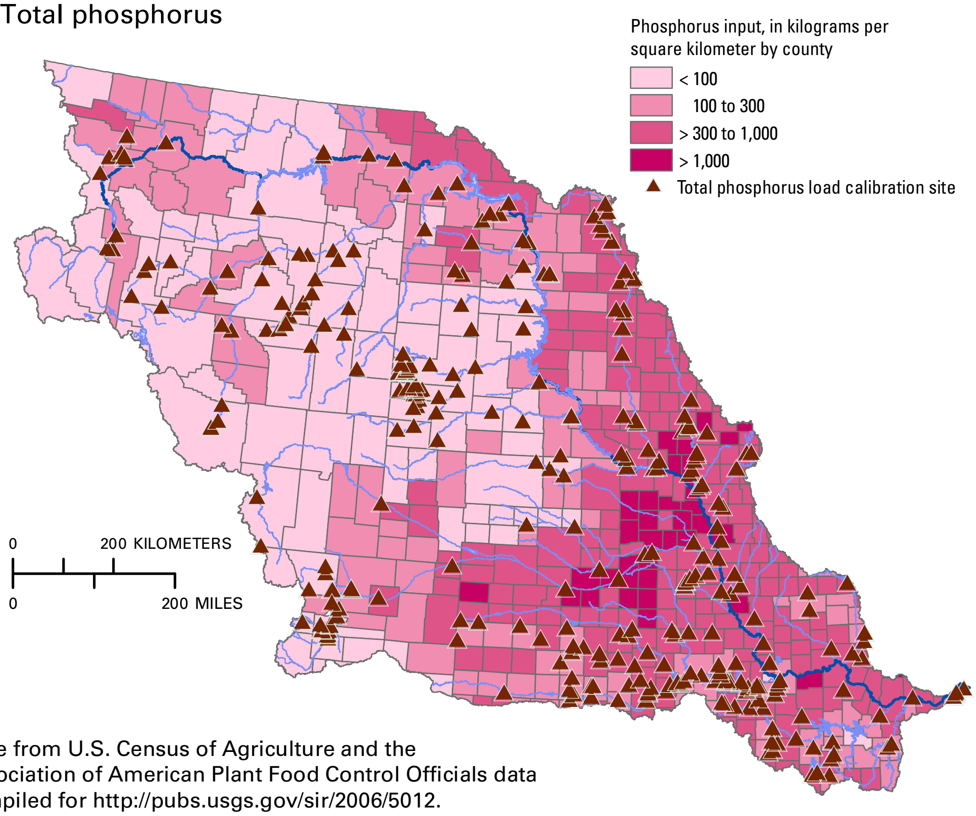

Base from U.S. Census of Agriculture and the Association of American Plant Food Control Officials data compiled for http://pubs.usgs.gov/sir/2006/5012.
Fertilizer allocation to catchments from Wieczorek and LaMotte (2010e).
Metadata on-line at http://water.usgs.gov/GIS/metadata/usgswrd/XML/mrb_e2rf1_nutrients.xml.

Nutrients from confined and unconfined manure

Data on nitrogen and phosphorus inputs from confined (predominantly from concentrated animal feeding operations for cattle, poultry, and dairy operations) and unconfined (farm-, pasture- and range- based livestock operations) manure were derived from 2002 livestock population data from the U.S. Census of Agriculture (Ruddy *et al.*, 2006) and allocated to MRB_E2RF1 catchments by Wieczorek and LaMotte (2010e). Confined animal wastes include recoverable manure that may be applied to nearby farmlands as well as unrecoverable manure that is lost during the collection, storage, and treatment of the waste. Manure inputs showed a northwest to southeast increasing gradient similar to that for fertilizer inputs (Figure S11). Manure inputs were greatest in the areas directly west of Omaha and Lincoln, Nebraska; northeast of Sioux City, Iowa; near Greeley, Colorado; and throughout much of the southern Lower Missouri River subbasin (see Figure S1 for city locations). Nitrogen inputs from manure (Figure S11a) were typically 2 to 4 times greater than phosphorus inputs from manure (Figure S11b) throughout the basin.

Figure S11. Nutrient inputs per unit area from confined and unconfined manure as (a) total nitrogen and (b) total phosphorus, by county in kilograms per square kilometer, in the Missouri River Basin, 2002.

(a) Total nitrogen

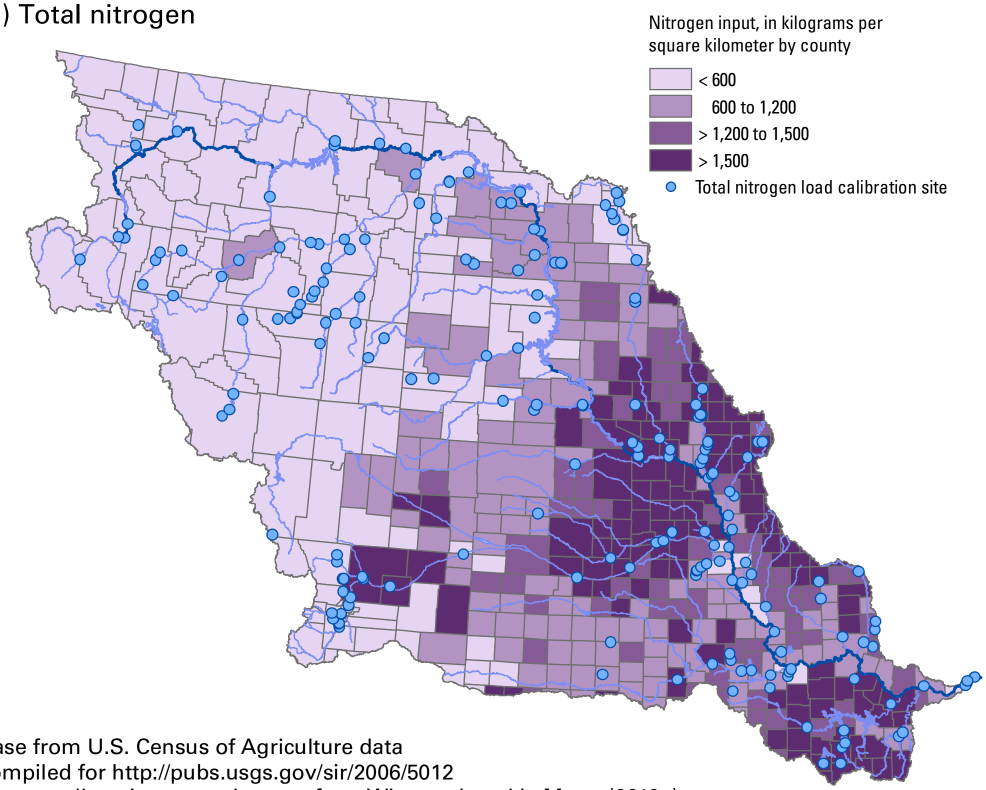

Base from U.S. Census of Agriculture data
compiled for http://pubs.usgs.gov/sir/2006/5012
Manure allocation to catchments from Wieczorek and LaMotte (2010e).
Metadata on-line at http://water.usgs.gov/GIS/metadata/usgswrd/XML/mrb_e2rf1_nutrients.xml.

(b) Total phosphorus

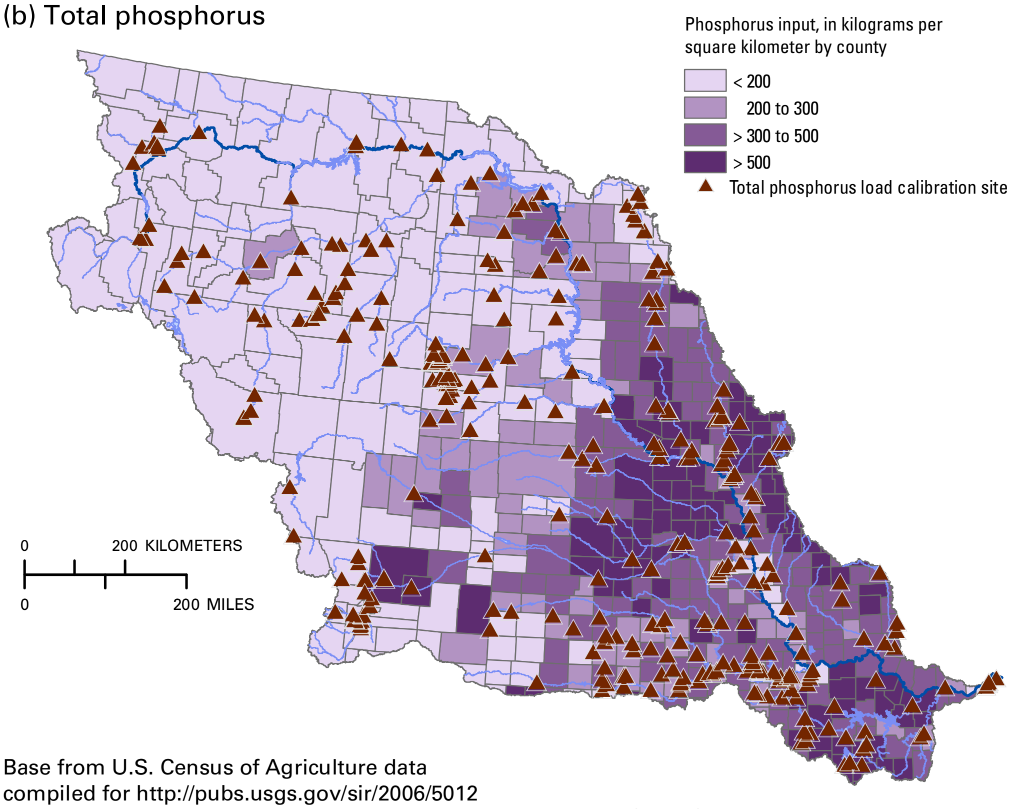

Base from U.S. Census of Agriculture data
compiled for http://pubs.usgs.gov/sir/2006/5012
Manure allocation to catchments from Wieczorek and LaMotte (2010e).
Metadata on-line at http://water.usgs.gov/GIS/metadata/usgswrd/XML/mrb_e2rf1_nutrients.xml.

Nitrogen from atmospheric deposition

Nitrogen inputs from atmospheric deposition were estimated from measurements of wet deposition of total inorganic nitrogen obtained from the National Atmospheric Deposition Program (NADP; http://nadp.sws.uiuc.edu/, *accessed* June 2011). These data were derived from long-term mean annual measurements (1990 to 2005) at 186 stations in the United States and serve as a surrogate for total (wet plus dry) inorganic nitrogen deposition. Estimates were detrended to the base year 2002 and allocated to MRB_E2RF1 catchments by Wieczorek and LaMotte (2010d). SPARROW estimates of the quantities of nitrogen deposition delivered to streams are expected to account for additional contributions from dry nitrogen deposition forms because the regional patterns of wet and dry deposition are generally correlated over large areas of the U.S. (Holland *et al.,* 2005; Baumgardner *et al.,* 2002). The SPARROW estimates of atmospheric nitrogen contributions to streams would also be expected to primarily reflect *regional* atmospheric nitrogen sources, given that NADP wet-deposition estimates generally reflect regional nitrous oxide (NOx) emissions from stationary sources (Elliott *et al.*, 2007). Local atmospheric nitrogen sources, such as those associated with vehicle emissions, are likely to be included in the SPARROW estimates of the nitrogen contributions from other modeled sources, especially developed land area.

Inputs from atmospheric deposition of nitrogen followed a largely west to east increasing gradient in the basin (Figure S12). The largest inputs occurred principally in the southeast part of the Missouri River Basin, particularly in the Grand River, Big Sioux, James River and lower Kansas River subbasins. These elevated inputs were likely the result of agricultural and urban influences concentrated in these areas. Additionally, nitrogen deposition appears to be elevated in the upper South Platte River subbasin. Evaluation of nitrogen emissions in the western United States suggest increasing nitrate deposition in the Rocky Mountains (Nilles and Conley, 2001; Fenn *et al.*, 2003; Campbell, 2003), and recent research suggests a correlation between elevated regional anthropogenic emissions of nitrogen compounds and increasing nitrate concentrations in some high-elevation lakes in the Rocky Mountains (Nanus *et al.*, 2008).

Figure S12. Nitrogen inputs from atmospheric deposition from measurements of wet deposition of total inorganic nitrogen, in kilograms per square kilometer per year, in the Missouri River Basin, 2002.

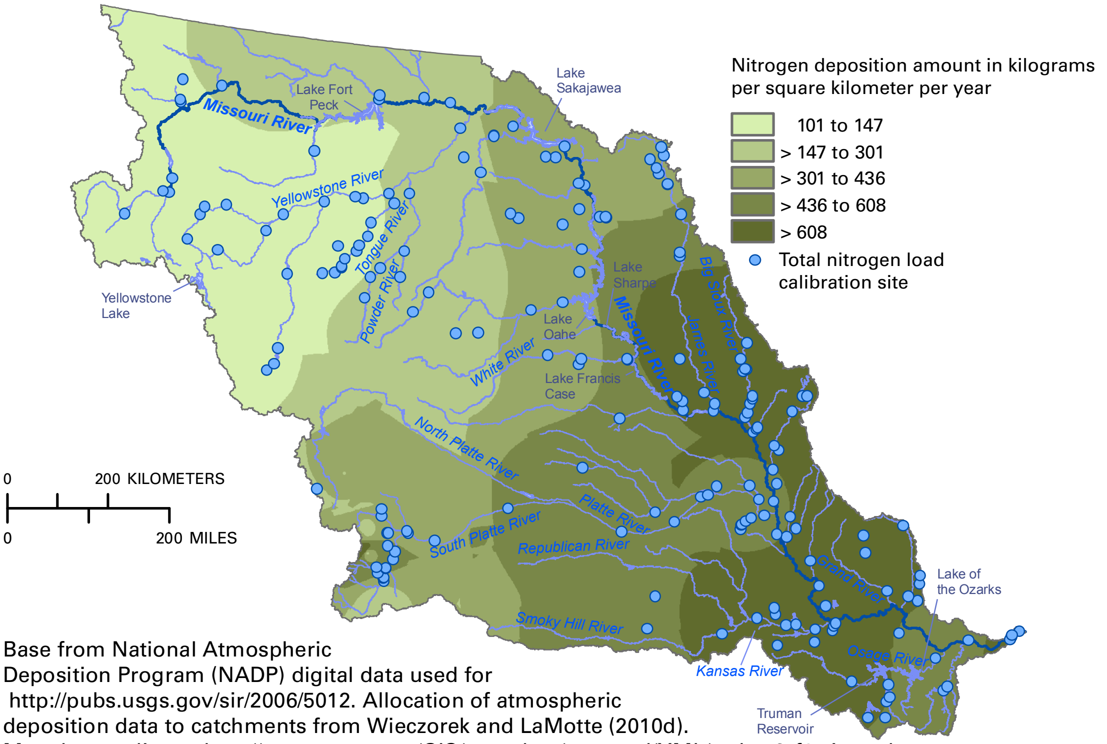

Base from National Atmospheric Deposition Program (NADP) digital data used for http://pubs.usgs.gov/sir/2006/5012. Allocation of atmospheric deposition data to catchments from Wieczorek and LaMotte (2010d). Metadata on-line at http://water.usgs.gov/GIS/metadata/usgswrd/XML/mrb_e2rf1_tin.xml. Data represents the average normalized atmospheric (wet) deposition of total inorganic nitrogen for 2002.

Stream channels as source of phosphorus

Stream channel length estimates, used as a surrogate for stream channel sources of phosphorus, were derived from reach lengths in the modeled MRB_E2RF1 stream network (Nolan *et al.*, 2002; Wieczorek and LaMotte, 2010b) for streams with discharge greater than 1.13 m^3/s (40 ft^3/s) (those found to be a significant source of phosphorus in the TP model). Shorter channel lengths (< 20 km) meeting the model discharge criterion were largely concentrated along the western boundary of the basin, while medium and longer-length channels meeting the discharge criterion were concentrated in the southeast part of the basin (Figure S13).

Figure S13. Stream channel length as a source of phosphorus (for streams where discharge is greater than 1.13 cubic meters per second), in kilometers, in the Missouri River Basin.

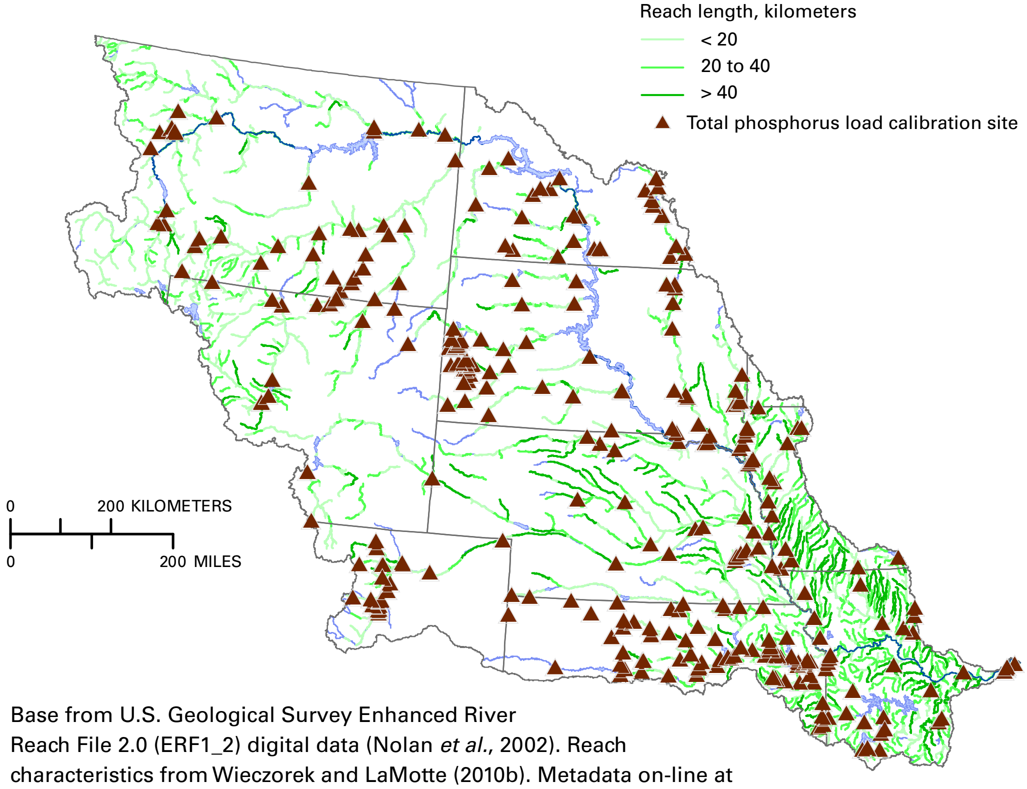

Base from U.S. Geological Survey Enhanced River Reach File 2.0 (ERF1_2) digital data (Nolan *et al.*, 2002). Reach characteristics from Wieczorek and LaMotte (2010b). Metadata on-line at http://water.usgs.gov/GIS/metadata/usgswrd/XML/mrb_e2rf1_bchar.xml.

Mean annual precipitation

Precipitation data averaged from annual precipitation depth values over 30 years (1971 to 2002) were obtained from the Parameter-elevation Regressions on Independent Slopes Model (PRISM) digital data network (http://www.prism.oregonstate.edu/, *accessed* July 23, 2009) and allocated to MRB_E2RF1 catchments by Wieczorek and LaMotte (2010j). These data show a strong west to east gradient of increasing mean precipitation in the Missouri River Basin (Figure S14). The exception to this gradient is a relatively narrow band of higher precipitation that follows the western boundary of the basin in the Rocky Mountains. Mean annual precipitation values for 2002 were also evaluated in the models; however, the 30-year long-term mean provided a better fit in both the TN and TP SPARROW models.

Figure S14. Mean annual precipitation, in millimeters, in the Missouri River Basin, 1971-2000.

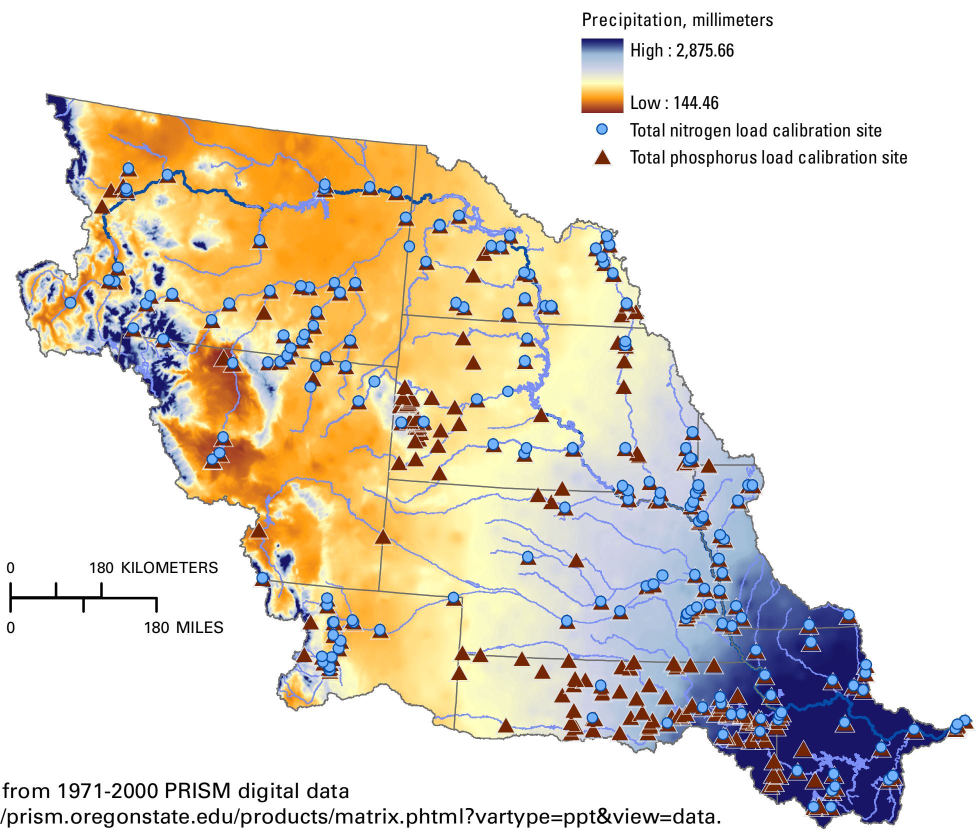

Base from 1971-2000 PRISM digital data
http://prism.oregonstate.edu/products/matrix.phtml?vartype=ppt&view=data.
Allocation of precipitation to catchments from Wieczorek and LaMotte (2010j).
Metadata on-line at http://water.usgs.gov/GIS/metadata/usgswrd/XML/mrb_e2rf1_ppt30yr.xml.

Mean air temperature

Air temperature data averaged from minimum and maximum daily temperature values over 30 years (1971 to 2000) were obtained from the Parameter-elevation Regressions on Independent Slopes Model (PRISM) digital data network (http://www.prism.oregonstate.edu/, *accessed* July 23, 2009) and allocated to MRB_E2RF1 catchments by Wieczorek and LaMotte (2010h,i). These data generally show a northwest to southeast gradient of increasing mean air temperature in the Missouri River Basin (Figure S15). The exceptions to this spatial pattern are the lower temperatures along the western boundary of the basin in the Rocky Mountains and the northern boundary of the basin. Minimum and maximum daily air temperature values for 2002 were also evaluated in the models; however, the 30-year long-term mean provided a better fit in both the TN and TP SPARROW models.

Figure S15. Mean of maximum and minimum air temperature, in degrees Celsius, in the Missouri River Basin, 1971-2000.

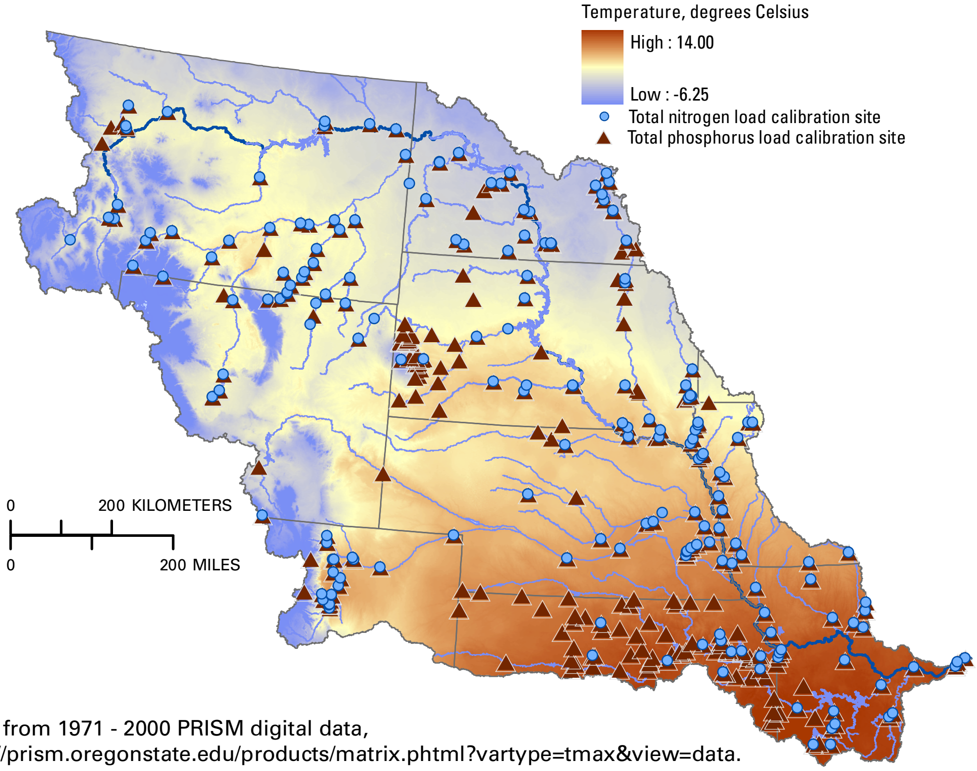

Base from 1971 - 2000 PRISM digital data,
http://prism.oregonstate.edu/products/matrix.phtml?vartype=tmax&view=data.
Allocation of temperature values to catchments from Wieczorek and LaMotte (2010h,i).
Metadata on-line at http://water.usgs.gov/GIS/metadata/usgswrd/XML/mrb_e2rf1_tmax30yr.xml,
and http://water.usgs.gov/GIS/metadata/usgswrd/XML/mrb_e2rf1_tmin30yr.xml.

Estimated irrigated agricultural land

Combined estimates of the agricultural area in gravity, pressure, and gravity and pressure irrigation (categories not distinguished in the model dataset) were derived from the 1997 National Resources Inventory (NRI) dataset created by the National Resource Conservation Service (http://www.nrcs.usda.gov/technical/NRI/, *accessed* April 26, 2011) and allocated to MRB_E2RF1 catchments by Wieczorek and LaMotte (2010a). Regional or national-scale irrigation datasets with more refined detail on irrigation type and geographic extent are not currently available. These NRI estimates performed similarly in the TN model as alternative mean irrigation estimates computed for the Missouri River Basin catchments from the 2002 National Agricultural Statistics Service dataset (http://www.agcensus.usda.gov/Publications/2002/index.asp, *accessed* April 26, 2011). The NRI data indicate that most of the irrigated agricultural is concentrated in areas north of and surrounding Lake Sharpe, Lake Francis Case, and Lewis and Clark Lake, and the eastern Platte River and northwestern Kansas River subbasins. Large areas of Federal land and other areas with minimal irrigation characterize a large part of the northern and western part of the study area (Figure S16).

Figure S16. Estimated agricultural area in gravity and pressure irrigation, in percent, in the Missouri River Basin, 1997.

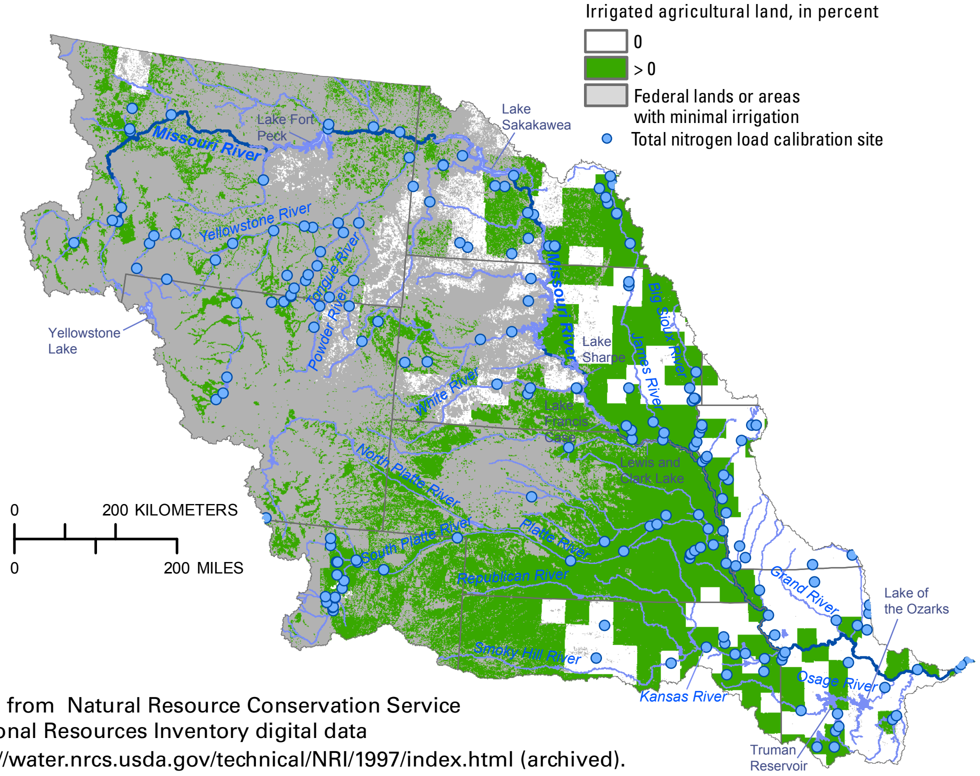

Base from Natural Resource Conservation Service
National Resources Inventory digital data
http://water.nrcs.usda.gov/technical/NRI/1997/index.html (archived).
Allocation of irrigation data to catchments from Wieczorek and LaMotte (2010a).
Metadata on-line at http://water.usgs.gov/GIS/metadata/usgswrd/XML/mrb_e2rf1_adrain.xml.

Loess-dominated geologic units

Loess-dominated geologic units were derived from surficial geology data digitally generated from the USGS National Atlas map series (Hunt, 1979) for the USGS National Water-Quality Assessment Program (Clawges and Price, 1999) and allocated to MRB_E2RF1 catchments by Wieczorek and LaMotte (2010g). The loess-dominated units identified in the Missouri River Basin are a combination of three surficial geologic units, including Wisconsinan loess (wl), deeply-weathered loess (es), and sandy or silty residuum, probably including loess (rsi). An alternative loess-based variable that excluded the rsi unit was tested in the preliminary model regressions, but it was not found to be as accurate of a predictor of nitrogen transport in the model. The distribution of loess-dominated surficial geology by major subbasin varies from 0 to 54% of the total drainage area in a subbasin (Table S4). The Lower Middle Missouri and the Lower Missouri River subbasins have the highest percentage of loess-dominated surficial geology (46 to 54%). These loess-dominated areas only occur in the southeastern part of the basin (Figure S17).

Table S4. Summary of loess-dominated surficial geology by major subbasin[1].

[km^2, square kilometers; %, percent]

Major subbasin	Total drainage area [2](km^2)	Total loess area (km^2)	Loess in subbasin as percentage of total loess in Missouri River Basin (%)	Loess in subbasin as percentage of total drainage area in subbasin (%)
Upper Missouri	242,208	0	0	0
Yellowstone	181,603	0	0	0
Middle Missouri	405,807	28,472	16	7
Platte	221,530	37,511	21	17
Lower Middle Missouri	35,241	18,866	10	54
Kansas	155,577	48,061	27	31
Lower Missouri	103,433	47,384	26	46
Missouri River Basin	1,345,399[(3)]	180,294[(3)]	100	13

[1] Surficial geology data allocated to catchments from Wieczorek and LaMotte (2010g).

[2] Drainage areas (that is, incremental catchments) used in SPARROW models for the stream

[3] Excludes Oldman River drainage area.

Figure S17. Loess-dominated surficial geology in the Missouri River Basin.

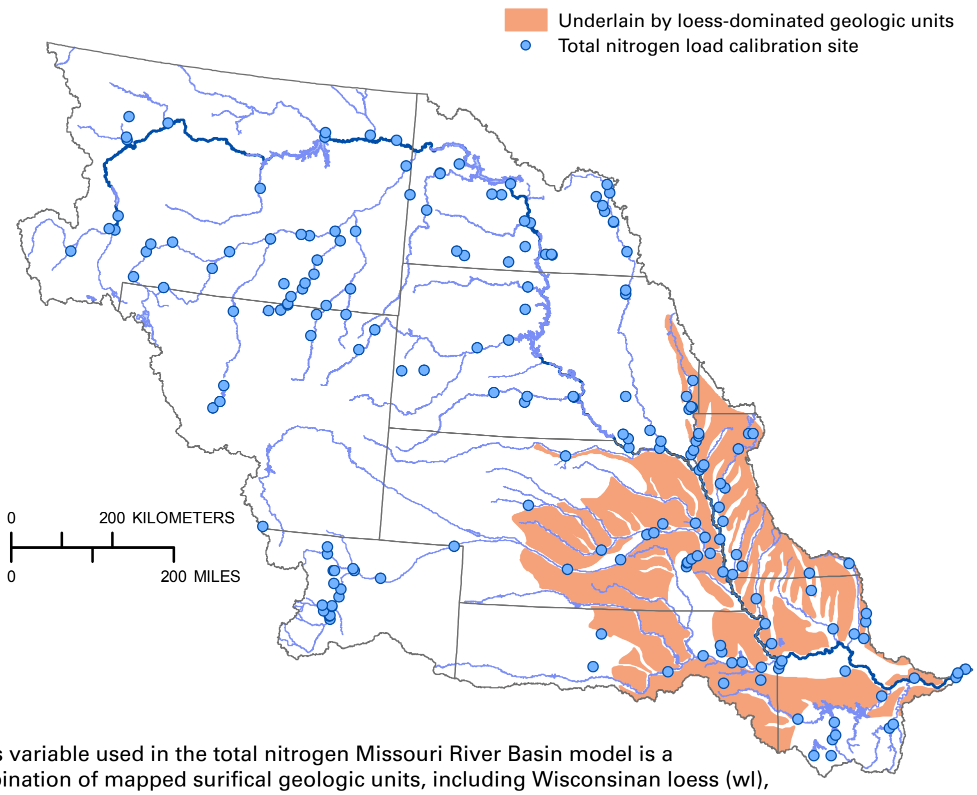

Loess variable used in the total nitrogen Missouri River Basin model is a combination of mapped surifical geologic units, including Wisconsinan loess (wl), deeply-weathered loess (es), and sandy or silty residuum, probably including loess (rsi). Allocation of surficial geology to catchments from Wieczorek and LaMotte (2010g). Metadata on-line at http://water.usgs.gov/GIS/metadata/usgswrd/XML/mrb_e2rf1_sgeol.xml.

Mean soil permeability

Mean soil permeability was derived from the 1994 State Soil Geographic (STATSGO) Data Base digital data (Wolock, 1997) and allocated to MRB_E2RF1 catchments by Wieczorek and LaMotte (2010f). Soil permeability shows a largely variable pattern throughout the Missouri River Basin (Figure S18). The area of highest permeability is in the Sand Hills region of central Nebraska, which forms the headwaters of the Dismal River and surrounding tributaries to the Platte River (see Figure 1 in paper for stream locations). Permeability is also higher along the western boundary of the basin, except in northwestern Montana. Areas of low permeability include northern Montana, central and eastern South Dakota, eastern Kansas, and northwestern Missouri.

Figure S18. Mean soil permeability, in centimeters per hour, in the Missouri River Basin.

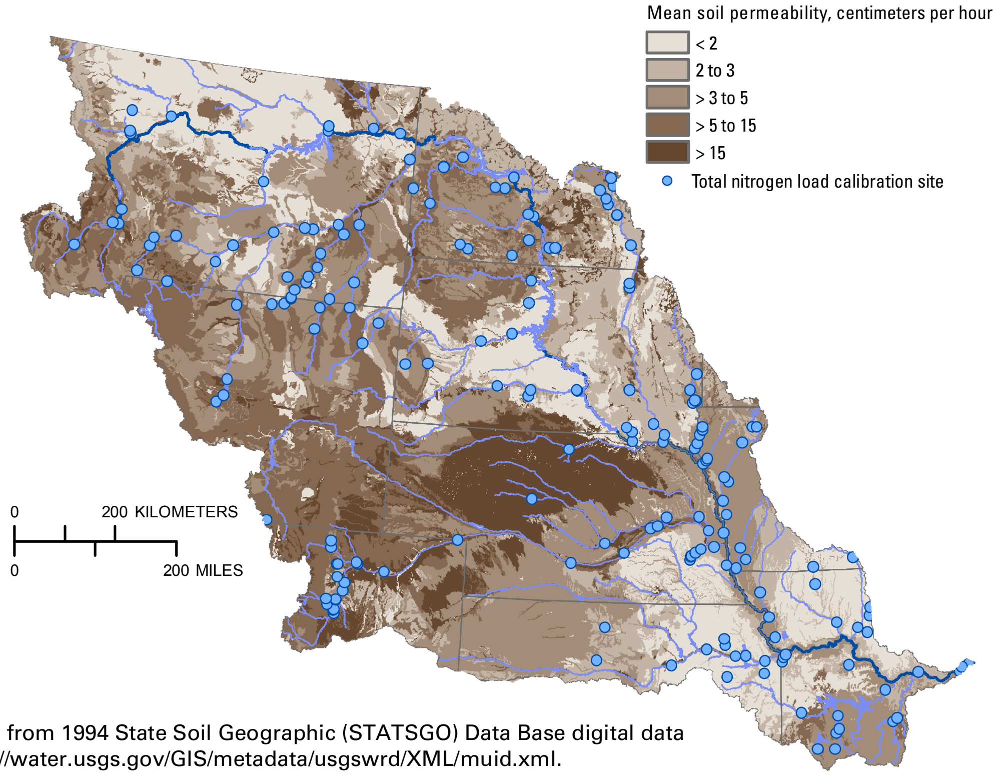

Base from 1994 State Soil Geographic (STATSGO) Data Base digital data
http://water.usgs.gov/GIS/metadata/usgswrd/XML/muid.xml.
Allocation of soil permeability to catchments from Wieczorek and LaMotte (2010f).
Metadata on-line at http://water.usgs.gov/GIS/metadata/usgswrd/XML/mrb_e2rf1_statsgo.xml.

Mean basin slope

Mean basin slope (Figure S19) in the Missouri River Basin was derived from basin characteristics compiled by Wieczorek and LaMotte (2010a) for the MRB_E2RF1 stream reach network that was modified from the U.S. Environmental Protection Agency's Enhanced River Reach File 2.0 (ERF1_2) (Nolan *et al.*, 2002; Brakebill and Terziotti, 2011; Brakebill *et al.*, this issue). Generally slope is highest in the headwater regions in the Northern and Southern Rocky Mountains and lowest in the northeast part of the Middle Missouri River subbasin and in the western and north-central parts of the Kansas River subbasin.

Figure S19. Mean basin slope, in percent, in the Missouri River Basin.

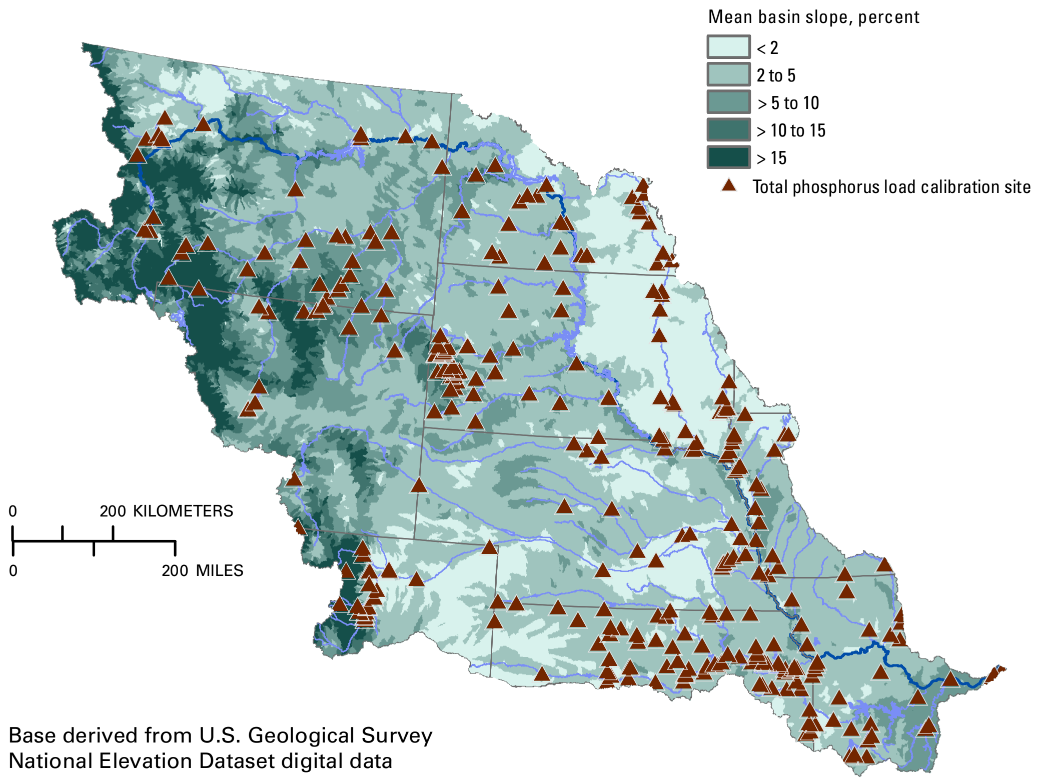

Base derived from U.S. Geological Survey
National Elevation Dataset digital data
Metadata on-line at http://water.usgs.gov/
GIS/metadata/usgswrd/XML/mrb_e2rf1_bchar.xml.

Supporting Information Literature Cited

Alexander, R.B., R.A. Smith, G.E. Schwarz, E.W. Boyer, J.V. Nolan, and J.W. Brakebill, 2008. Differences in Phosphorus and Nitrogen Delivery to the Gulf of Mexico From the Mississippi River. Environ. Sci. Technol. 42(3):822-830 and supporting information, doi:10.1021/es0716103

Baumgardner, R.E., T.F. Lavery, C.M. Rogers, and S.S. Isil, 2002. Estimates of the Atmospheric Deposition of Sulfur and Nitrogen Species: Clean Air Status and Trends Network, 1990-2000. Environ. Sci. Technol., 36(12):2614-2629, doi:10.1021/es011146g

Brakebill, J.W. and S.E. Terziotti, 2011. A Digital Hydrologic Network Supporting NAWQA MRB SPARROW Modeling – MRB_E2RF1 (Version 1.0). U.S. Geological Survey General Information (web only). http://water.usgs.gov/GIS/metadata/usgswrd/XML/mrb_e2rf1.xml, *accessed* June 27, 2011.

Brakebill, J.W., D.M. Wolock, and S.E. Terziotti, this issue. Digital Hydrologic Networks Supporting Applications Related to Spatially Referenced Regression Modeling. Journal of the American Water Resources Association, doi: 10.1111/j.1752-1688.2011.00578.x

Campbell, D.H., 2003. Atmospheric Deposition and Its Effects in the Intermountain West. *In:* Acid Rain: Are the Problems Solved? American Fisheries Society Trends in Fisheries and Management 2. J.C. White (Editor). American Fisheries Society, Bethesda, MD, 260 p. ISBN 1888569476

Clawges, R. and C. Price, 1999. Digital Data Set Describing Surficial Geology in the Conterminous United States. U.S. Geological Survey Open-File Report 99-77. http://water.usgs.gov/GIS/metadata/usgswrd/XML/ofr99-77_geol75m.xml, *accessed* June 27, 2011.

Elliott, E.M., C. Kendall, S.D. Wankel, D.A. Burns, E.W. Boyer, K. Harlin, D.J. Bain, and T.J. Butler, 2007. Nitrogen Isotopes as Indicators of NOx Sources Contributions to Atmospheric Nitrate Deposition Across the Midwestern and Northeastern United States. Environ. Sci. Technol., 41(22):7661-7667, doi:10.1021/es070898t

Fenn, M.E., R. Haeuber, G.S. Tonnesen, J.S. Baron, S. Grossman-Clarke, D. Hope, D.A. Jaffe, S. Copeland, L. Geiser, H.M. Rueth, and J.O. Sickman, 2003. Nitrogen Emissions, Deposition, and Monitoring in the Western United States, BioScience 53(4):391-403, doi:10.1641/0006-3568(2003)053[0391:NEDAMI]2.0.CO;2

Fenneman, N.M. and D.W. Johnson, 1946. Physiographic Divisions of the Conterminous United States: U.S. Geological Survey map, scale 1:7,000,000. http://water.usgs.gov/GIS/metadata/usgswrd/XML/physio.xml, *accessed* June 27, 2011.

Holland, E.A., B.H. Braswell, J. Sulzman, and J.F. Lamarque, 2005. Nitrogen Deposition on the United States and Western Europe: Synthesis of Observations and Models. Ecological Applications, 15(1):38-57, doi:10.1890/03-5162.

Hunt, C.D., 1979. National Atlas of the United States of America – Surficial Geology. U.S. Geological Survey, NAC-P-0204-75M-0 [map].

LaMotte, A.E., 2008a. National Land Cover Database 2001 (NLCD01) Tile 1, Northwest United States: NLCD01_1. U.S. Geological Survey Data Series DS-383A. http://water.usgs.gov/GIS/metadata/usgswrd/XML/nlcd01_1.xml, *accessed* June 27, 2011.

LaMotte, A.E., 2008b. National Land Cover Database 2001 (NLCD01) Tile 2, Northeast United States: NLCD01_2. U.S. Geological Survey Data Series DS-383B. http://water.usgs.gov/GIS/metadata/usgswrd/XML/nlcd01_2.xml, *accessed* June 27, 2011.

LaMotte, A.E., 2008c. National Land Cover Database 2001 (NLCD01) Tile 3, Southwest United States: NLCD01_3. U.S. Geological Survey Data Series DS-383C. http://water.usgs.gov/GIS/metadata/usgswrd/XML/nlcd01_3.xml, *accessed* June 27, 2011.

LaMotte, A.E., 2008d. National Land Cover Database 2001 (NLCD01) Tile 4, Southeast United States: NLCD01_4. U.S. Geological Survey Data Series DS-383D. http://water.usgs.gov/GIS/metadata/usgswrd/XML/nlcd01_4.xml, *accessed* June 27, 2011.

Maupin, M.A., and T. Ivahnenko, this issue. Nutrient Loadings to Streams of the Continental United States From Municipal and Industrial Effluent. Journal of the American Water Resources Association, doi: 10.1111/j.1752-1688.2011.00576.x

Nanus, L., M.W. Williams, D.H. Campbell, E.M. Elliott, and C. Kendall, 2008. Evaluating Regional Patterns in Nitrate Sources to Watersheds in National Parks of the Rocky Mountains Using Nitrate Isotopes. Environ. Sci. Technol. 42(17):6487-6493, doi:10.1021/es800739e

Nilles, M.A., and Brooke E. Conley, 2001. Changes in the Chemistry of Precipitation in the United States, 1981-1998. Water, Air, and Soil Pollution 130(1-4):409-414, doi:10.1023/A:1013889302895

Nolan, J.V., J.W. Brakebill, R.B. Alexander, and G.E. Schwarz, 2002. ERF1_2 – Enhanced River Reach File 2.0 (Version 2.0). U.S. Geological Survey Open-File Report 02-40. http://water.usgs.gov/GIS/metadata/usgswrd/XML/erf1_2.xml, *accessed* June 27, 2011.

Ruddy, B.C., D.L. Lorenz, and D.K. Mueller, 2006. County-Level Estimates of Nutrient Inputs to the Land Surface of the Conterminous United States, 1982-2001. U.S. Geological Survey Scientific Investigations Report 2006-5012. http://pubs.usgs.gov/sir/2006/5012/, *accessed* June 27, 2011.

Saad, D.A., G.E. Schwarz, D.M. Robertson, and N.L. Booth, this issue. A Multi-Agency Nutrient Dataset Used to Estimate Loads, Improve Monitoring Design, and Calibrate Regional Nutrient SPARROW Models. Journal of the American Water Resources Association, doi: 10.1111/j.1752-1688.2011.00575.x

Schwarz, G.E., A.B. Hoos, R.B. Alexander, and R.A. Smith, 2006. The SPARROW Surface Water-Quality Model – Theory, Applications and User Documentation. U.S. Geological Survey Techniques and Methods 6-B3, 248 p. and CD-ROM. http://pubs.usgs.gov/tm/2006/tm6b3/, *accessed* June 27, 2011.

Smith, R.A., G.E. Schwarz, and R.B. Alexander, 1997. Regional Interpretation of Water-Quality Monitoring Data. Water Resources Research 33(12):2781-2798, doi:10.1029/97WR02171

Sprague, L.A., M.L. Clark, D.L. Rus, R.B. Zelt, J.L. Flynn, and J.V. Davis, 2006. Nutrient and Suspended-Sediment Trends in the Missouri River Basin, 1993-2003. U.S. Geological Survey Scientific Investigations Report 2006-5321, 80 p. http://pubs.usgs.gov/sir/2006/5231/pdf/SIR06-5231_508.pdf, *accessed* June 27, 2011.

U.S. Army Corps of Engineers in cooperation with FEMA's National Dam Safety Program, 2005, National Inventory of Dams, Water Control Infrastructure. U.S. Army Corps of Engineers, http://crunch.tec.army.mil/nidpublic/webpages/nid.cfm, *accessed* January, 2008.

Wieczorek, M.E. and A.E. LaMotte, 2010a. Attributes for MRB_E2RF1 Catchments By Major River Basins in the Conterminous United States: Artificial Drainage (1992) and Irrigation (1997). U.S. Geological Survey Digital Data Series DS-491-01. http://water.usgs.gov/GIS/metadata/usgswrd/XML/mrb_e2rf1_adrain.xml, *accessed* June 27, 2011.

Wieczorek, M.E. and A.E. LaMotte, 2010b. Attributes for MRB_E2RF1 Catchments By Major River Basins in the Conterminous United States: Basin Characteristics, 2002. U.S. Geological Survey Digital Data Series DS-491-03. http://water.usgs.gov/GIS/metadata/usgswrd/XML/mrb_e2rf1_bchar.xml, *accessed* June 27, 2011.

Wieczorek, M.E. and A.E. LaMotte, 2010c. Attributes for MRB_E2RF1 Catchments By Major River Basins in the Conterminous United States: NLCD 2001 Land Use and Land Cover. U.S. Geological Survey Digital Data Series DS-491-15. http://water.usgs.gov/GIS/metadata/usgswrd/XML/mrb_e2rf1_nlcd01.xml, *accessed* June 27, 2011.

Wieczorek, M.E. and A.E. LaMotte, 2010d. Attributes for MRB_E2RF1 Catchments By Major River Basins in the Conterminous United States: Normalized Atmospheric Deposition for 2002, Total Inorganic Nitrogen. U.S. Geological Survey Digital Data Series DS-491-27. http://water.usgs.gov/GIS/metadata/usgswrd/XML/mrb_e2rf1_tin.xml, *accessed* June 27, 2011.

Wieczorek, M.E. and A.E. LaMotte, 2010e. Attributes for MRB_E2RF1 Catchments By Major River Basins in the Conterminous United States: Nutrient Inputs From Farm Fertilizer and Manure, Nitrogen and Phosphorus (N&P), 2002. U.S. Geological Survey Digital Data Series DS-491-17. http://water.usgs.gov/GIS/metadata/usgswrd/XML/mrb_e2rf1_nutrients.xml, *accessed* June 27, 2011.

Wieczorek, M.E. and A.E. LaMotte, 2010f. Attributes for MRB_E2RF1 Catchments By Major River Basins in the Conterminous United States: STATSGO Soil Characteristics. U.S. Geological Survey Digital Data Series DS-491-26. http://water.usgs.gov/GIS/metadata/usgswrd/XML/mrb_e2rf1_statsgo.xml, *accessed* June 27, 2011.

Wieczorek, M.E. and A.E. LaMotte, 2010g. Attributes for MRB_E2RF1 Catchments By Major River Basins in the Conterminous United States: Surficial Geology. U.S. Geological Survey Digital Data Series DS-491-25. http://water.usgs.gov/GIS/metadata/usgswrd/XML/mrb_e2rf1_sgeol.xml, *accessed* June 27, 2011.

Wieczorek, M.E. and A.E. LaMotte, 2010h. Attributes for MRB_E2RF1 Catchments By Major River Basins in the Conterminous United States: 30-Year Average Annual Maximum Temperature, 1972-2000. U.S. Geological Survey Digital Data Series DS-491-29. http://water.usgs.gov/GIS/metadata/usgswrd/XML/mrb_e2rf1_tmax30yr.xml, *accessed* June 27, 2011.

Wieczorek, M.E. and A.E. LaMotte, 2010i. Attributes for MRB_E2RF1 Catchments By Major River Basins in the Conterminous United States: 30-Year Average Annual Minimum Temperature, 1972-2000. U.S. Geological Survey Digital Data Series DS-491-31. http://water.usgs.gov/GIS/metadata/usgswrd/XML/mrb_e2rf1_tmin30yr.xml, *accessed* June 27, 2011.

Wieczorek, M.E. and A.E. LaMotte, 2010j. Attributes for MRB_E2RF1 Catchments By Major River Basins in the Conterminous United States: 30-Year Average Annual Precipitation, 1972-2000. U.S. Geological Survey Digital Data Series DS-491-21. http://water.usgs.gov/GIS/metadata/usgswrd/XML/mrb_e2rf1_ppt30yr.xml, *accessed* June 27, 2011.

Wieczorek, M.E. and A.E. LaMotte, 2011. Attributes for MRB_E2RF1 Catchments by Major River Basins in the Conterminous United States. U.S. Geological Survey Digital Data Series DS-491. http://water.usgs.gov/nawqa/modeling/rf1attributes.html, *accessed* June 27, 2011.

Wolock, D.M., 1997. STATSGO Soil Characteristics for the Conterminous United States. U.S. Geological Survey Open-File Report 656. http://water.usgs.gov/GIS/metadata/usgswrd/XML/muid.xml, *accessed* June 27, 2011.

Appendix

Appendix S1. Source shares (in percent of load) in the total catchment of the major subbasins. Source shares correspond to the data in Figure 7a in main manuscript. [90% prediction interval, PI, determined from parametric bootstrap analysis[1].]

Major subbasin	Developed land [90% PI]	Point sources [90% PI]	Farm fertilizer [90% PI]	Manure [90% PI]	Atmos-pheric deposition [90% PI]	Stream channels [90% PI]
**Total nitrogen**						
Upper Missouri	15 (7.7 - 90)	9 (5.4 - 17)	32 (22 - 69)	29 (17 - 100)	16 (8.1 - 100)	--
Yellowstone	9 (4.8 - 61)	13 (8.4 - 28)	18 (10 - 44)	38 (22 - 100)	22 (11 - 100)	--
Middle Missouri	10 (4.6 - 57)	5 (2.8 - 9.4)	50 (37 - 97)	27 (15 - 100)	8 (3.9 - 100)	--
Platte	10 (4.8 - 50)	27 (19 - 54)	36 (26 - 71)	20 (12 - 83)	7 (3.4 - 100)	--
Lower Middle Missouri	10 (4.8 - 62)	1 (0.8 - 2.8)	59 (47 - 100)	20 (9.7 - 100)	9 (4.6 - 100)	--
Kansas	14 (7.1 - 78)	6 (3.6 - 13)	50 (37 - 94)	19 (9.9 - 92)	10 (4.9 - 100)	--
Lower Missouri	14 (7.1 - 81)	4 (2.4 - 8.0)	39 (27 - 82)	31 (18 - 100)	12 (5.8 - 100)	--
**Total phosphorus**						
Upper Missouri	6 (3.3 - 26)	2 (1.3 - 4.8)	14 (6.6 - 100)	15 (7.5 - 41)	--	63 (53 - 85)
Yellowstone	3 (1.4 - 11)	4 (2.3 - 8.3)	5 (1.9 - 50)	18 (11 - 41)	--	70 (62 - 92)
Middle Missouri	8 (4.6 - 28)	11 (6.6 - 20)	31 (19 - 100)	24 (14 - 71)	--	27 (21 - 39)
Platte	5 (2.8 - 17)	39 (29 - 65)	17 (11 - 100)	12 (6.5 - 33)	--	27 (20 - 45)
Lower Middle Missouri	12 (7.1 - 43)	1 (0.7 - 2.5)	44 (30 - 100)	22 (11 - 69)	--	20 (14 - 31)
Kansas	16 (9.6 - 55)	13 (7.6 - 23)	33 (20 - 100)	24 (13 - 70)	--	15 (11 - 23)
Lower Missouri	16 (9.8 - 54)	3 (1.6 - 5.7)	32 (18 - 100)	32 (19 - 89)	--	18 (13 - 28)

[1] The bootstrap methodology used to compute prediction intervals does not constrain the intervals for share estimates to be less than or equal to 100 percent. For prediction intervals involving share variables in which the upper bound is estimated by the bootstrap to exceed 100 percent, the upper bound is constrained to equal 100 percent. (G.E. Schwarz, U.S. Geological Survey, personal commun., Oct. 2009).
